# Supplementary material for: Discovery of Barakacin and Its Derivatives as Novel Antiviral and Fungicidal Agents
Source: Molecules. 2023 Mar 29;28(7):3032. doi: 10.3390/molecules28073032 (PMC10095642; doi:10.3390/molecules28073032)

Supplementary materials

Discovery of Barakacin and Its Derivatives as Novel Antiviral and Fungicidal Agents

Yongyue Gao 1, Xingxing He 1, Lili Yan 1, Hongyu Zhang 1, Sijia Liu 1, Qian Ma 1, Peiyao Zhang 1, Yan Zhang 1, Zijun Zhang 1, Ziwen Wang 1,*, Aidang Lu 2 and Qingmin Wang 3,*

1 Tianjin Key Laboratory of Structure and Performance for Functional Molecules, College of Chemistry, Tianjin Normal University, Tianjin 300387, China

2 School of Chemical Engineering and Technology, Hebei University of Technology, Hebei Collaborative Innovation Center of Modern Marine Chemical Technology, Tianjin 300130, China

3 State Key Laboratory of Elemento-Organic Chemistry, Research Institute of Elemento-Organic Chemistry, College of Chemistry, Collaborative Innovation Center of Chemical Science and Engineering (Tianjin), Nankai University, Tianjin 300071, China

* Correspondence: hxxywzw@tjnu.edu.cn (Z.W.); wangqm@nankai.edu.cn (Q.W.); Tel./Fax: +86-22-2376-6531 (Z.W.); +86-22-2350-3952 (Q.W.)

Contents

1. Scheme S1. Synthesis of barakacin. S3

2. The synthetic procedures of derivatives **8a−8b, 9a−9e, 10−12, 13a−13d, 14a−14g, 15, 16a−16c** S3

3. Biological Assay S13

4. Reference: S17

5. Spectra of all target compounds, intermediates S18

1. Scheme S1. Synthesis of barakacin

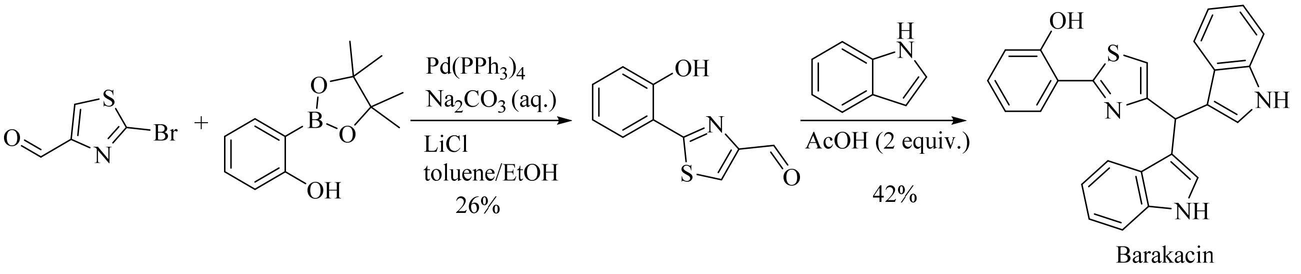

Scheme S1. Synthesis of alkaloid barakacin by Buzid's group

2. The synthetic procedures of derivatives 8a−8b, 9a−9e, 10−12, 13a−13d, 14a−14g, 15, 16a−16c

2.1. Preparation of Compounds **8a**−**8b**.

To a round bottom flask was added compound **7** (0.50 g, 1.19 mmol), K_2CO_3 (0.33 g, 2.37 mmol), and DMF (10 mL). Then corresponding bromide (2.37 mmol) was added, and the mixture was stirred at 120 °C for 12 h. After the reaction was completed, cold water (100 mL) was added. The reaction solution was partitioned and extracted with ethyl acetate (50 mL × 3), the organic phase was washed with brine (100 mL × 3), dried over anhydrous Na_2SO_4, filtered with suction, and concentrated. The residue was subjected to column chromatography with petroleum ether and ethyl acetate (2:1, v/v) as eluent to obtain compounds **8a**−**8b**.

4-(Di(1*H*-indol-3-yl)methyl)-2-(2-(prop-2-yn-1-yloxy)phenyl)thiazole (**8a**).

Brown solid, 35% yield, mp. 272−274 °C; ^{1}H NMR (400 MHz, DMSO-d_6) δ 10.84 (s, 2H), 8.26 (dd, J = 7.8, 1.5 Hz, 1H), 7.47 (d, J = 7.9 Hz, 2H), 7.44 – 7.41 (m, 1H), 7.34 (d, J = 8.7 Hz, 3H), 7.28 (d, J = 8.5 Hz, 1H), 7.11 (t, J = 7.6 Hz, 1H), 7.04 (dd, J = 9.8, 5.0 Hz, 4H), 6.89 (t, J = 7.5 Hz, 2H), 6.08 (s, 1H), 5.06 (d, J = 2.3 Hz, 2H), 3.64 (t, J = 2.3 Hz, 1H). ^{13}C NMR (100 MHz, DMSO-d_6) δ 167.5, 160.5, 159.2, 154.4, 136.9, 132.0, 129.1, 127.0, 124.0, 121.3, 119.6, 118.6, 117.5, 111.9, 79.2, 65.5, 60.2, 56.7, 30.5, 19.1, 14.0. HRMS (ESI) calcd for $C_{29}H_{22}N_3OS$ $[M+H]^+$ 460.1478, found 460.1485.

2-(2-(Benzyloxy)phenyl)-4-(di(1*H*-indol-3-yl)methyl)thiazole (**8b**).

Light yellow solid, 40% yield, mp. 150−152 °C; ^{1}H NMR (400 MHz, DMSO-d_6) δ 10.83 (s, 2H), 8.30 – 8.24 (m, 1H), 7.54 (d, J = 7.2 Hz, 2H), 7.46 (d, J = 7.9 Hz, 2H), 7.39 (t, J = 7.3 Hz, 3H), 7.32 (dd, J = 13.0, 7.9 Hz, 5H), 7.09 – 6.99 (m, 5H), 6.88 (t, J = 7.5 Hz, 2H), 6.06 (s, 1H), 5.37 (s, 2H). ^{13}C NMR (100 MHz, DMSO-d_6) δ 169.5, 157.3, 154.0, 136.7, 136.5, 129.7, 128.9, 128.5, 127.6, 127.4, 127.1, 123.0, 122.1, 121.7, 121.5, 119.8, 118.8, 116.6, 115.6, 112.1, 111.1, 71.1, 53.6. HRMS (ESI) calcd for $C_{33}H_{26}N_3OS$ [M+H]$^+$ 512.1791, found 512.1797.

2.2. Preparation of Compounds **9a**−**9e**.

In a 50 mL round-bottom flask, compound **6** was dissolved (1.00 g, 2.3 mmol) in DMF (10 mL), then NaH (0.165 g, 6.9 mmol) was slowly added to the solution in an ice bath. The result mixture was stirred for 1 h, and added corresponding alkyl chloride or acyl chloride (9.2 mmol), then stirred for 24 h. The reaction was quenched by adding cold water (100 mL), extracted with ethyl acetate (30 mL × 3). The organic phase was washed with brine (100 mL), dried over anhydrous Na_2SO_4, filtered, and concentrated. The residue was purified by column chromatography with petroleum ether and ethyl acetate (8:1, v/v) as eluent to obtain compounds **9a**−**9e**.

4-(Bis(1-methyl-1*H*-indol-3-yl)methyl)-2-(2-methoxyphenyl)thiazole (**9a**).

Light red solid, 75% yield, mp. 272−274 °C; ^{1}H NMR (400 MHz, DMSO-d_6) δ 8.35 – 8.25 (m, 1H), 7.53 (d, J = 7.9 Hz, 2H), 7.50 – 7.46 (m, 1H), 7.43 (d, J = 8.2 Hz, 2H), 7.37 (s, 1H), 7.28 (d, J = 4.7 Hz, 1H), 7.15 (dd, J = 14.3, 7.1 Hz, 3H), 7.08 (s, 2H), 6.99 (t, J = 7.5 Hz, 2H), 6.14 (s, 1H), 4.04 (s, 3H), 3.77 (s, 6H). ^{13}C NMR (100 MHz, DMSO-d_6) δ 160.9, 159.0, 156.5, 137.3, 131.2, 128.2, 127.3, 122.1, 121.4, 119.7, 118.8, 116.8, 116.5, 112.7, 110.1, 56.3, 36.6, 32.8. HRMS (ESI) calcd for $C_{29}H_{26}N_3OS$ [M+H]$^+$ 464.1791, found 464.1798.

5-(Bis(1-benzyl-1*H*-indol-3-yl)methyl)-2-(2-methoxyphenyl)thiazole (**9b**).

Orange solid, 48% yield, mp. 170−172 °C; ^{1}H NMR (400 MHz, DMSO-d_6) δ 8.22 (dd, J = 7.8, 1.7 Hz, 1H), 7.49 (d, J = 7.9 Hz, 2H), 7.46 – 7.40 (m, 1H), 7.36 (d, J = 8.2 Hz, 2H), 7.31 (s, 1H),

7.28 – 7.18 (m, 9H), 7.15 – 7.10 (m, 4H), 7.05 (dt, *J* = 14.4, 7.2 Hz, 3H), 6.91 (t, *J* = 7.5 Hz, 2H), 6.12 (s, 1H), 5.37 (s, 4H), 3.98 (s, 3H). ^{13}C NMR (100 MHz, DMSO-*d*$_6$) δ 160.5, 158.1, 156.0, 138.5, 136.3, 130.7, 128.4, 127.6, 127.2, 126.8, 121.7, 121.1, 120.8, 119.6, 118.5, 116.8, 116.1, 112.2, 110.1, 55.8, 48.8, 36.4. HRMS (ESI) calcd for $C_{41}H_{34}N_3OS$ [M+H]$^+$ 616.2417, found 616.2413.

4-(Bis(1-tosyl-1*H*-indol-3-yl)methyl)-2-(2-methoxyphenyl)thiazole (**9c**).

Light yellow solid, 51% yield, mp. 164−166 °C; ^{1}H NMR (400 MHz, CDCl$_3$) δ 8.98 (d, *J* = 7.8 Hz, 1H), 7.97 (d, *J* = 8.3 Hz, 2H), 7.65 – 7.51 (m, 5H), 7.36 – 7.27 (m, 5H), 7.25 – 7.21 (m, 1H), 7.17 (d, *J* = 8.0 Hz, 6H), 7.13 – 7.07 (m, 3H), 7.05 (s, 1H), 4.09 (s, 3H), 2.34 (s, 6H). ^{13}C NMR (100 MHz, DMSO-*d*$_6$) δ 161.3, 156.1, 154.1, 145.4, 134.8, 133.8, 131.1, 130.9, 130.2, 129.6, 127.3, 126.5, 124.9, 123.6, 123.3, 121.5, 121.2, 120.5, 117.6, 113.4, 112.4, 55.9, 35.5, 21.0. HRMS (ESI) calcd for $C_{41}H_{34}N_3O_5S_3$ [M+H]$^+$ 744.1655, found 744.1658.

(3,3'-((2-(2-Methoxyphenyl)thiazol-4-yl)methylene)bis(1*H*-indole-3,1-diyl))bis(phenylmethanone) (**9d**).

Light red solid, 42% yield, mp. 112−114 °C; ^{1}H NMR (400 MHz, DMSO-*d*$_6$) δ 8.24 (d, *J* = 8.0 Hz, 2H), 8.04 (d, *J* = 6.7 Hz, 1H), 7.75 (d, *J* = 7.6 Hz, 2H), 7.66 (dd, *J* = 16.6, 7.7 Hz, 6H), 7.60 (s, 1H), 7.54 – 7.43 (m, 5H), 7.40 – 7.27 (m, 6H), 7.22 (d, *J* = 8.3 Hz, 1H), 7.10 (t, *J* = 7.5 Hz, 1H), 6.19 (s, 1H), 3.96 (s, 3H). ^{13}C NMR (100 MHz, DMSO-*d*$_6$) δ 168.5, 168.2, 167.9, 161.1, 156.0, 154.7, 136.8, 135.8, 134.0, 132.0, 131.0, 129.6, 128.9, 128.5, 127.3, 126.2, 124.8, 123.6, 122.6, 122.0, 120.9, 120.6, 120.1, 117.4, 115.8, 113.5, 113.0, 112.2, 55.8, 54.9, 51.7, 50.9, 48.6, 35.1, 32.4, 30.3, 26.4. HRMS (ESI) calcd for $C_{41}H_{30}N_3O_3S$ [M+H]$^+$ 644.2002, found 644.2004.

4-(Bis(1-(ethylsulfonyl)-1*H*-indol-3-yl)methyl)-2-(2-methoxyphenyl)thiazole (**9e**).

Yellow solid, yield 50%, mp. 143−145 °C; ^{1}H NMR (400 MHz, DMSO-*d*$_6$) δ 8.20 (dd, *J* = 7.8, 1.6 Hz, 1H), 7.82 (d, *J* = 8.3 Hz, 2H), 7.67 (d, *J* = 7.8 Hz, 2H), 7.54 (s, 1H), 7.49 – 7.41 (m, 1H), 7.39 – 7.30 (m, 4H), 7.25 (dd, *J* = 8.3, 5.9 Hz, 3H), 7.08 (t, *J* = 7.6 Hz, 1H), 6.25 (s, 1H), 4.00 (s,

3H), 3.58 (q, J = 7.3 Hz, 4H), 1.00 (t, J = 7.3 Hz, 6H). ^{13}C NMR (100 MHz, DMSO-d_6) δ 161.2, 156.1, 154.6, 135.0, 131.1, 129.2, 127.3, 125.1, 124.7, 122.9, 121.6, 121.3, 120.9, 120.5, 117.5, 113.1, 112.3, 55.9, 48.0, 35.4. HRMS (ESI) calcd for $C_{31}H_{30}N_3O_5S_3$ $[M+H]^+$ 620.1342, found 620.1347.

2.3. Preparation of Ethyl 2-phenylthiazole-4-carboxylate (**10**).

To a stirred solution of thiobenzamide (1.00 g, 7.30 mmol) in CH_3OH (100 mL) was added ethyl bromopyruvate (30.69 g, 0.18 mol) at 0 °C. The reaction mixture was stirred at 90 °C for 2 h, cold to room temperature, and extracted with ethyl acetate (100 mL × 3). The combined organic phase was dried over anhydrous Na_2SO_4, filtered, and concentrated. The residue was purified by flash chromatography on a silica gel using petroleum ether and ethyl acetate (4:1, v/v) as eluent to give compound **10** as a yellow oil, 85% yield. ^1H NMR (400 MHz, $CDCl_3$) δ 8.16 (s, 1H), 8.06 – 7.98 (m, 2H), 7.52 – 7.42 (m, 3H), 4.45 (q, J = 7.1 Hz, 2H), 1.44 (t, J = 7.1 Hz, 3H). ^{13}C NMR (100 MHz, $CDCl_3$) δ 168.8, 161.4, 148.0, 132.7, 130.7, 128.9, 127.1, 126.9, 61.4, 14.4.

2.4. Preparation of (2-Phenylthiazol-4-yl)methanol (**11**).

To a stirred solution of $LiAlH_4$ (0.163 g, 4.29 mmol) in THF (20 mL) was added compound **10** (1.00 g, 4.29 mmol) in THF (10 mL) at 0 °C. The reaction mixture was stirred at 25 °C for 4 h, then cooled to 0 °C, and slowly added potassium sodium tartrate solution (5 mL), extracted with ethyl acetate (20 mL × 3). The combined organic phase was dried over anhydrous Na_2SO_4, filtered, and concentrated. The residue was purified by flash chromatography on a silica gel using petroleum ether and ethyl acetate (4:1, v/v) as eluent to give compound **11** as a yellow solid, 90% yield. ^1H NMR (400 MHz, DMSO-d_6) δ 7.96 – 7.89 (m, 2H), 7.56 – 7.44 (m, 4H), 5.40 (t, J = 5.7 Hz, 1H), 4.63 (dd, J = 5.7, 0.9 Hz, 2H). ^{13}C NMR (100 MHz, DMSO-d_6) δ 166.8, 159.0, 133.2, 130.1, 129.2, 126.0, 114.9, 59.8.

2.5. Preparation of 2-Phenylthiazole-4-carbaldehyde (**12**).

To a stirred solution of PCC (1.72 g, 5.30 mmol) in DCM (30 mL) was added compound **11** (1.00 g, 5.30 mmol) in DCM (30 mL) at room temperature. The reaction mixture was stirred at 25 °C for 4 h, extracted with DCM (20 mL × 3). The combined organic phase was dried over anhydrous Na_2SO_4, filtered, and concentrated. The residue was purified by flash chromatography on a silica gel using petroleum ether and ethyl acetate (4:1, v/v) as eluent to give compound **12** as a brown solid, 80% yield. ^{1}H NMR (400 MHz, DMSO-d_6) δ 10.01 (s, 1H), 8.79 (s, 1H), 8.07 – 7.97 (m, 2H), 7.60 – 7.52 (m, 3H). ^{13}C NMR (100 MHz, DMSO-d_6) δ 185.0, 168.6, 155.2, 132.2, 131.8, 131.0, 129.4, 126.6.

2.6. Preparation of Compounds **13a**−**13d**.

To a stirred solution of **12** (1 mmol) in MeCN (25 mL) was added indole or its derivatives (2 mmol) at room temperature. The reaction mixture was stirred at 25 °C for 1 h, added I_2 (1.0 mmol), then stirred at 25 °C for 2 h, quenched by adding $Na_2S_2O_3$ (50 mL) for 15 min, extracted with ethyl acetate (20 mL × 3). The combined organic phase was dried over anhydrous Na_2SO_4, filtered, and concentrated. The residue was recrystallized in DCM and petroleum ether to give compounds **13a**−**13d**.

4-(Di(1*H*-indol-3-yl)methyl)-2-phenylthiazole (**13a**).

Brown solid, 91% yield, mp. 226−228 °C; ^{1}H NMR (400 MHz, DMSO-d_6) δ 10.84 (s, 2H), 7.94 – 7.86 (m, 2H), 7.52 – 7.42 (m, 5H), 7.35 (d, *J* = 8.1 Hz, 2H), 7.29 (s, 1H), 7.09 – 7.00 (m, 4H), 6.90 (t, *J* = 7.4 Hz, 2H), 6.08 (s, 1H). ^{13}C NMR (100 MHz, DMSO-d_6) δ 166.2, 160.7, 136.4, 133.3, 130.0, 129.2, 126.5, 126.0, 123.5, 120.8, 119.1, 118.2, 116.7, 115.2, 111.5, 36.5. HRMS (ESI): Cacld for $C_{26}H_{20}N_3S$ $[M+H]^+$ 406.1372, found 406.1377.

4-(Bis(5-methoxy-1*H*-indol-3-yl)methyl)-2-phenylthiazole (**13b**).

Brown solid, 95% yield, mp. 192−194 °C; ^{1}H NMR (400 MHz, DMSO-d_6) δ 10.71 (d, *J* = 2.0 Hz, 2H), 7.97 – 7.89 (m, 2H), 7.51 – 7.42 (m, 3H), 7.36 (s, 1H), 7.25 (d, *J* = 8.7 Hz, 2H), 7.08 (d, *J* =

2.3 Hz, 2H), 6.99 (d, *J* = 2.4 Hz, 2H), 6.71 (dd, *J* = 8.7, 2.4 Hz, 2H), 6.01 (s, 1H), 3.65 (s, 6H). ^{13}C NMR (100 MHz, DMSO-d_6) δ 166.7, 161.2, 153.2, 133.8, 132.1, 130.5, 129.6, 127.3, 126.4, 124.7, 116.9, 115.7, 112.5, 111.2, 101.7, 55.7, 36.9. HRMS (ESI): Cacld for $C_{28}H_{24}N_3O_2S$ $[M+H]^+$ 466.1584, found 466.1588.

4-(Bis(6-bromo-1*H*-indol-3-yl)methyl)-2-phenylthiazole (**13c**).

Yellow solid, 85% yield, mp. 238−240 °C; ^{1}H NMR (400 MHz, DMSO-d_6) δ 11.03 (s, 2H), 7.90 (dd, *J* = 7.6, 1.8 Hz, 2H), 7.55 (d, *J* = 1.2 Hz, 2H), 7.48 (dd, *J* = 10.3, 5.1 Hz, 3H), 7.40 (d, *J* = 8.5 Hz, 2H), 7.31 (s, 1H), 7.11 (d, *J* = 1.8 Hz, 2H), 7.04 (dd, *J* = 8.5, 1.6 Hz, 2H), 6.06 (s, 1H). ^{13}C NMR (100 MHz, DMSO-d_6) δ 166.5, 160.0, 137.3, 133.2, 130.0, 129.2, 126.0, 125.4, 124.6, 121.1, 120.8, 116.7, 115.5, 114.0, 113.7, 36.2. HRMS (ESI): Cacld for $C_{26}H_{18}Br_2N_3S$ $[M+H]^+$ 466.1584, found 466.1578.

4-(Bis(5-bromo-1*H*-indol-3-yl)methyl)-2-phenylthiazole (**13d**).

Red solid, 90% yield, mp. 142−144 °C; ^{1}H NMR (400 MHz, DMSO-d_6) δ 11.12 (s, 2H), 7.99 – 7.91 (m, 2H), 7.75 (s, 2H), 7.47 (d, *J* = 6.6 Hz, 3H), 7.41 (s, 1H), 7.34 (t, *J* = 6.9 Hz, 2H), 7.21 (d, *J* = 1.7 Hz, 2H), 7.16 (dd, *J* = 8.6, 1.5 Hz, 2H), 6.08 (s, 1H). ^{13}C NMR (100 MHz, DMSO-d_6) δ 167.1, 160.3, 135.6, 133.7, 130.6, 129.7, 128.7, 126.5, 125.7, 123.8, 122.1, 116.7, 115.9, 114.0, 111.4, 36.6. HRMS (ESI): Cacld for $C_{26}H_{18}Br_2N_3S$ $[M+H]^+$ 466.1584, found 466.1588.

2.7. Preparation of Compounds **14a**−**14g**.

At room temperature, to a solution of indole (0.59 g, 5 mmol) in acetic acid (8 mL) was added corresponding aldehyde (2.5 mmol) under constant stirring. The result solution was stirred at room temperature for 16 h. After that, the reaction solution was slowly neutralized with NaOH (10%) in an ice bath, extracted with ethyl acetate (20 mL × 3). The combined organic phase was washed with brine (100 mL), dried over anhydrous Na_2SO_4, filtered, and concentrated. The residue was purified

by column chromatography on a silica gel using petroleum ether and ethyl acetate (5:1, v/v) as eluent to obtain compounds **14a**−**14g**.

2-(Di(1*H*-indol-3-yl)methyl)benzo[d]thiazole (**14a**).

Brown solid, 79% yield, mp. 143−145 °C; ^{1}H NMR (400 MHz, DMSO-*d*$_6$) δ 11.03 (s, 2H), 7.94 (t, *J* = 7.5 Hz, 2H), 7.45 (dd, *J* = 11.1, 4.1 Hz, 3H), 7.42 – 7.32 (m, 3H), 7.22 (s, 2H), 7.07 (t, *J* = 7.6 Hz, 2H), 6.91 (t, *J* = 7.5 Hz, 2H), 6.38 (s, 1H). ^{13}C NMR (100 MHz, DMSO-*d*$_6$) δ 176.3, 153.0, 141.5, 136.4, 135.0, 126.3, 125.8, 124.7, 124.0, 122.4, 122.0, 121.2, 118.9, 118.6, 115.2, 111.6, 56.0, 18.5. HRMS (ESI): Cacld for $C_{24}H_{18}N_3S$ $[M+H]^+$ 380.1216, found 380.1219.

3,3'-(Pyrimidin-5-ylmethylene)bis(1*H*-indole) (**14b**).

Brown solid, 71% yield, mp. 214−216 °C; ^{1}H NMR (400 MHz, DMSO-*d*$_6$) δ 11.00 (d, *J* = 1.5 Hz, 2H), 9.05 (s, 1H), 8.79 (s, 2H), 7.39 (d, *J* = 8.1 Hz, 2H), 7.34 (d, *J* = 7.9 Hz, 2H), 7.12 – 7.04 (m, 2H), 6.97 (d, *J* = 2.2 Hz, 2H), 6.91 (dd, *J* = 11.1, 3.8 Hz, 2H), 5.99 (s, 1H). ^{13}C NMR (100 MHz, DMSO-*d*$_6$) δ 157.0, 156.9, 138.5, 137.0, 126.6, 124.4, 121.7, 119.3, 119.0, 116.6, 112.1, 35.4. HRMS (ESI): Cacld for $C_{21}H_{17}N_4$ $[M+H]^+$ 325.1448, found 325.1441.

2-(Di(1*H*-indol-3-yl)methyl)-1*H*-benzo[d]imidazole (**14c**).

Light brown solid, 71% yield, mp. 296−298 °C; ^{1}H NMR (400 MHz, DMSO-*d*$_6$) δ 10.96 (d, *J* = 1.7 Hz, 2H), 7.51 – 7.33 (m, 7H), 7.16 (d, *J* = 2.2 Hz, 2H), 7.10 (dd, *J* = 6.0, 3.2 Hz, 2H), 7.07 – 7.02 (m, 2H), 6.89 (t, *J* = 7.5 Hz, 2H), 6.11 (s, 1H). ^{13}C NMR (100 MHz, DMSO-*d*$_6$) δ 157.3, 143.8, 137.2, 134.8, 128.6, 127.2, 121.9, 121.6, 121.2, 119.3, 119.1, 118.9, 114.5, 111.5, 110.2, 34.9, 32.8. HRMS (ESI): Cacld for $C_{24}H_{19}N_4$ $[M+H]^+$ 363.1604, found 363.1609.

3,3'-(Bicyclo[2.2.1]hept-5-en-2-ylmethylene)bis(1*H*-indole) (**14d**).

Light yellow solid, 76% yield, mp. 114−116 °C; ^{1}H NMR (400 MHz, DMSO-*d*$_6$) δ 10.71 (dd, *J* = 15.0, 1.5 Hz, 2H), 7.58 (d, *J* = 7.8 Hz, 1H), 7.45 (d, *J* = 7.9 Hz, 1H), 7.39 (t, *J* = 2.6 Hz, 2H), 7.27 (d, *J* = 7.9 Hz, 1H), 7.23 (d, *J* = 8.1 Hz, 1H), 6.97 – 6.92 (m, 2H), 6.82 (dd, *J* = 9.6, 8.0 Hz, 2H),

6.23 (dd, J = 5.6, 2.9 Hz, 1H), 6.06 (dd, J = 5.7, 2.9 Hz, 1H), 3.71 (d, J = 12.0 Hz, 1H), 3.27 (ddd, J = 15.9, 8.1, 3.9 Hz, 1H), 2.77 (s, 1H), 1.97 – 1.89 (m, 1H), 1.32 (t, J = 8.0 Hz, 3H), 0.59 (dd, J = 6.9, 4.7 Hz, 1H). ^{13}C NMR (100 MHz, DMSO-d_6) δ 137.5, 136.9, 136.7, 133.1, 127.6, 126.8, 122.6, 122.3, 120.9, 120.8, 119.7, 119.3, 119.1, 119.0, 118.2, 118.2, 111.7, 111.6, 49.3, 45.4, 43.5, 43.0, 33.0. HRMS (ESI): Cacld for $C_{24}H_{23}N_2$ $[M+H]^+$ 339.1856, found 339.1850.

3,3'-((1*H*-Pyrazol-4-yl)methylene)bis(1H-indole) (**14e**).

Brown solid, 82% yield, mp. 270−272 °C; ^{1}H NMR (400 MHz, DMSO-d_6) δ 12.49 (s, 1H), 10.76 (d, J = 1.0 Hz, 2H), 7.41 (s, 2H), 7.34 (dd, J = 15.2, 8.0 Hz, 4H), 7.05 – 6.94 (m, 4H), 6.85 (t, J = 7.4 Hz, 2H), 5.77 (s, 1H). ^{13}C NMR (100 MHz, DMSO-d_6) δ 137.1, 127.0, 124.6, 123.4, 121.3, 119.7, 119.3, 118.5, 111.9, 30.3. HRMS (ESI): Cacld for $C_{20}H_{17}N_4$ $[M+H]^+$ 313.1448, found 313.1444.

3,3'-((1*H*-Imidazol-2-yl)methylene)bis(1H-indole) (**14f**).

Brown solid, 77% yield, mp > 300 °C; ^{1}H NMR (400 MHz, DMSO-d$_6$) δ 11.90 (s, 1H), 10.87 (d, J = 1.5 Hz, 2H), 7.35 (dd, J = 9.5, 8.8 Hz, 4H), 7.06 – 7.00 (m, 4H), 6.91 – 6.85 (m, 4H), 5.91 (s, 1H). ^{13}C NMR (100 MHz, DMSO-d_6) δ 150.5, 136.8, 127.0, 123.9, 121.3, 119.4, 118.7, 116.4, 111.9, 34.5. HRMS (ESI): Cacld for $C_{20}H_{17}N_4$ $[M+H]^+$ 313.1448, found 313.1454.

2-(Di(1*H*-indol-3-yl)methyl)thiazole (**14g**).

Brown solid, 78% yield, mp. 147−149 °C; ^{1}H NMR (400 MHz, DMSO-d_6) δ 10.95 (s, 2H), 7.72 (d, J = 3.3 Hz, 1H), 7.51 (d, J = 3.3 Hz, 1H), 7.39 (t, J = 8.8 Hz, 4H), 7.12 (d, J = 1.7 Hz, 2H), 7.06 (t, J = 7.5 Hz, 2H), 6.90 (t, J = 7.5 Hz, 2H), 6.28 (d, J = 2.4 Hz, 1H). ^{13}C NMR (100 MHz, DMSO-d_6) δ 175.3, 142.5, 136.9, 126.7, 124.2, 121.5, 120.1, 119.4, 118.9, 116.6, 112.0, 30.8. HRMS (ESI): Cacld for $C_{20}H_{16}N_3S$ $[M+H]^+$ 330.1059, found 330.1062.

2.8. Preparation of 2-(Bis(1-methyl-1*H*-indol-3-yl)methyl)-1*H*-benzo[d]imidazole (**15**).

To a solution of indole (8.00 g, 0.068 mol) in THF (200 mL) at room temperature was added NaH (1.96 g, 0.0816 mol), and the mixture was stirred for 30 min. Then iodomethane (11.58 g, 0.0816 mol) was added. The result reaction mixture was allowed to react at room temperature for 10 h, quenched by addition of H_2O (200 mL), and extracted with ethyl acetate (200 mL × 3). The combined organic phase was dried over anhydrous Na_2SO_4, filtered, and concentrated. The residue was purified by column chromatography on a silica gel using petroleum ether and ethyl acetate (10:1, v/v) as eluent to give 1-methylindole, which was used directly for the next step. 1-Methylindole (4.62 g, 0.0352 mol) was dissolved in 50 mL of acetic acid, benzimidazole-2-carbaldehyde (2.57 g, 0.0176 mol) was added with constant stirring, and the reaction mixture was allowed to react at room temperature for 16 h. After that, the reaction solution was slowly neutralized with NaOH (10%) in an ice bath, extracted with ethyl acetate (50 mL × 3). The combined organic phase was washed with brine (100 mL), dried over anhydrous Na_2SO_4, filtered, and concentrated. The residue was purified by column chromatography on a silica gel using petroleum ether and ethyl acetate (7:1, v/v) as eluent to obtain compound **15** as a yellow solid, 84% yield, mp. 248−250 °C; ^{1}H NMR (400 MHz, DMSO-d_6) δ 11.98 (s, 1H), 7.44 (ddd, J = 19.2, 8.5, 5.7 Hz, 6H), 7.18 – 7.07 (m, 6H), 6.94 (t, J = 7.5 Hz, 2H), 6.13 (s, 1H), 3.73 (s, 6H). ^{13}C NMR (100 MHz, DMSO-d_6) δ 157.3, 137.2, 134.8, 128.6, 127.2, 121.9, 121.6, 121.2, 119.3, 119.1, 118.9, 114.5, 111.5, 110.2, 32.8. HRMS (ESI): Cacld for $C_{26}H_{23}N_4$ $[M+H]^+$ 391.1917, found 391.1911.

2.9. Preparation of Compounds **16a−16c**.

At room temperature, compound **15** (0.40 g, 1.02 mmol) was dissolved in 20 mL of *N,N*-dimethylformamide (DMF), NaH (0.114 g, 2.04 mmol) was added, and the mixture was stirred for 30 min. After that, corresponding halogenated substance (1.02 mmol) was added to the reaction mixture at room temperature, and the reaction solution was stirred continually for 8 h, then quenched by adding of H_2O (100 mL), and extracted with ethyl acetate (100 mL × 3). The combined organic phase was washed with brine (100 mL), dried over anhydrous Na_2SO_4, filtered, and concentrated.

The residue was purified by column chromatography on a silica gel using petroleum ether and ethyl acetate (10:1, v/v) as eluent to obtain compounds **16a**−**16c**.

2-(Bis(1-methyl-1*H*-indol-3-yl)methyl)-1-methyl-1*H*-benzo[d]imidazole (**16a**).

Brown solid, 70% yield, mp. 241−243 °C; ^{1}H NMR (400 MHz, $CDCl_3$) δ 7.77 (d, *J* = 7.2 Hz, 1H), 7.43 (d, *J* = 7.9 Hz, 2H), 7.35 – 7.27 (m, 3H), 7.25 (s, 1H), 7.25 – 7.17 (m, 3H), 7.02 (t, *J* = 7.5 Hz, 2H), 6.79 (s, 2H), 6.24 (s, 1H), 3.74 (s, 3H), 3.68 (s, 6H). ^{13}C NMR (100 MHz, $CDCl_3$) δ 156.2, 137.3, 128.5, 127.1, 121.7, 119.9, 119.2, 119.1, 109.4, 109.0, 33.1, 32.8, 30.3. HRMS (ESI): Cacld for $C_{27}H_{25}N_4$ $[M+H]^+$ 405.2074, found 405.2079.

2-(Bis(1-methyl-1*H*-indol-3-yl)methyl)-1-tosyl-1*H*-benzo[*d*]imidazole (**16b**).

White solid, 75% yield, mp. 135−137 °C; ^{1}H NMR (400 MHz, DMSO-*d*$_6$) δ 7.61 – 7.56 (m, 1H), 7.44 – 7.39 (m, 1H), 7.34 (d, *J* = 8.2 Hz, 2H), 7.28 (d, *J* = 7.9 Hz, 3H), 7.20 (d, *J* = 6.6 Hz, 3H), 7.14 (dd, *J* = 5.9, 3.2 Hz, 2H), 7.08 (s, 3H), 6.98 – 6.93 (m, 2H), 6.87 (t, *J* = 7.5 Hz, 2H), 6.14 (s, 1H), 5.52 (s, 2H), 3.66 (s, 6H). ^{13}C NMR (100 MHz, DMSO-*d*$_6$) δ 156.0, 142.1, 137.0, 136.7, 135.5, 128.4, 128.3, 127.3, 126.7, 126.5, 122.6, 121.9, 121.3, 121.0, 119.0, 118.9, 118.4, 113.4, 110.2, 109.6, 32.2. HRMS (ESI): Cacld for $C_{33}H_{29}N_4$ $[M+H]^+$ 481.2387, found 481.2384.

1-Benzyl-2-(bis(1-methyl-1*H*-indol-3-yl)methyl)-1*H*-benzo[d]imidazole (**16c**).

Red liquid, 65% Yield. ^{1}H NMR (400 MHz, $CDCl_3$) δ 8.19 (d, *J* = 8.2 Hz, 1H), 7.69 (d, *J* = 7.9 Hz, 1H), 7.47 (d, *J* = 7.9 Hz, 2H), 7.34 (dd, *J* = 18.0, 9.3 Hz, 4H), 7.23 (s, 1H), 7.22 – 7.17 (m, 2H), 7.03 (t, *J* = 7.3 Hz, 2H), 6.96 (s, 1H), 6.71 (d, *J* = 8.2 Hz, 2H), 6.53 (s, 2H), 3.67 (d, *J* = 2.6 Hz, 1H), 3.55 (s, 6H), 2.20 (s, 3H). ^{13}C NMR (100 MHz, DMSO-*d*$_6$) δ 155.9, 145.9, 141.3, 136.8, 134.0, 129.7, 128.4, 126.5, 125.1, 124.7, 121.2, 120.0, 118.7, 118.5, 113.8, 113.4, 109.7, 32.8, 32.2, 21.0. HRMS (ESI): Cacld for $C_{33}H_{29}N_4O_2S$ $[M+H]^+$ 545.2006, found 545.2001.

3. Biological Assay

Antiviral biological assay[1].

Purification of tobacco mosaic virus.

Using Gooding's method[2], the upper leaves of *Nicotiana tabacum* L. inoculated with TMV were selected and ground in phosphate buffer and then filtered through double-layer pledget. The filtrate was centrifuged at 10000 g, treated with PEG twice, and centrifuged again. The whole experiment was processed at 4 °C. Absorbance value was estimated at 260 nm by ultraviolet spectrophotometer.

$$Virus\ concn = (A_{260} \times dilution\ ratio) / E_{1cm}^{0.1\%,\,260nm}$$

Protective effect of compounds against TMV in vivo.

The compound solution (100 μg/mL or 500 μg/mL) was smeared on growing *Nicotiana. tabacum* L. leaves (at least 3 leaves) of the same age. In another pot, the leaves were smeared with the solvent as a control. The leaves were then inoculated with the virus (6 × 10^{-3} μg/mL) after 12 h by rubbing emery. First, spread a layer of 600 mesh emery on the tested leaves, then brush the leaves 1 to 2 times along the direction of the vein with a brush stained with TMV (6 × 10^{-3} μg/mL), and then wash the leaves with clear water. The total local lesion numbers appearing on the leaves 3−4 days after inoculation were recorded[2]. There are three replicates for each compound.

Inactivation effect of compounds against TMV in vivo.

To test viral inhibition, equal volumes of the virus (6 × 10^{-3} μg/mL) and the solution (100 μg/mL or 500 μg/mL) of synthesized compounds or controls were mixed together for 30 min. The mixture was then inoculated into the growing *N. tabacum* L leaves of the same age, and another pot was inoculated with the mixture of solvent and the virus (6 × 10^{-3} μg/mL) as the control. The local lesion numbers were recorded 3−4 days after inoculation[2]. There are three replicates for each compound.

Curative effect of compounds against TMV in vivo.

TMV (concentration of 6.0×10^{-3} μg/mL) was inoculated on the growing leaves of *N. tabacum* L. of the same age. Then, the leaves were washed with water and dried. The compound solution was smeared on the inoculated leaves, while inoculated leaves in another pot were smeared with the solvent as a control. The local lesion numbers were recorded 3−4 days after inoculation[3].

There are three replicates for each compound. The *in vivo* inhibition rates of the compound were then calculated according to the following formula ("av" means average, and controls were not treated with compound).

Inhibition rate (%) = [(av local lesion no. of control − av local lesion no. of drug-treated)/av local lesion no. of control] × 100%

Detailed bioassay procedures for the fungicidal activities[4]

In Vitro Antifungal Bioassay. The fungicidal activities of compounds were evaluated in mycelial growth tests conducted in artificial media against 8 plant pathogens at a rate of 50 μg/mL. Each test compound was dissolved in a suitable amount of acetone and diluted with water containing 0.1% TW-80 to a concentration of 500 μg/mL. To each petri dish was added 1 mL of the test solution and 9 mL of culture medium to make a 50 μg/mL concentration of the test compound, while in another petri dish was added 1 mL distilled water containing 0.1% TW-80 and 9 mL of culture medium as a blank control. A 4 mm diameter of hyphal growth was cut using a hole puncher on a growing fungal culture and the hyphae were moved to the petri dish containing the test compound. Each assay was performed three times. The dishes were stored in controlled environment cabinets (24±1°C) for 4 days, after which the diameter of mycelial growth was measured and the percentage inhibition was calculated using the following equation: *Percentage inhibition (%)* = (*averaged diameter of mycelia in blank controls – averaged diameter of mycelia in medicated tablets*) / (*averaged diameter of*

mycelia in blank controls – 4 mm) × 100.

Preliminary mechanism research

In vitro TMV rod assembly inhibition. TMV purification was performed according to the instructions by Leberman[5]. TMV RNA was purified by RNApure virus kit (CoWin Biosciences) and TMV capsid protein (TMV CP) was isolated using glacial acetic acid as described by Fraenkel-Conrat[6]. Before assembly, 20S CP Disk was prepared by incubating 23.5 mg/mL TMV CP in 0.1 M phosphate buffer (pH 7.0) at 20 °C for 12 h. After incubation, *in vitro* TMV reconstitution reactions were performed by adding 7.5 μL of phosphate buffer (0.1 M, pH 7.0), 2 μL of 20S Disk (23.5 mg/mL) and 0.5 μL of TMV RNA (2 μg/μL). The assembly reaction mixture was incubated at 20 °C for 12 h and could be then transferred into the copper grid for transmission electron microscopy (TEM) assay. The assembly reaction mixture (5 μL) was mixed with 5 μL 0.1 M phosphate buffer (pH 7.0) and dropped onto the copper film waiting for 5 minutes. After the incubation, the droplet was removed by filter paper and negatively stained by 2% phosphotungstic acid (pH 7.0) for three minutes. After removing the staining agent, the copper was placed at 37 ℃ for 2 h for drying. The morphology of the reconstituted TMV rods was imaged at 200 keV on a CCD camera. For the inhibition tests with the compounds, in vitro TMV reconstitution inhibition reactions were performed by adding 7.4 μL of phosphate buffer (0.1 M, pH 7.0), 2 μL of 20S Disk (2 mg/mL), 0.5 µL of TMV RNA (2 μg/µL) and 0.1 μL of DMSO or the compound (10 μM). All treatments were repeated over time to validate the results.

In vitro 20S CP Disk assembly inhibition. For the inhibition tests with the compounds, TMV CP was first adjusted to 23.5 mg/mL with 0.1 M phosphate buffer (pH 7.0). *In vitro* 20S CP Disk assembly reactions were performed by adding 4.9 μL of phosphate buffer (0.1 M, pH 7.0), 5 µL

TMV CP (23.5 mg/mL) and 0.1 μL DMSO or the compound (10 μM). The assembly reaction was incubated at 20 °C for 12 h. The morphology of the 20S CP Disk was imaged via TEM at 200 keV on a CCD camera. All treatments were repeated over time for confirmation.

Molecular docking research

The calculation procedures for molecular docking research consist of four steps[7].

Receptor preparation. The 3D crystal structure of TMV-CP (PDB code:1EI7) was downloaded from the protein data bank (PDB) and this was used as the receptor for molecular docking. Water molecules were removed from the target protein and hydrogen atoms were added using AutoDock Tools prior to molecular docking.

Ligand preparation. Target compounds are drawn using ChemOffice 2015 as ligands followed by management of its conformer and the minimisation process.

Molecular docking using AutoDock Vina. The input files for AutoDock Vina were prepared using AutoDock tools. The protein was placed in a grid box (grid parameters: center x = 5, center y = -20, center z = 0.8, size x = 60, size y = 60, size z = 56), using AutoDock Vina at 1.00 Å to define the binding site. The docking procedure was performed using the instructed command prompts.

Analysing and output visualisation using PyMOL.

The docking poses were ranked according to their docking scores. The scoring function in Auto Dock was used to predict the binding affinity of one ligand to the receptor molecule. The conformation with the lowest binding affinity was selected for further analysis after the docking process. The docking results included the locations of hydrogen bonds and closely interacting residues were performed by PyMOL software.

4. Reference

(1) Wang, Z. W.; Wei, P.; Wang, L. Z.; Wang, Q. M. Design, synthesis, and anti-tobacco mosaic virus (TMV) activity of phenanthroindolizidines and their analogues. *J. Agric. Food Chem.* **2012**, *60*, 10212−10219.

(2) Gooding, G. V. Jr.; Hebert, T. T. A simple technique for purification of tobacco mosaic virus in large quantities. *Phytopathology* **1967**, *57*, 1285−1290.

(3) Li, S. Z.; Wang, D. M.; Jiao, S. M. In *Pesticide Experiment Methods-Fungicide Sector*; Li, S. Z., Ed.; Agriculture Press of China: Beijing, China, **1991**; 93−94.

(4) Zhao, H. P.; Liu, Y. X.; Cui, Z. P.; Beattie, D.; Gu, Y. C.; Wang, Q. M. Design, synthesis, and biological activities of arylmethylamine substituted chlorotriazine and methylthiotriazine compounds. *J. Agric. Food Chem*. **2011**, *59*, 11711−11717.

(5) Leberman, R. Isolation of plant viruses by means of simple coacervates. *Virol* **1966**, *30*, 341–347.

(6) Fraenkel Conrat, H.; Williams, R. C. Reconstitution of active tobacco mosaic virus fromits inactive protein and nucleic acid components. *Proc. Natl. Acad. Sci.* U S A **1955**, *41*, 690–698.

(7) Seyedi, S. S.; Shukri, M.; Hassandarvish, P.; Oo, A.; Muthu, S. E.; Abubakar, S.; Zandi, K. Computational approach towards exploring potential anti-chikungunya activity of selected flavonoids. *Sci. Rep.* **2016**, *6*, 24027.

5. Spectra of all target compounds, intermediates

Figure S1. ^{1}H NMR spectrum (400 MHz, $CDCl_3$) of 1

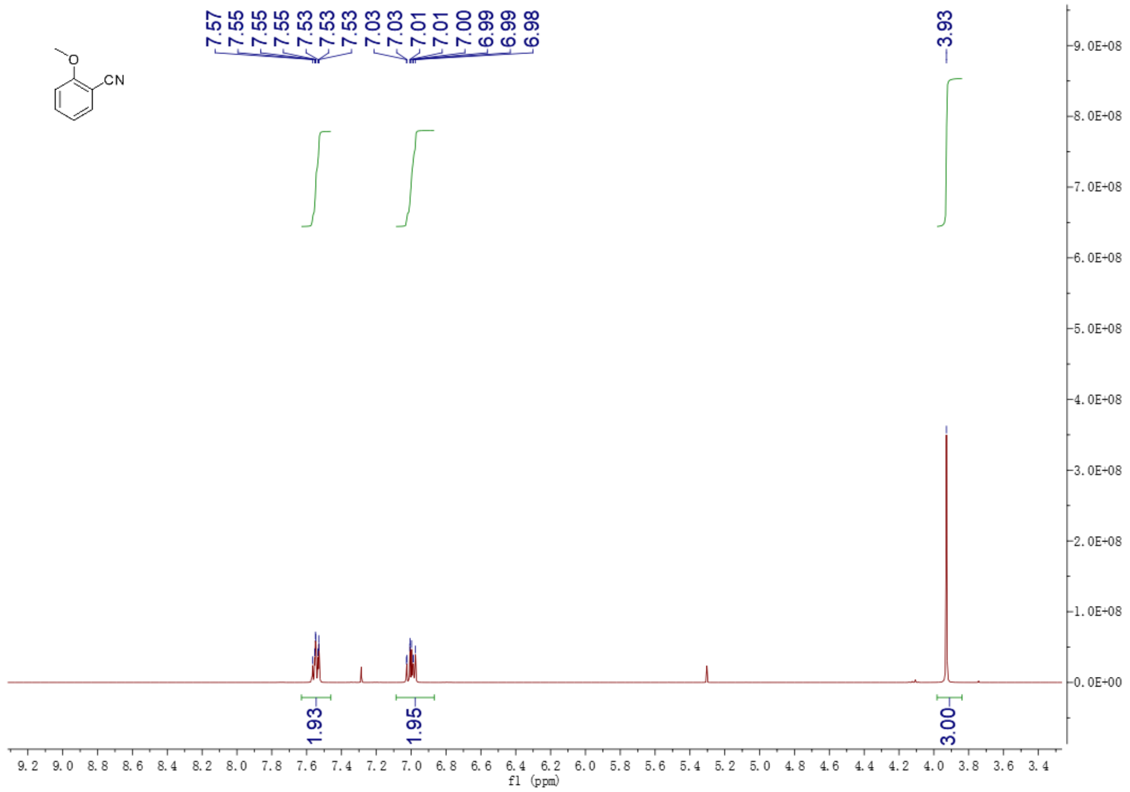

Figure S2. ^{13}C NMR spectrum (100 MHz, $CDCl_3$) of 1

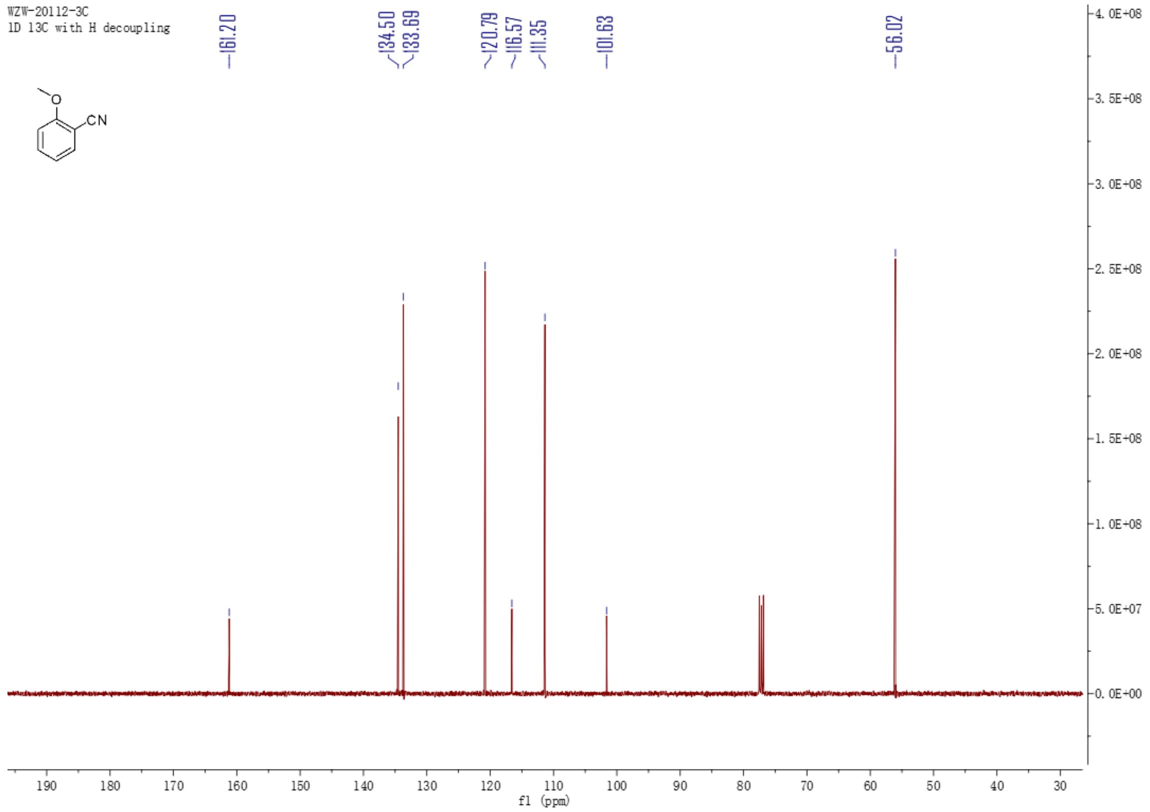

Figure S3. ^{1}H NMR spectrum (400 MHz, $CDCl_3$) of 2

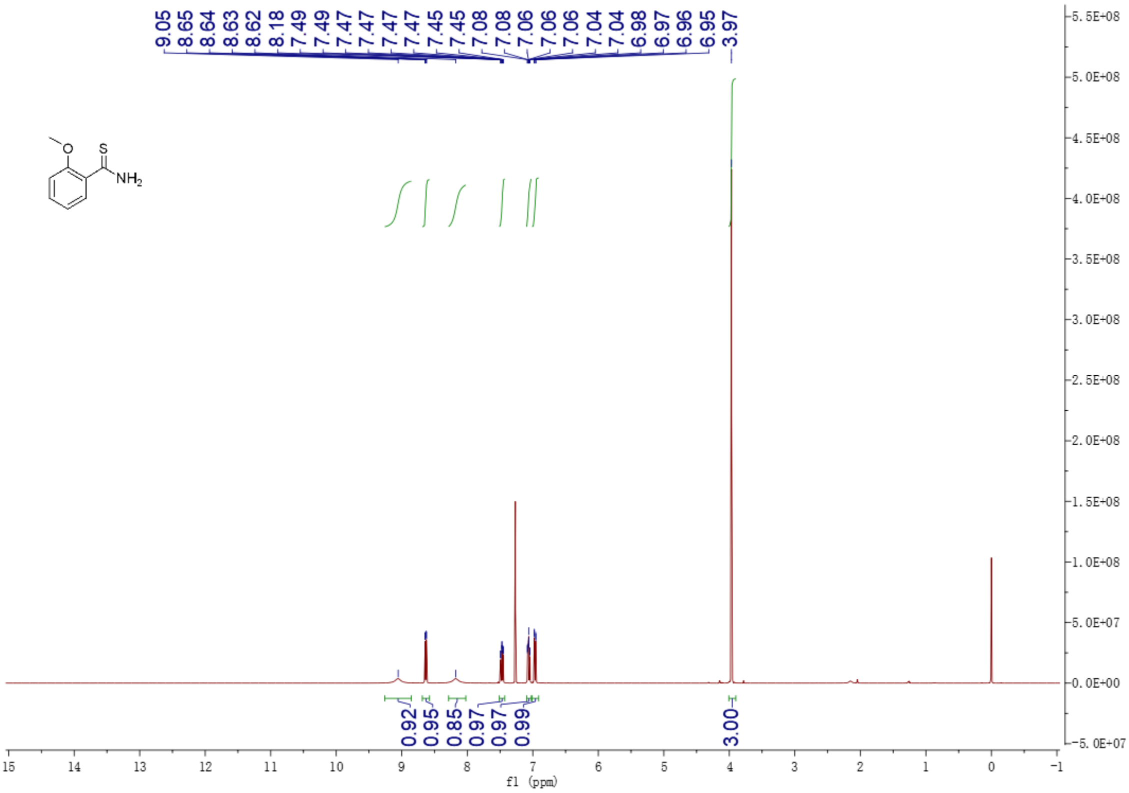

Figure S4. ^{13}C NMR spectrum (100 MHz, $CDCl_3$) of 2

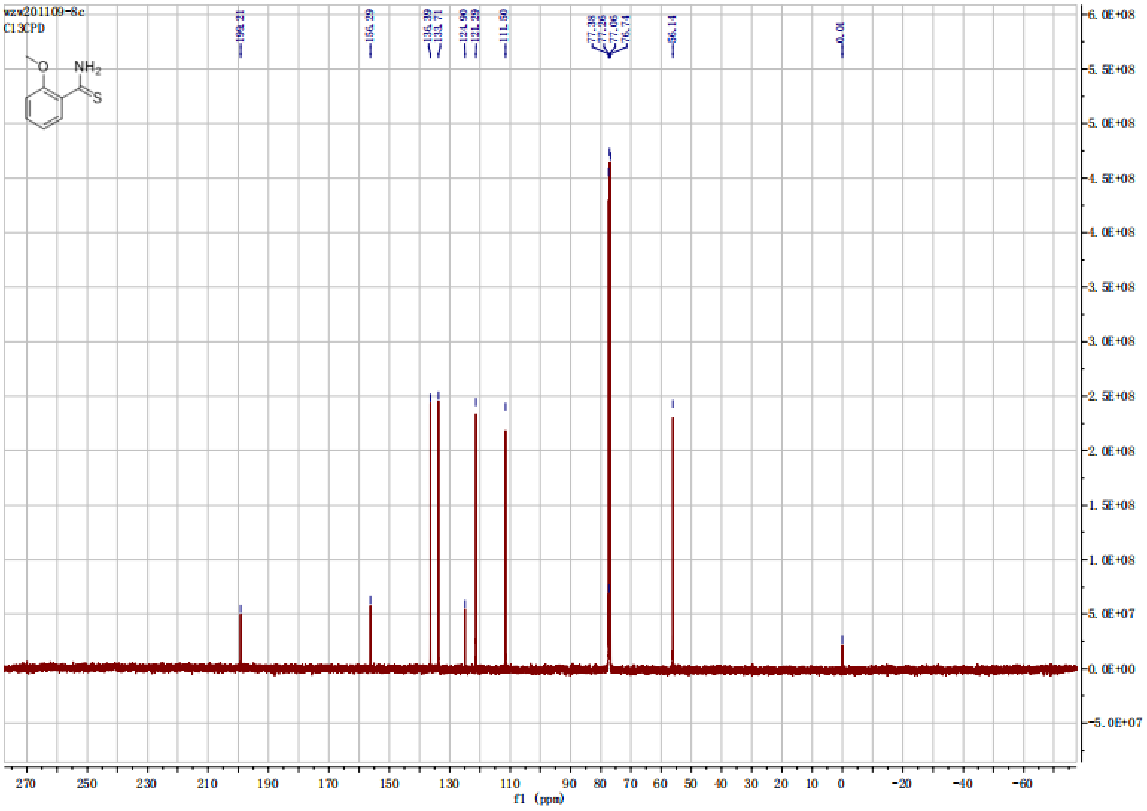

Figure S5. ^{1}H **NMR spectrum (400 MHz,** $CDCl_3$**) of 3**

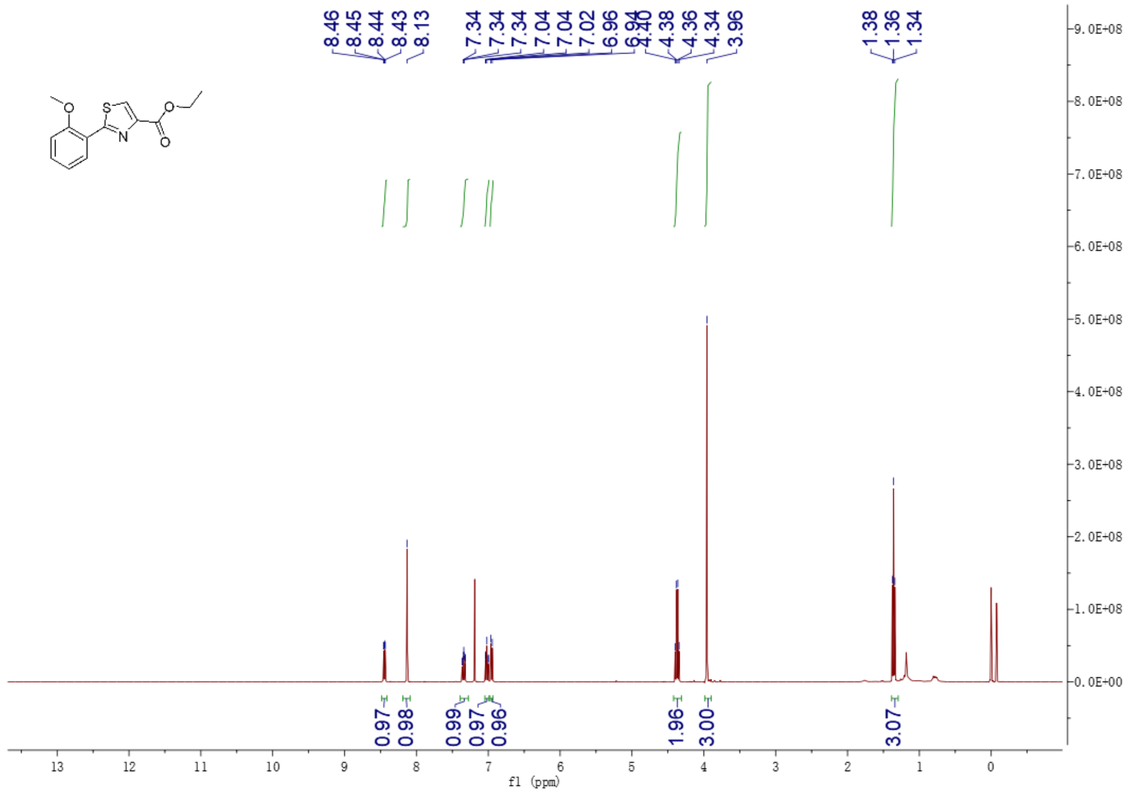

Figure S6. ^{13}C **NMR spectrum (100 MHz,** $CDCl_3$**) of 3**

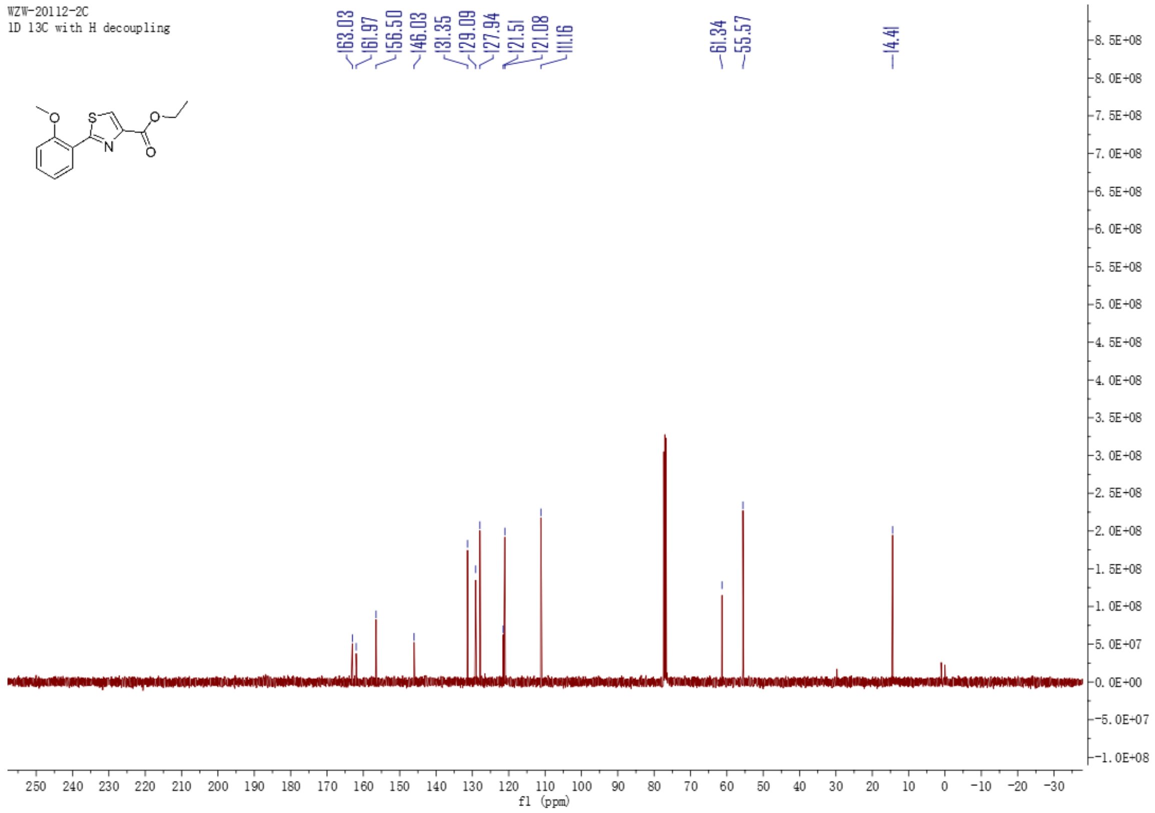

Figure S7. ^{1}H NMR spectrum (400 MHz, CDCl$_3$) of 4

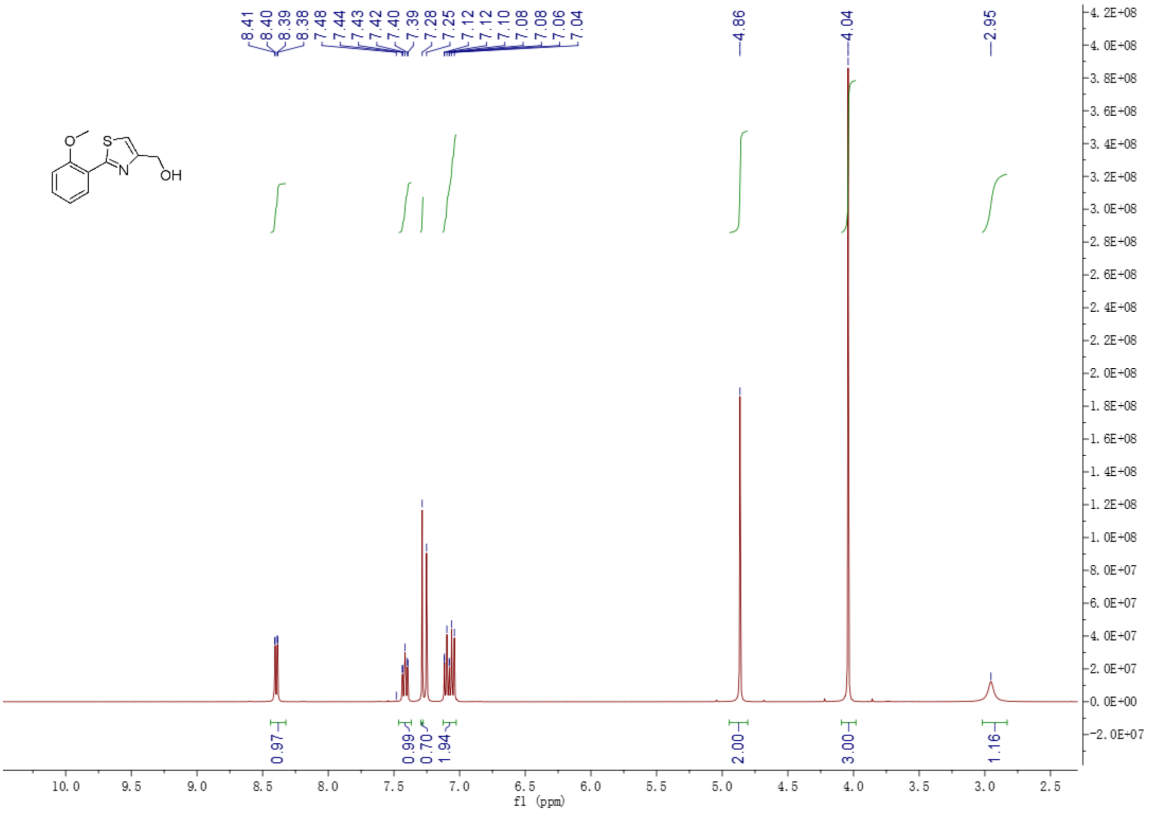

Figure S8. ^{13}C NMR spectrum (100 MHz, CDCl$_3$) of 4

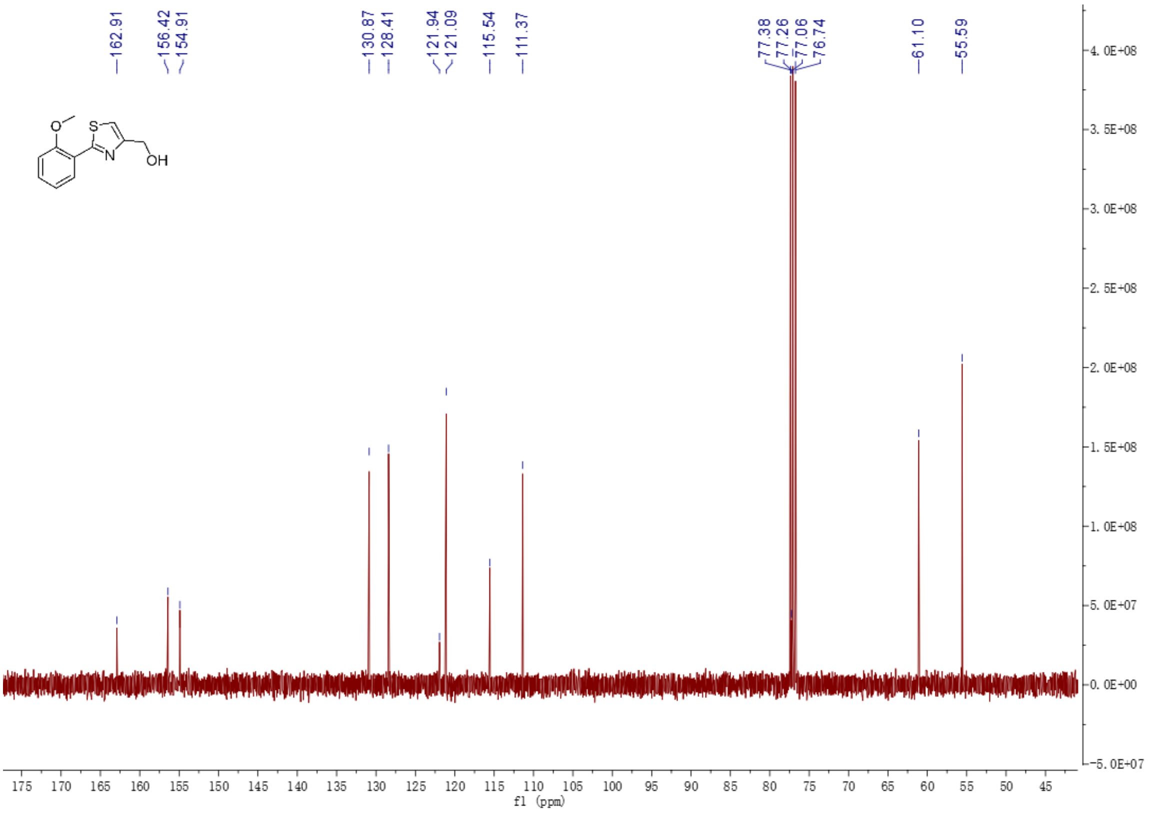

Figure S9. ^{1}H NMR spectrum (400 MHz, $CDCl_3$) of 5

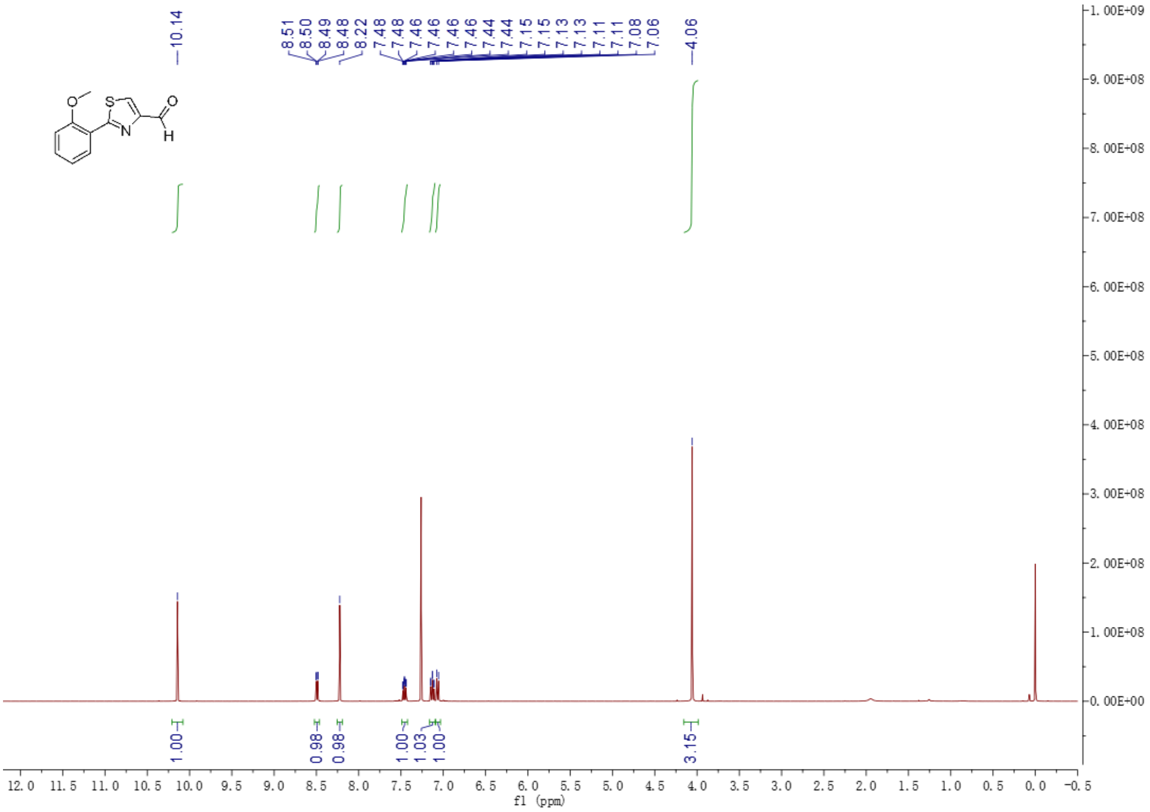

Figure S10. ^{13}C NMR spectrum (100 MHz, $CDCl_3$) of 5

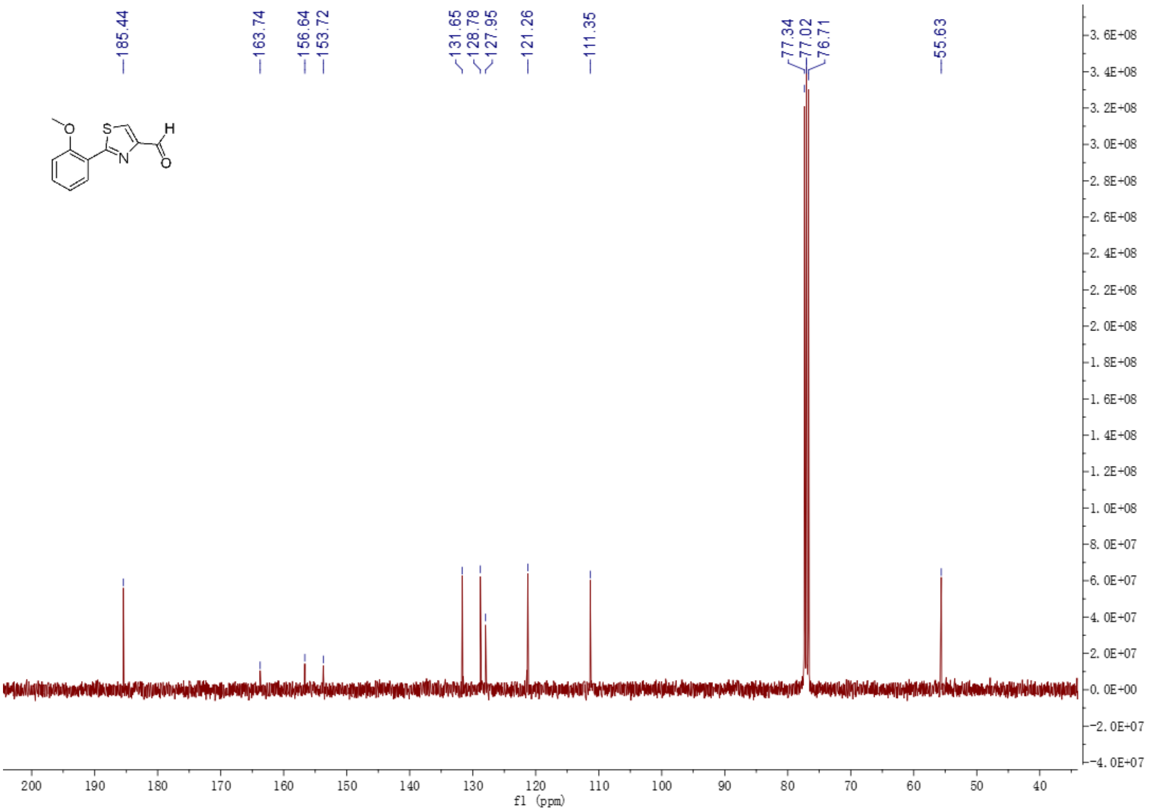

Figure S11. ^{1}H NMR spectrum (400 MHz, DMSO-d_6) of 6

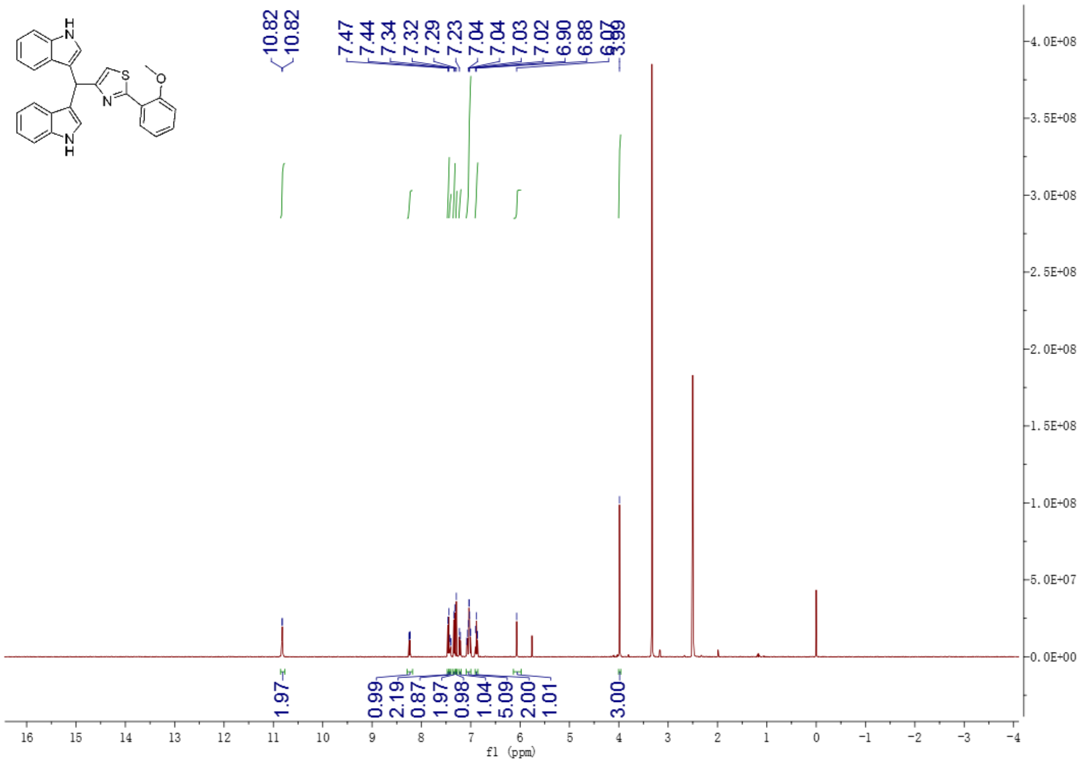

Figure S12. ^{13}C NMR spectrum (100 MHz, DMSO-d_6) of 6

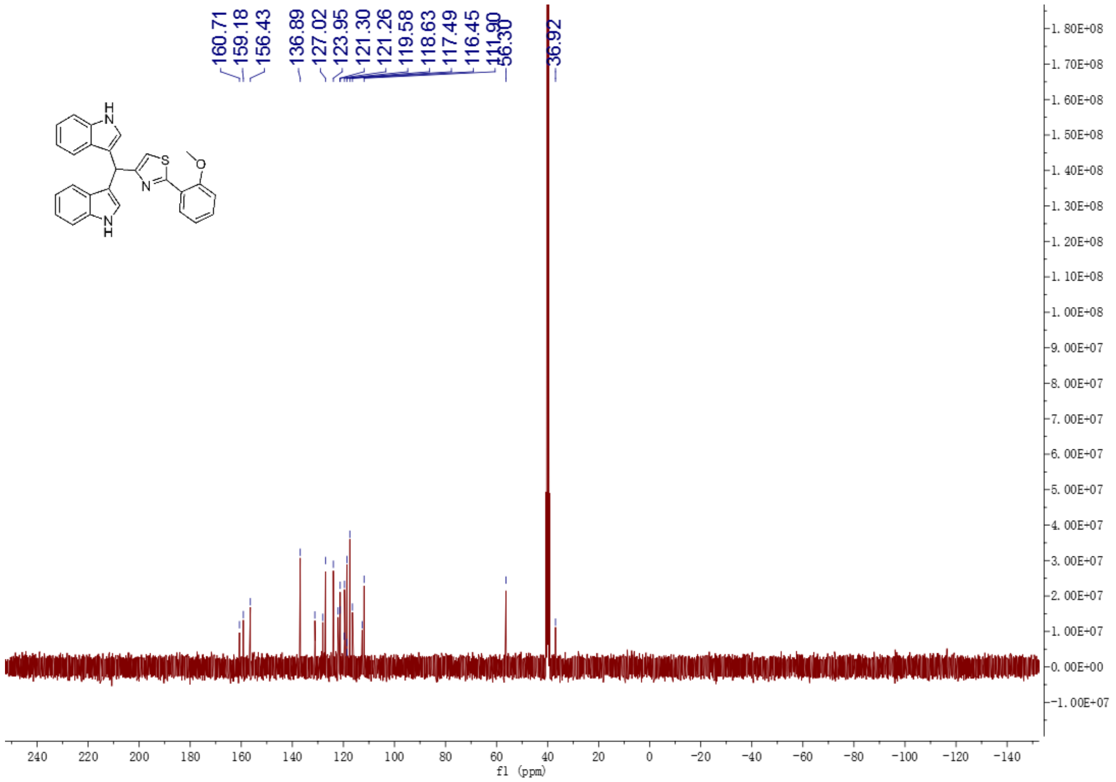

Figure S13. ^{1}H NMR spectrum (400 MHz, DMSO-d_6) of 7

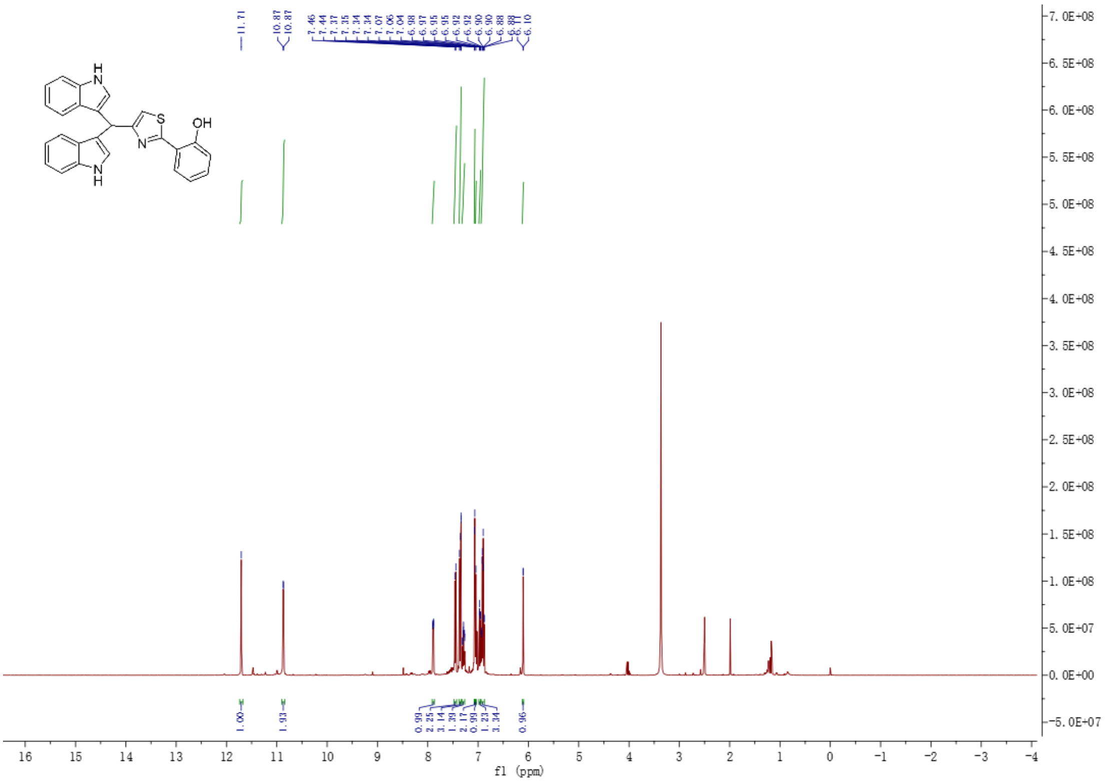

Figure S14. ^{13}C NMR spectrum (100 MHz, DMSO-d_6) of 7

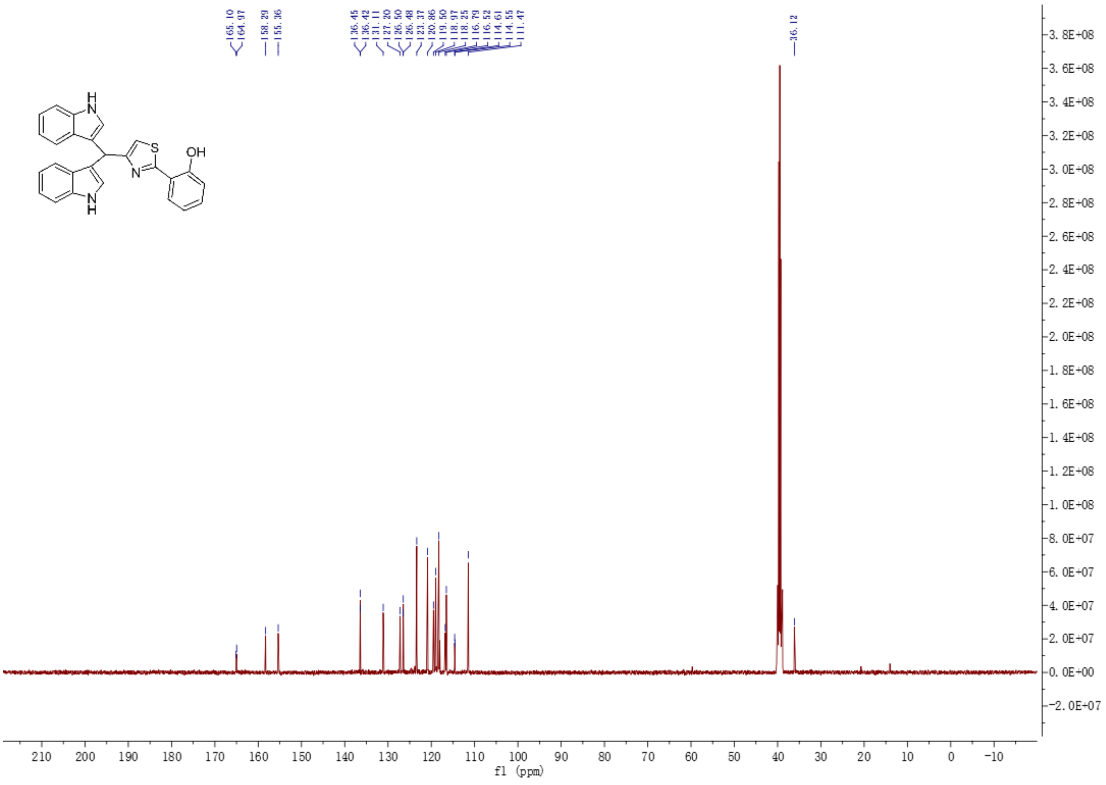

Figure S15. ^{1}H NMR spectrum (400 MHz, DMSO-d_6) of 8a

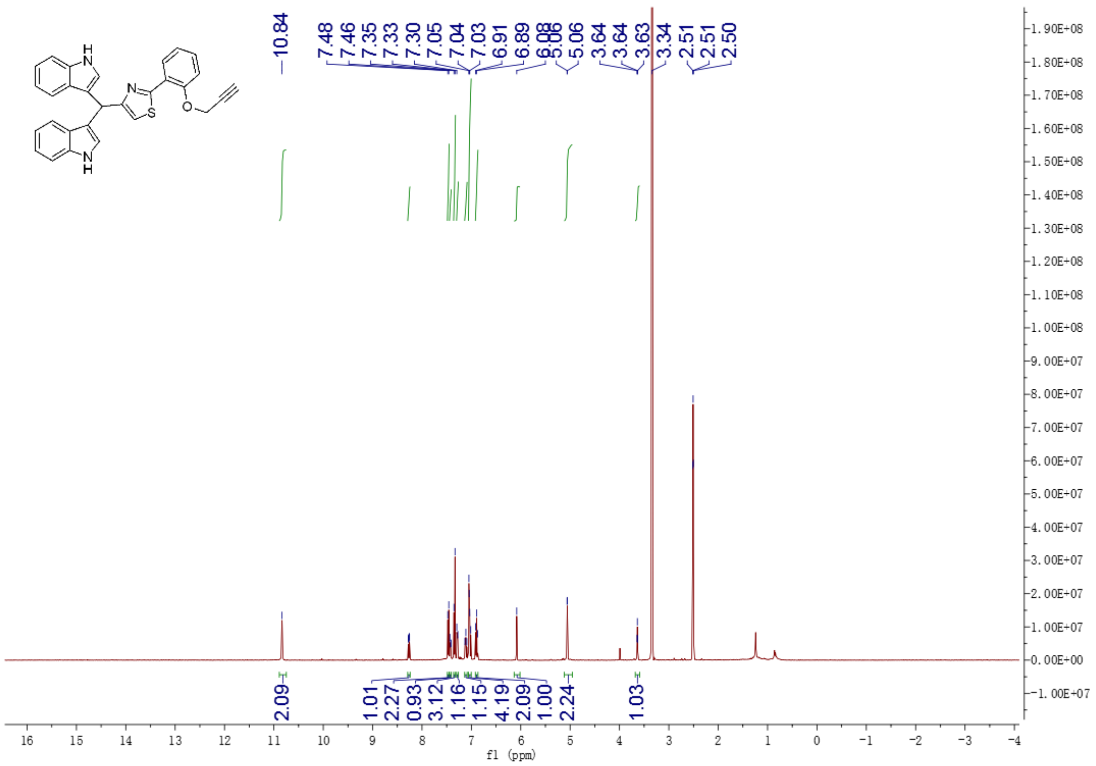

Figure S16. ^{13}C NMR spectrum (100 MHz, DMSO-d_6) of 8a

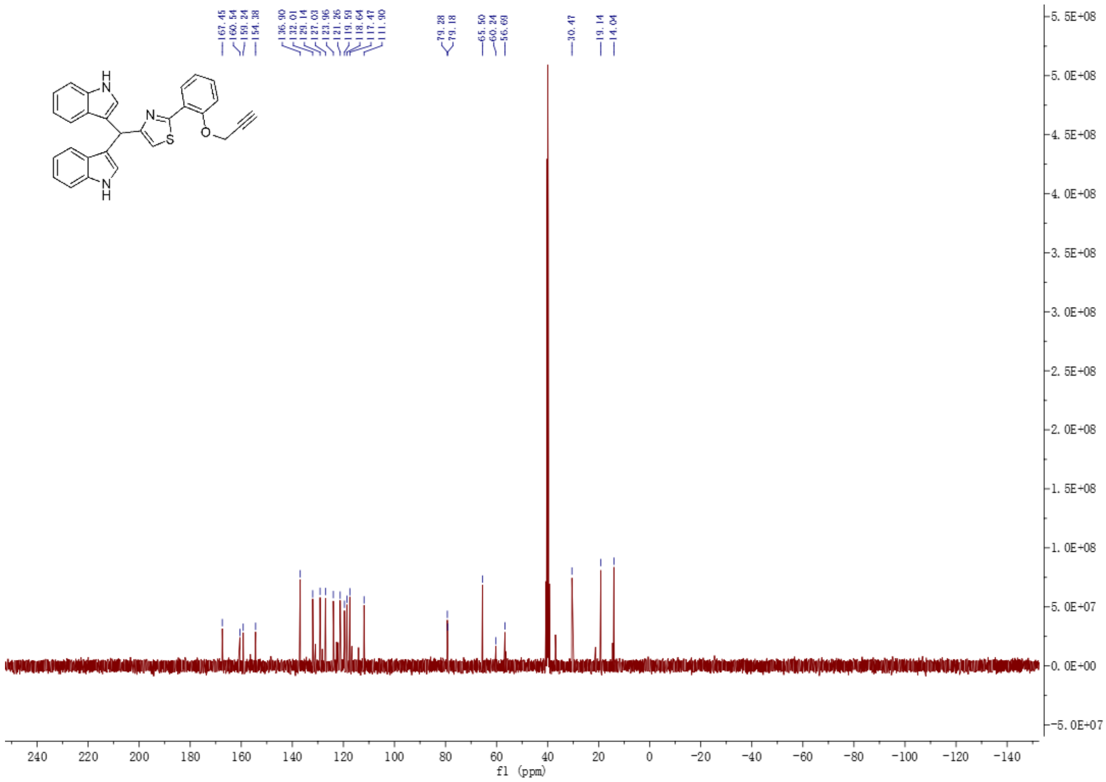

Figure S17. ^{1}H NMR spectrum (400 MHz, DMSO-d_6) of 8b

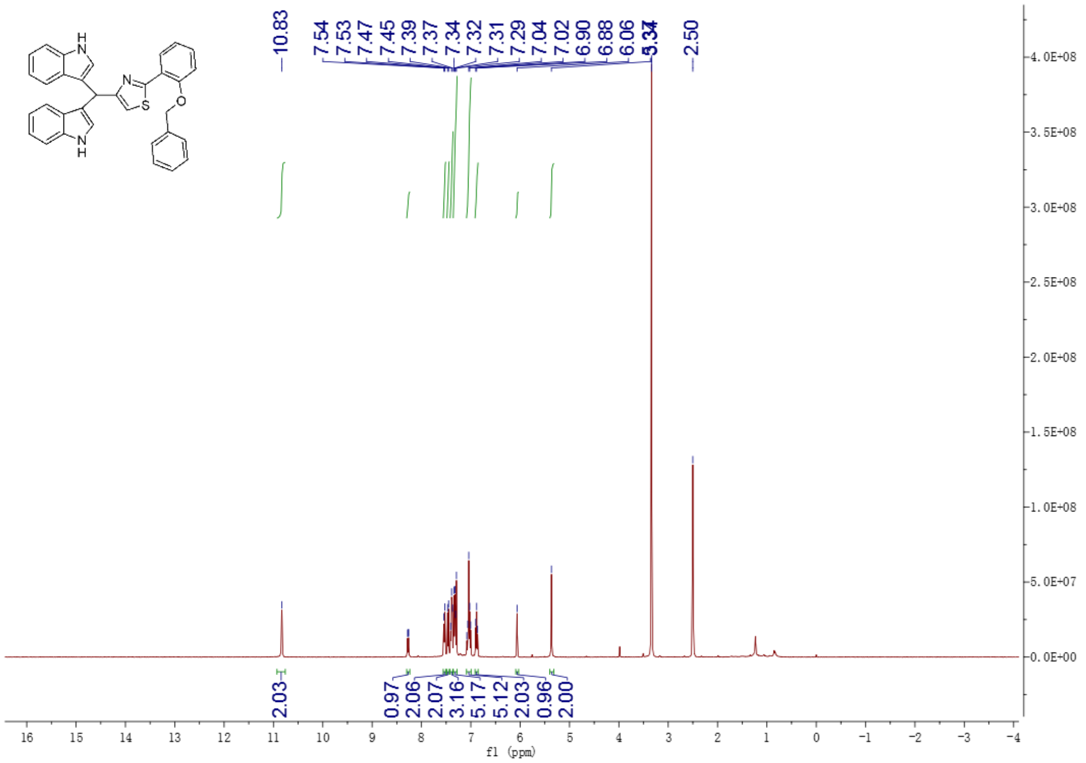

Figure S18. ^{13}C NMR spectrum (100 MHz, DMSO-d_6) of 8b

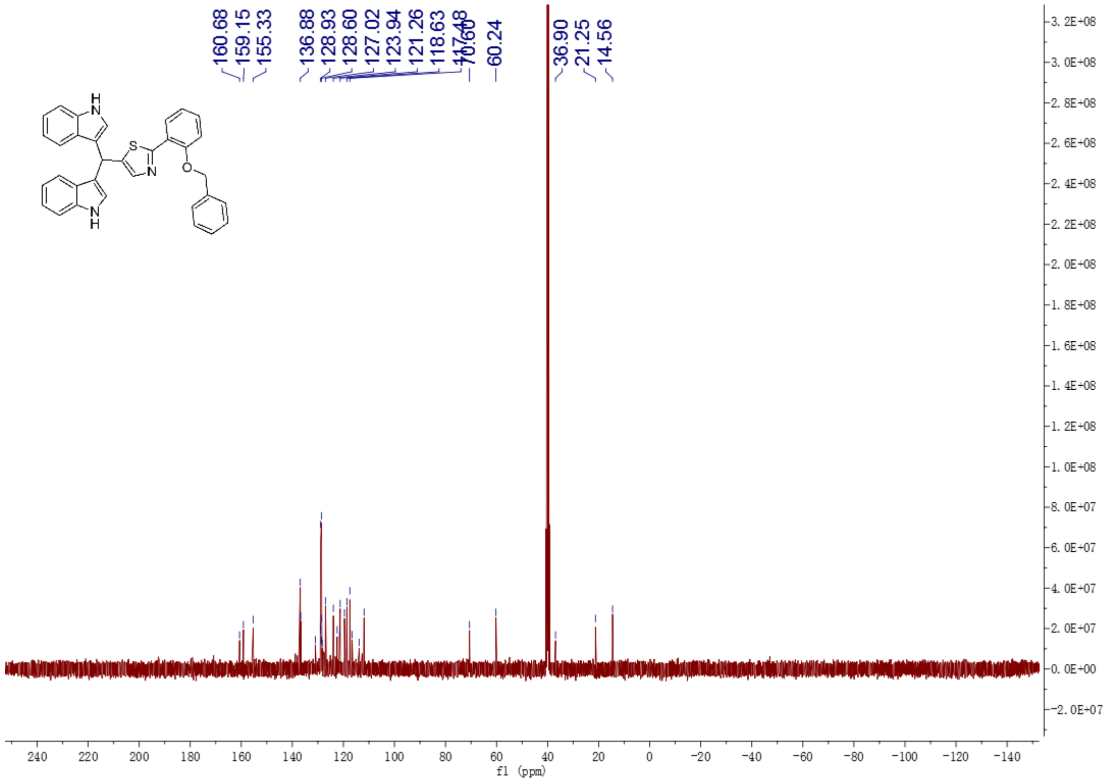

Figure S19. ^{1}H NMR spectrum (400 MHz, DMSO-d_6) of 9a

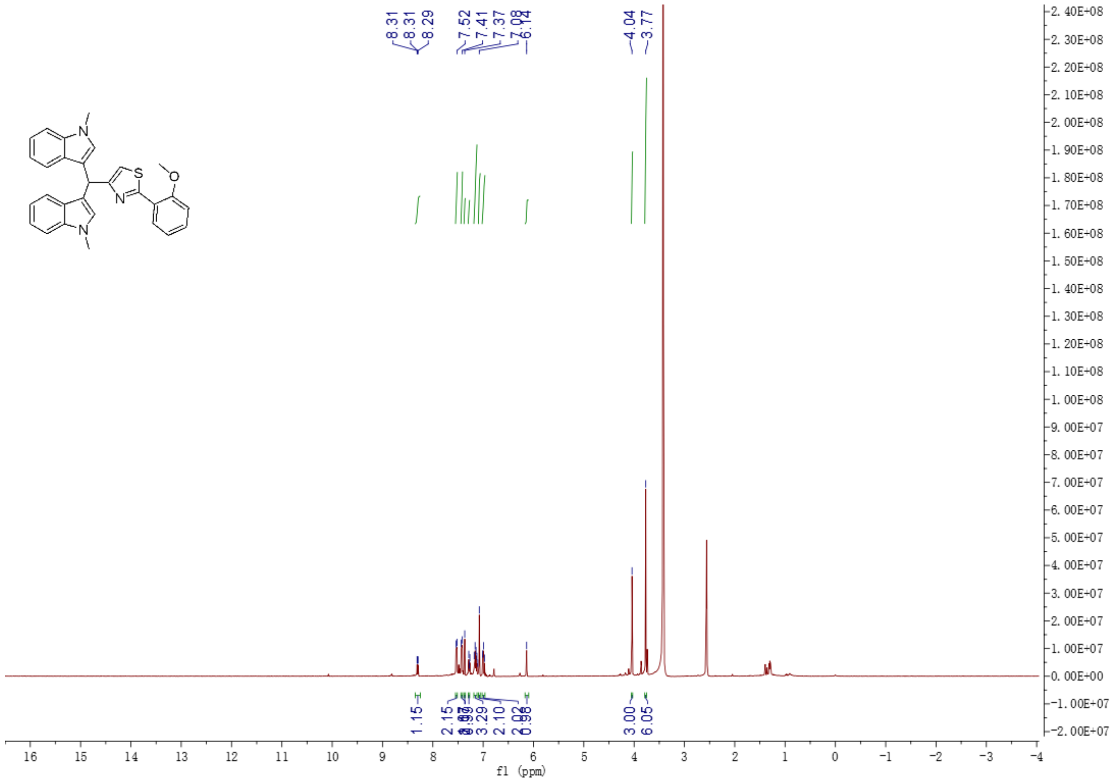

Figure S20. ^{13}C NMR spectrum (100 MHz, DMSO-d_6) of 9a

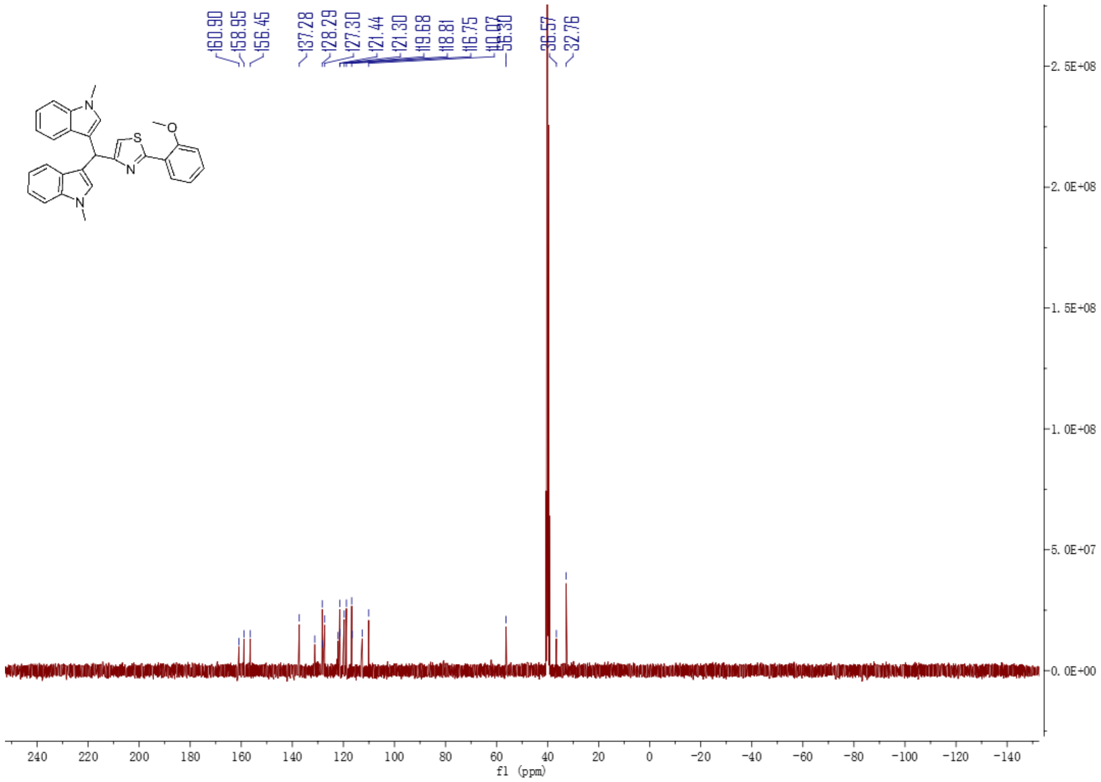

Figure S21. ^{1}H NMR spectrum (400 MHz, DMSO-d_6) of 9b

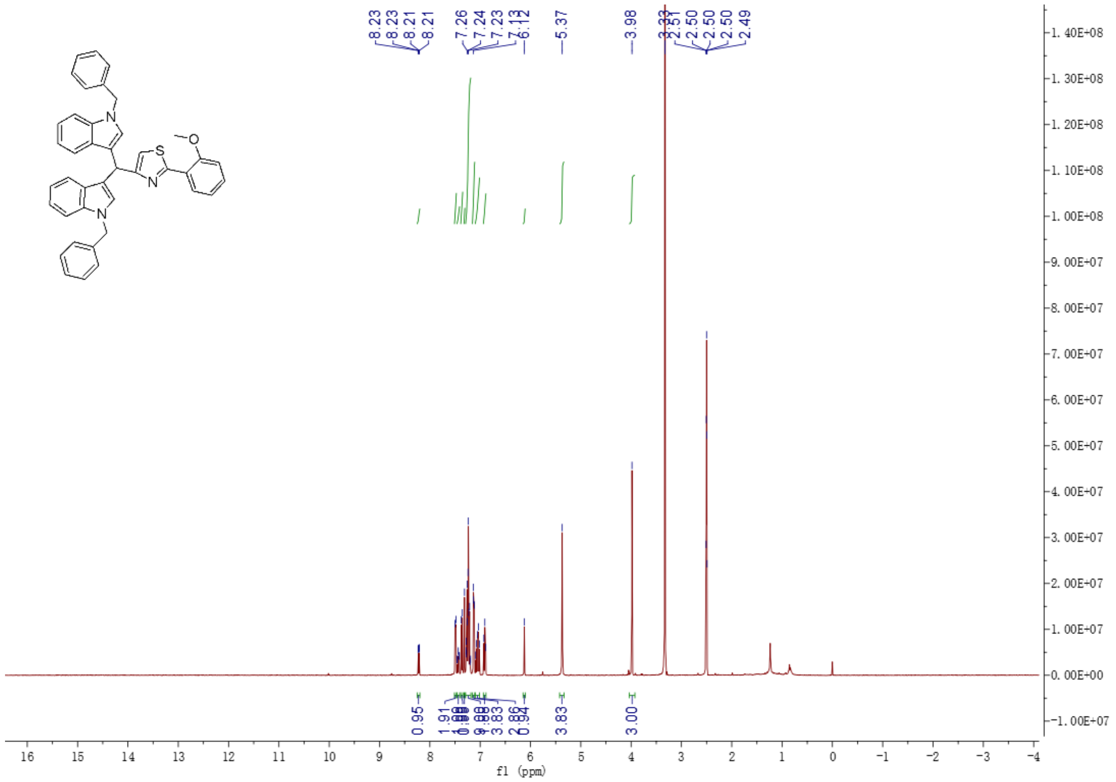

Figure S22. ^{13}C NMR spectrum (100 MHz, DMSO-d_6) of 9b

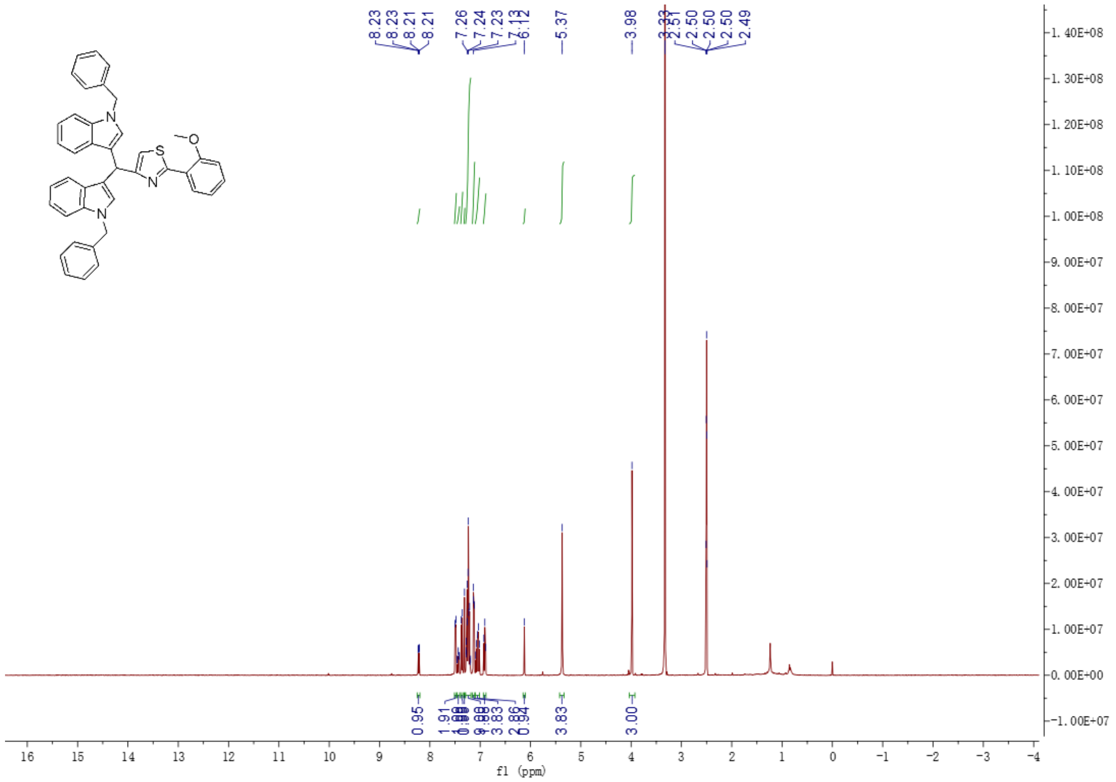

Figure S23. ^{1}H **NMR spectrum (400 MHz, DMSO-*d*$_6$) of 9c**

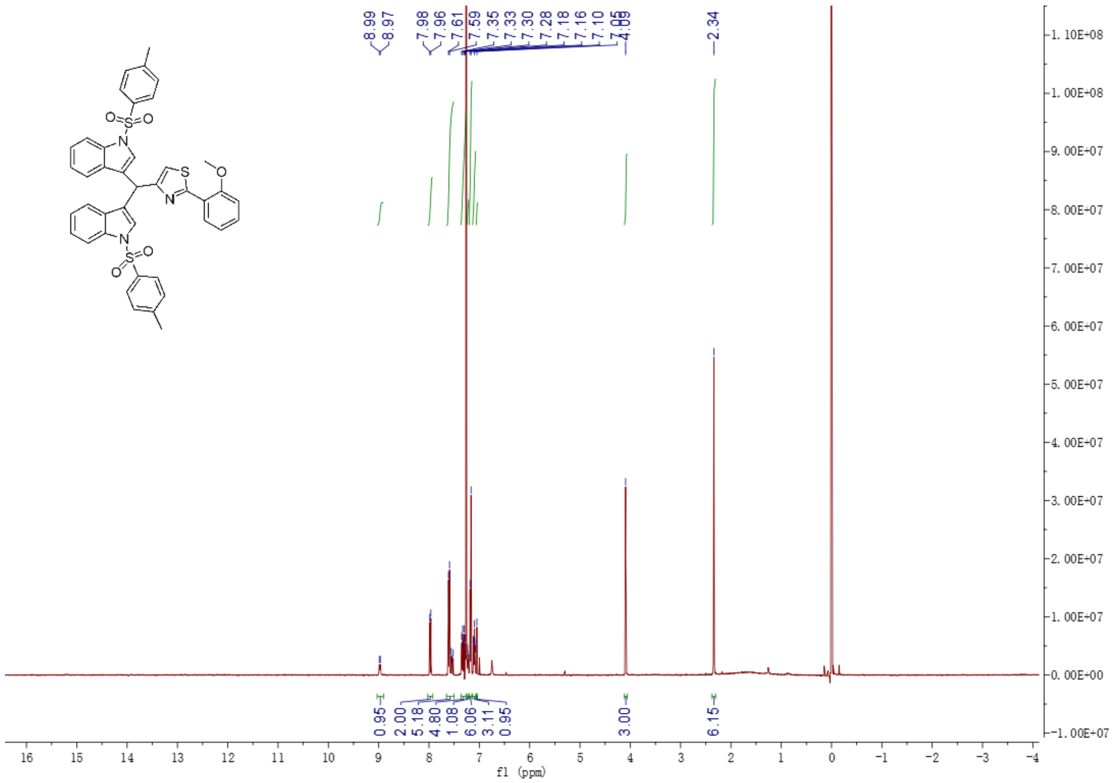

Figure S24. ^{13}C **NMR spectrum (100 MHz, DMSO-*d*$_6$) of 9c**

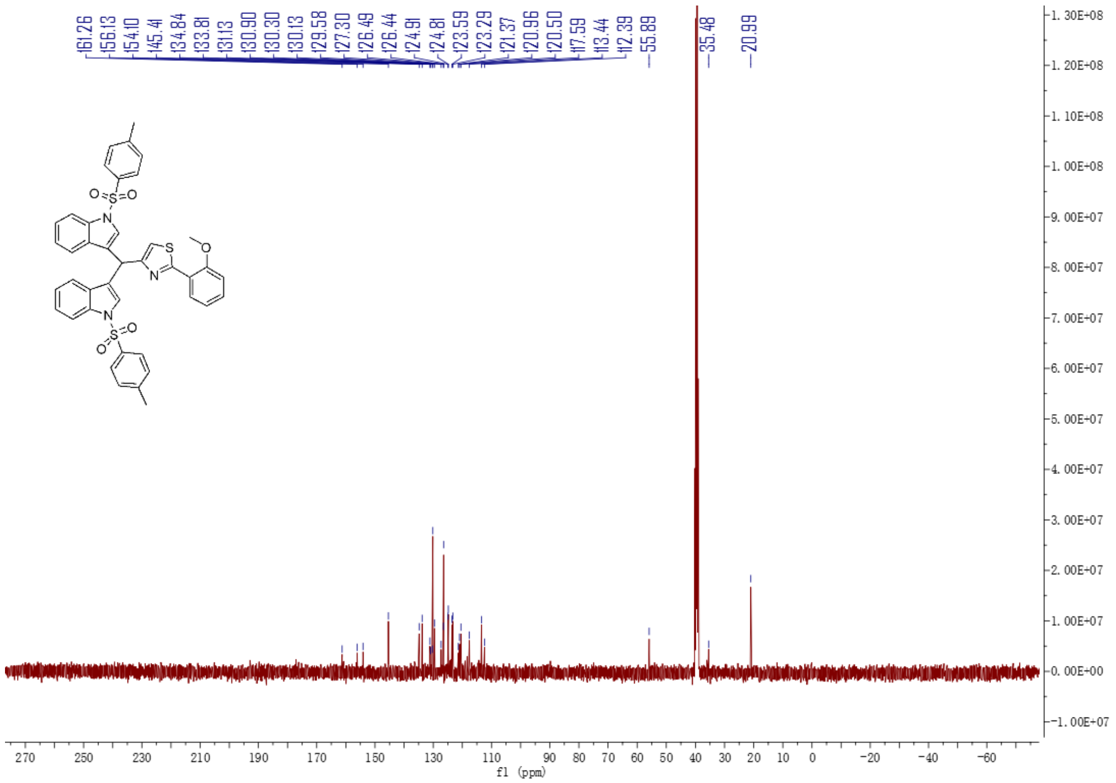

Figure S25. ^{1}H NMR spectrum (400 MHz, DMSO-d_6) of 9d

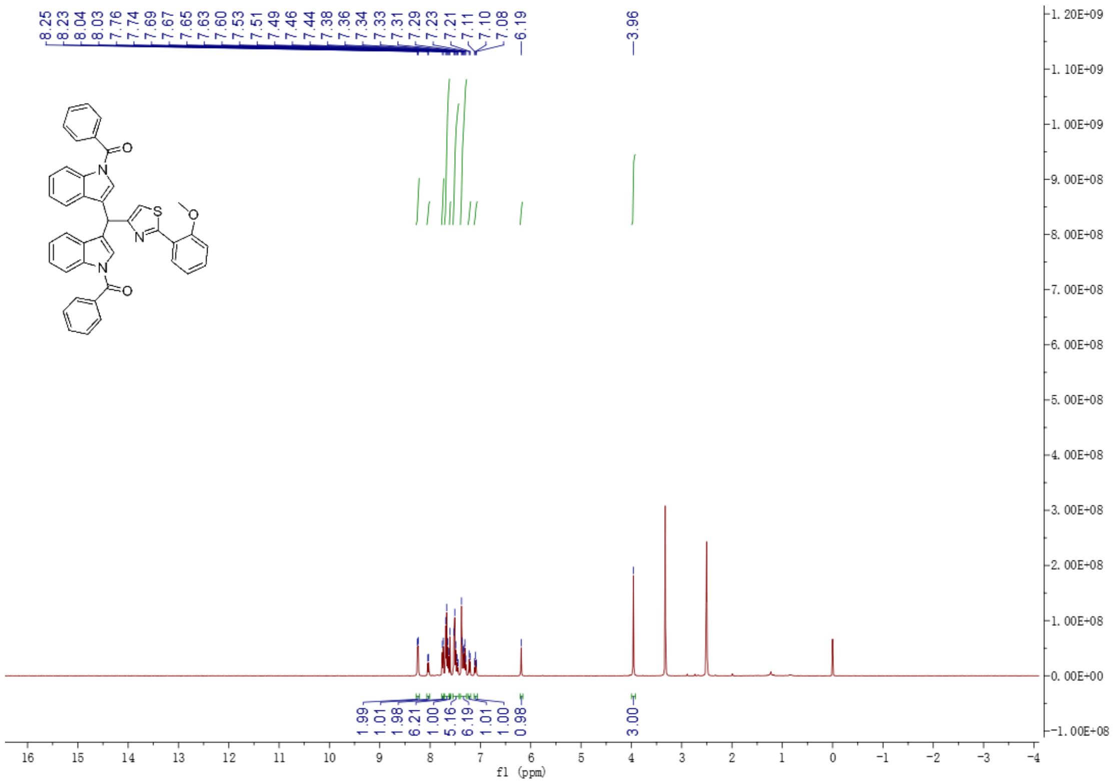

Figure S26. ^{13}C NMR spectrum (100 MHz, DMSO-d_6) of 9d

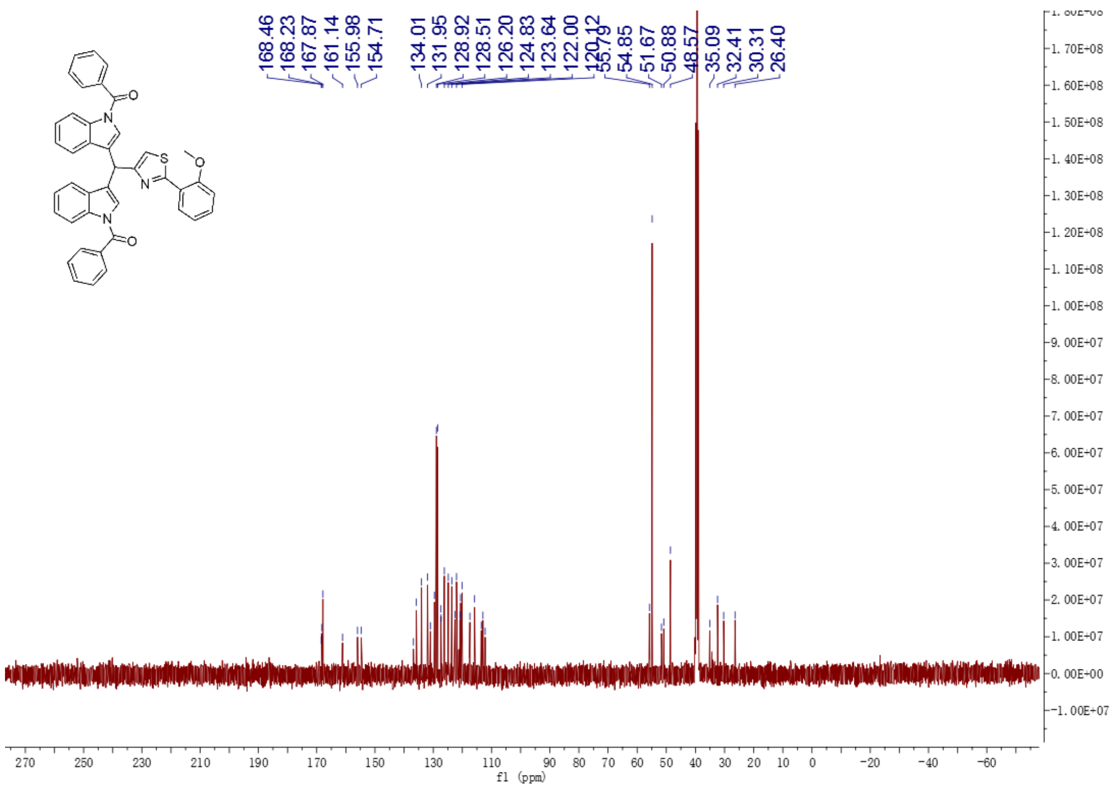

Figure S27. ^{1}H NMR spectrum (400 MHz, DMSO-d_6) of 9e

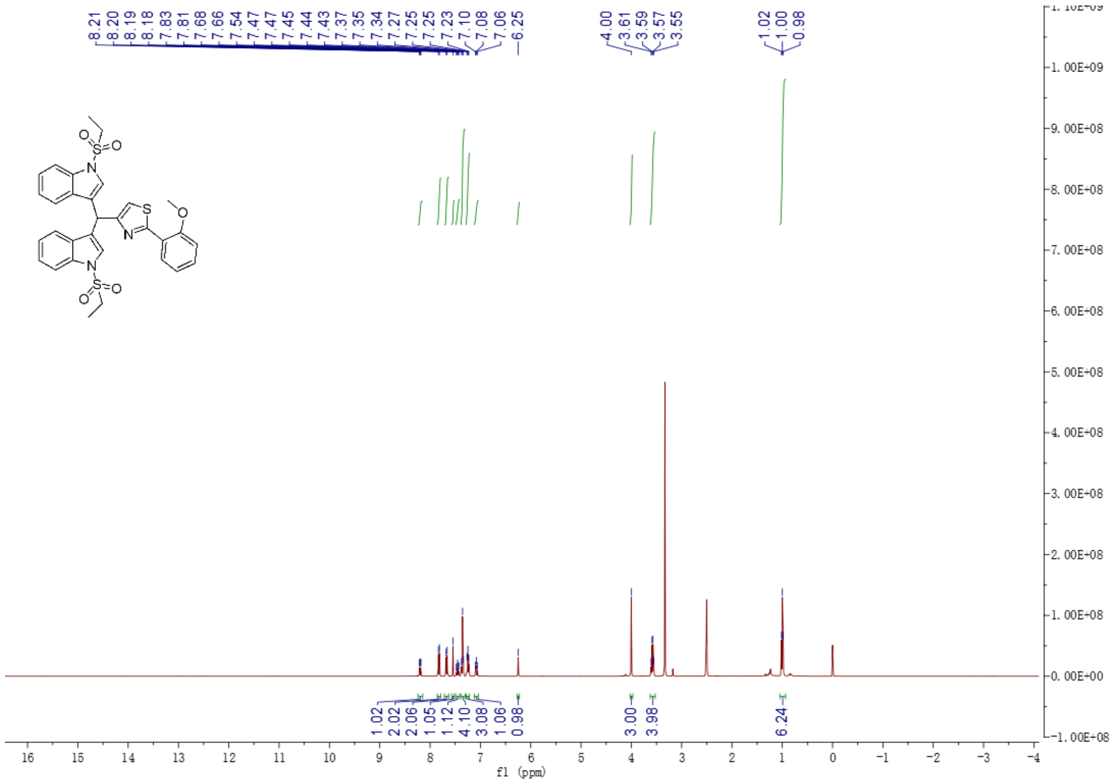

Figure S28. ^{13}C NMR spectrum (100 MHz, DMSO-d_6) of 9e

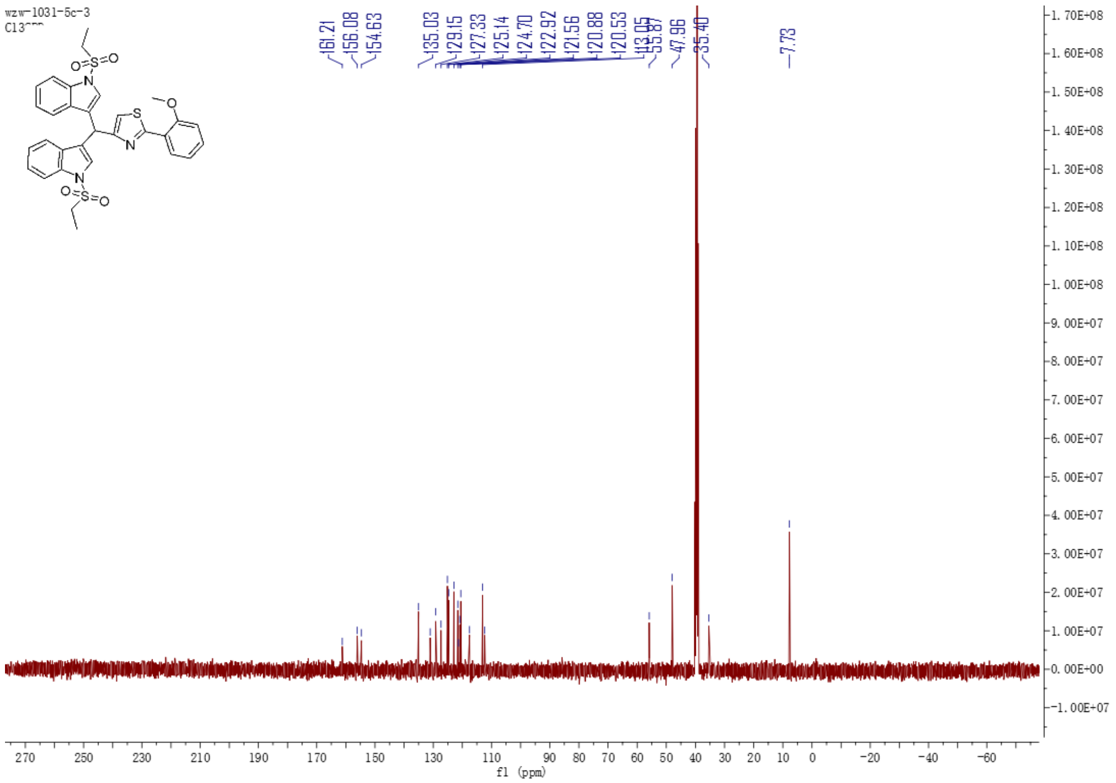

Figure S29. ^{1}H NMR spectrum (400 MHz, $CDCl_3$) of 10

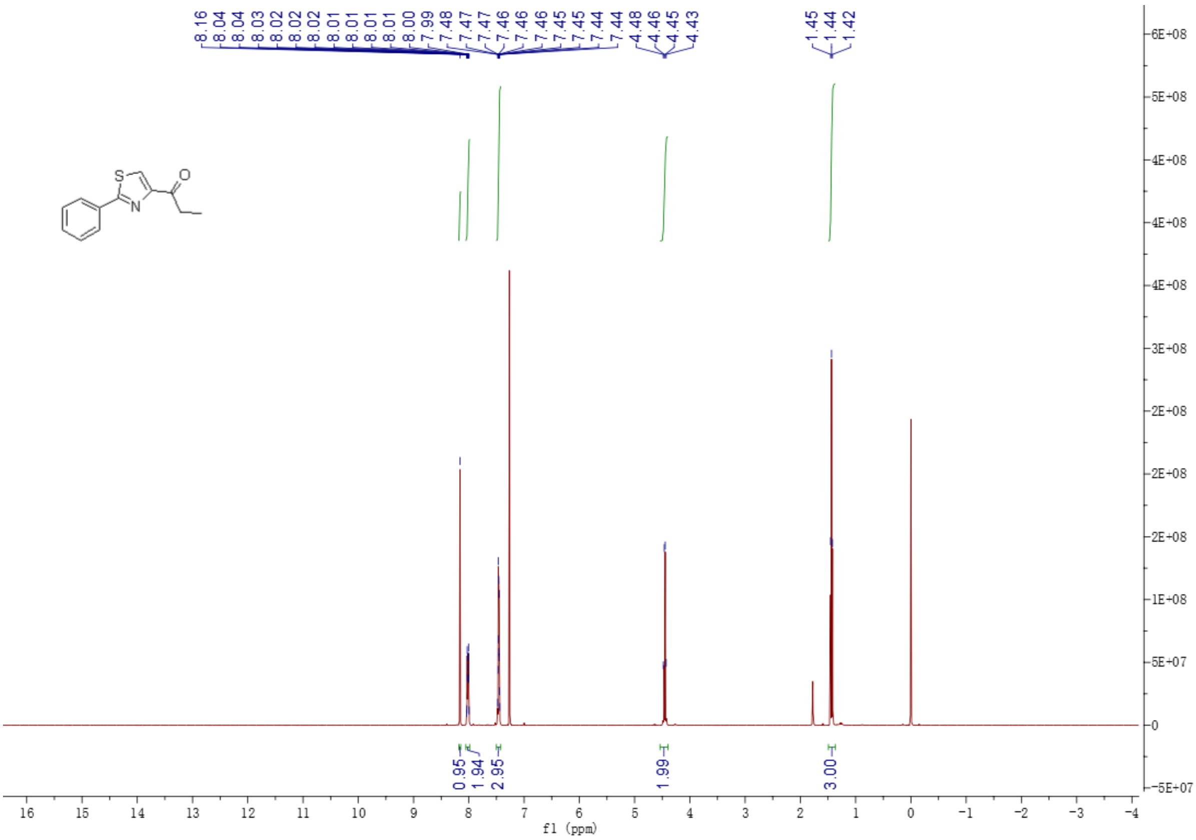

Figure S30. ^{13}C NMR spectrum (100 MHz, $CDCl_3$) of 10

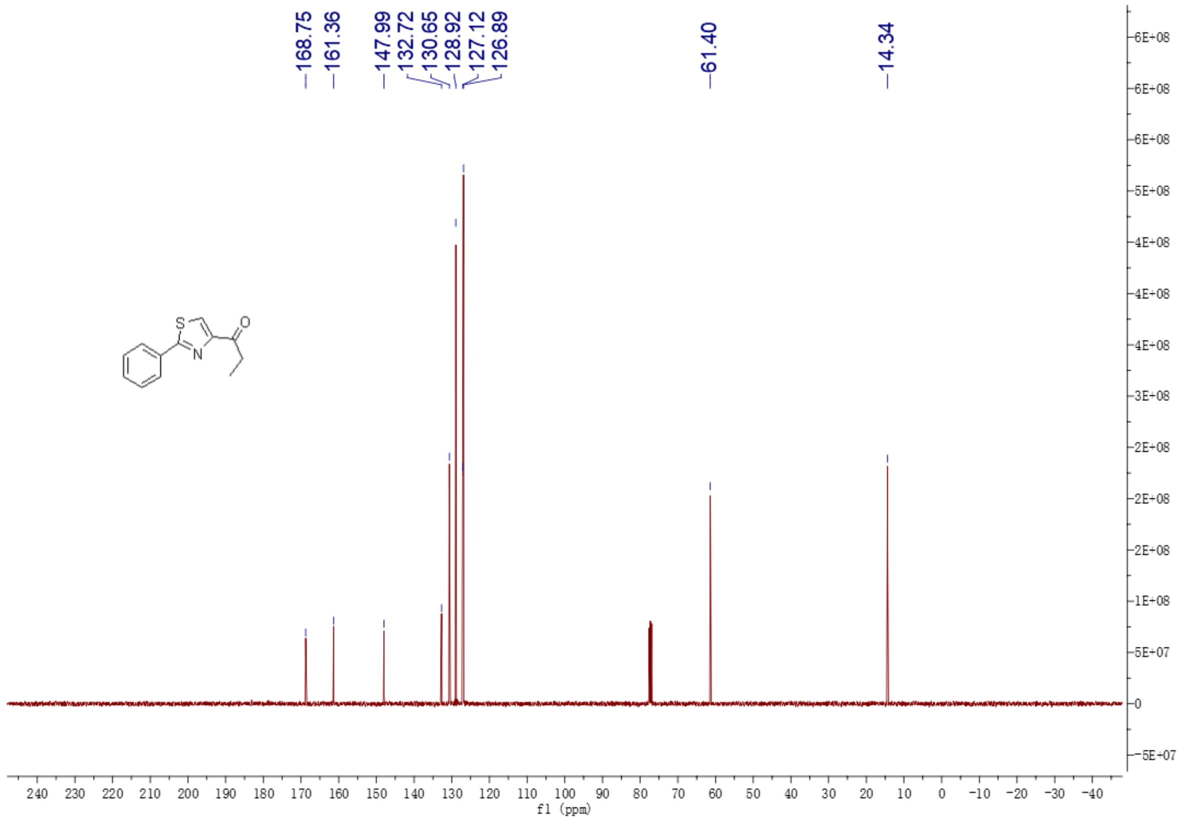

Figure S31. ^{1}H NMR spectrum (400 MHz, DMSO-d_6) of 11

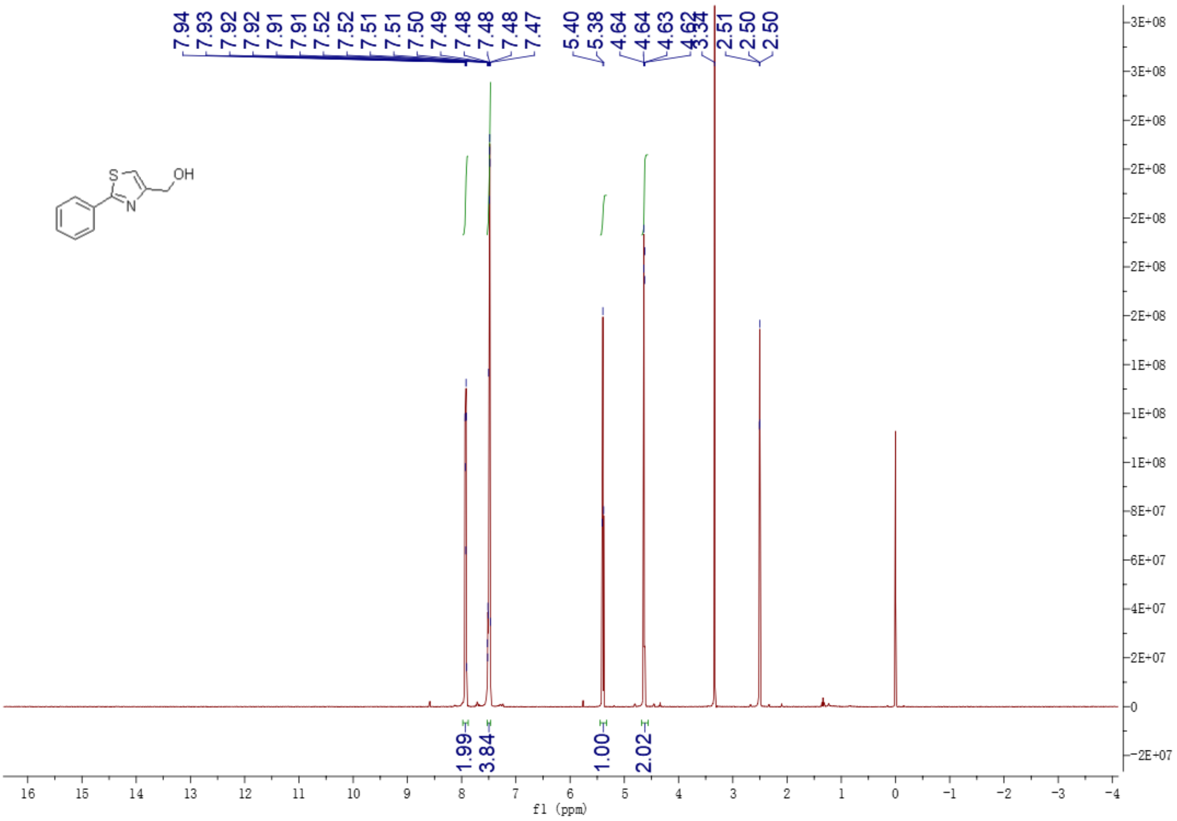

Figure S32. ^{13}C NMR spectrum (100 MHz, DMSO-d_6) of 11

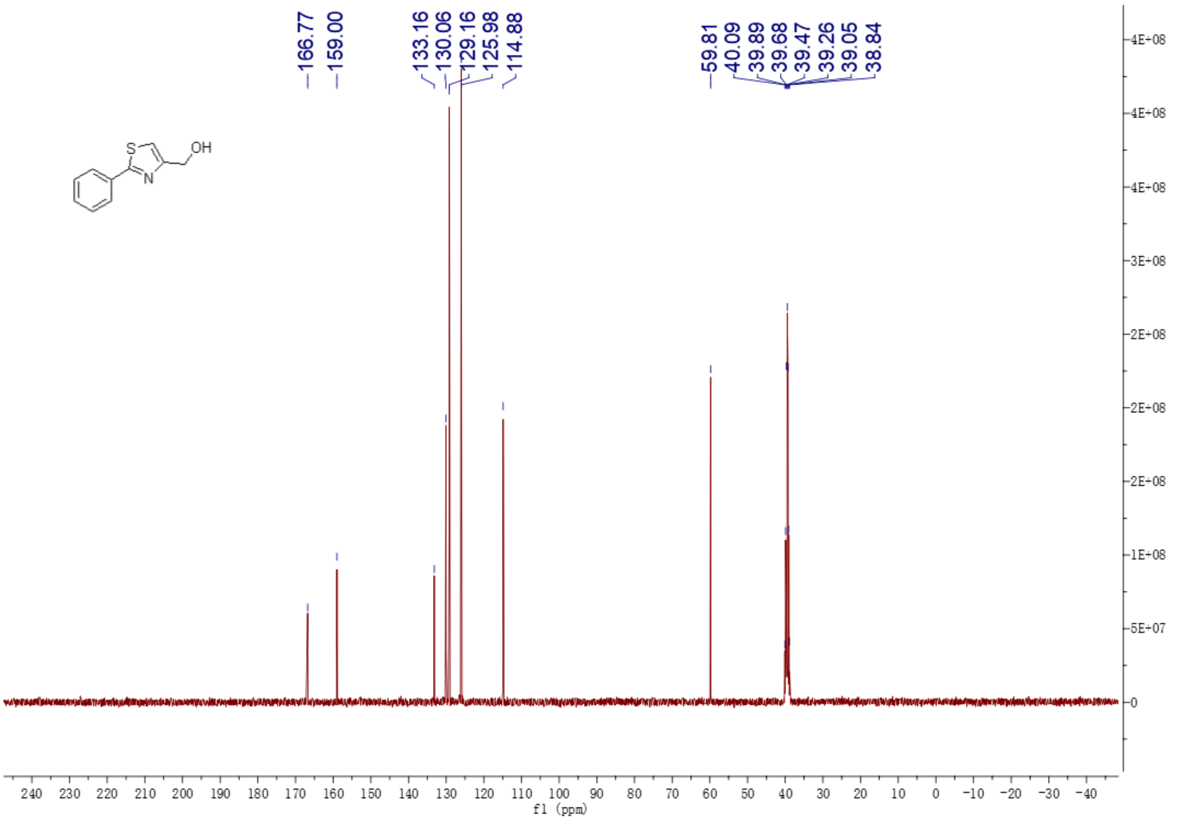

Figure S33. ^{1}H NMR spectrum (400 MHz, $CDCl_3$) of 12

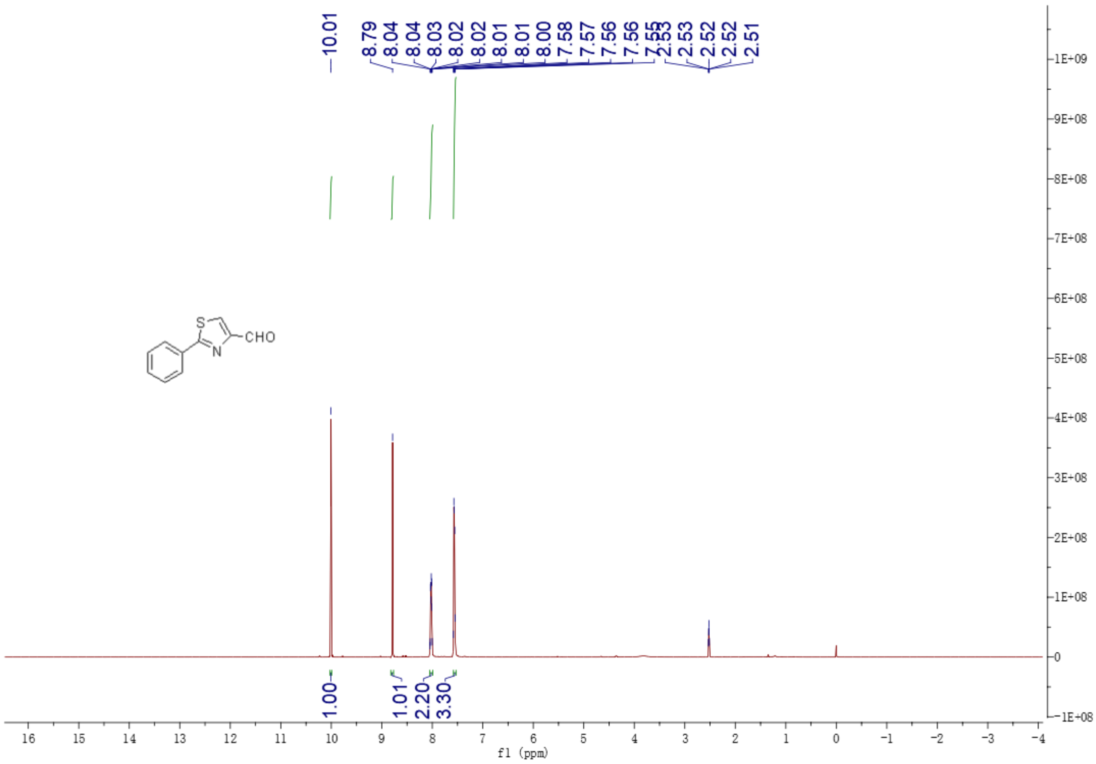

Figure S34. ^{13}C NMR spectrum (100 MHz, $CDCl_3$) of 12

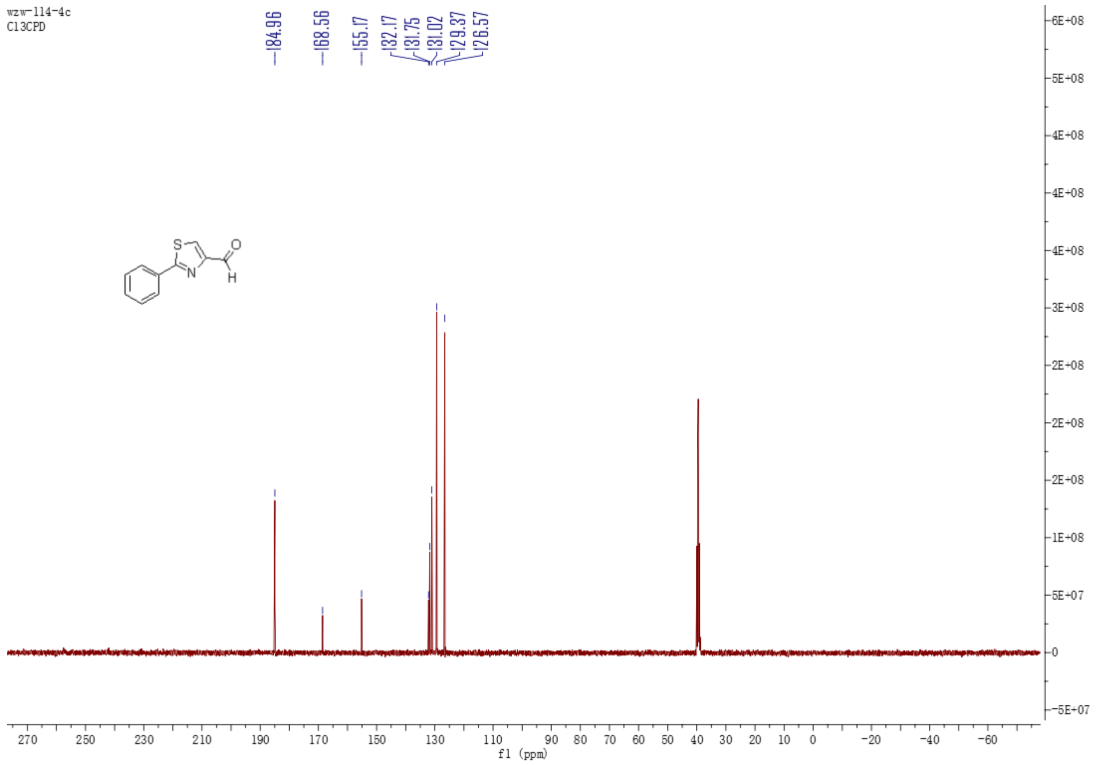

Figure S35. ^{1}H NMR spectrum (400 MHz, DMSO-d_6) of 13a

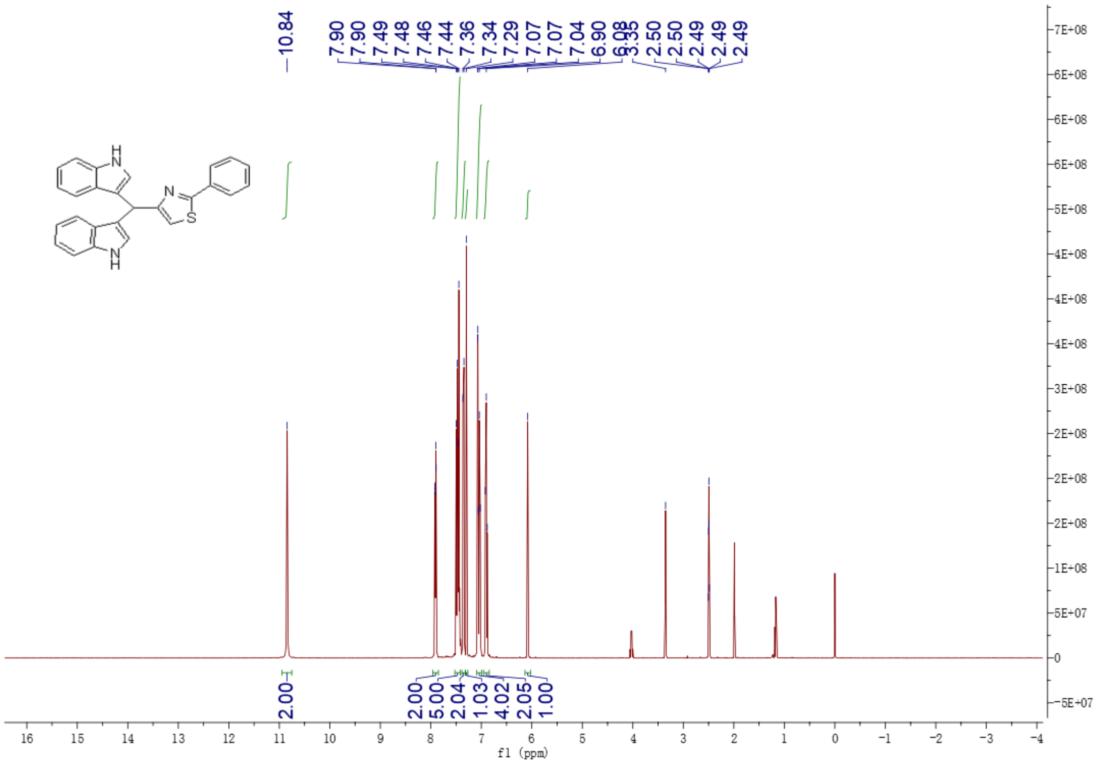

Figure S36. ^{13}C NMR spectrum (100 MHz, DMSO-d_6) of 13a

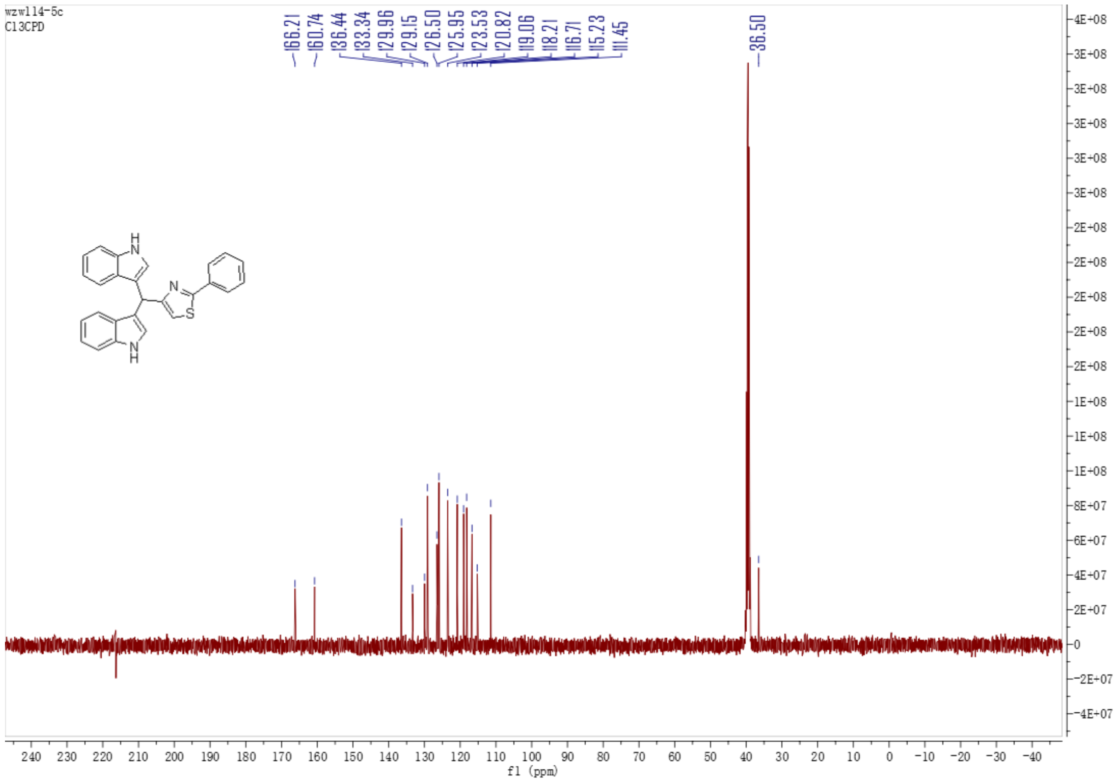

Figure S37. ^{1}H NMR spectrum (400 MHz, DMSO-d_6) of 13b

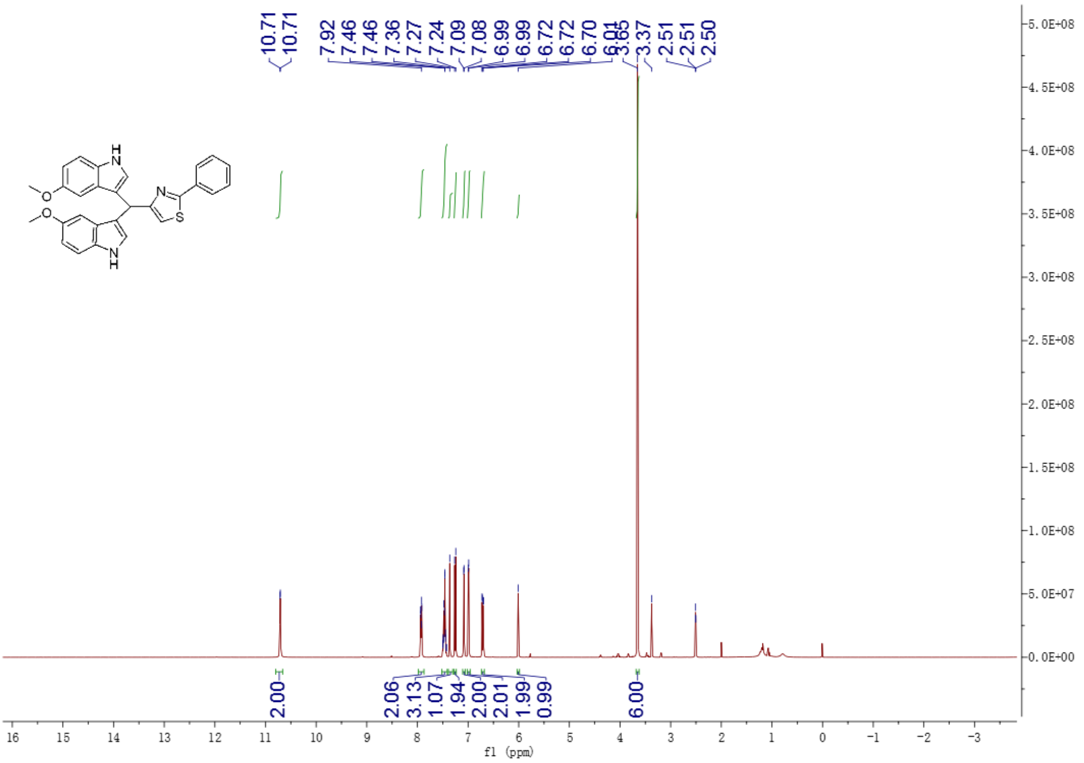

Figure S38. ^{13}C NMR spectrum (100 MHz, DMSO-d_6) of 13b

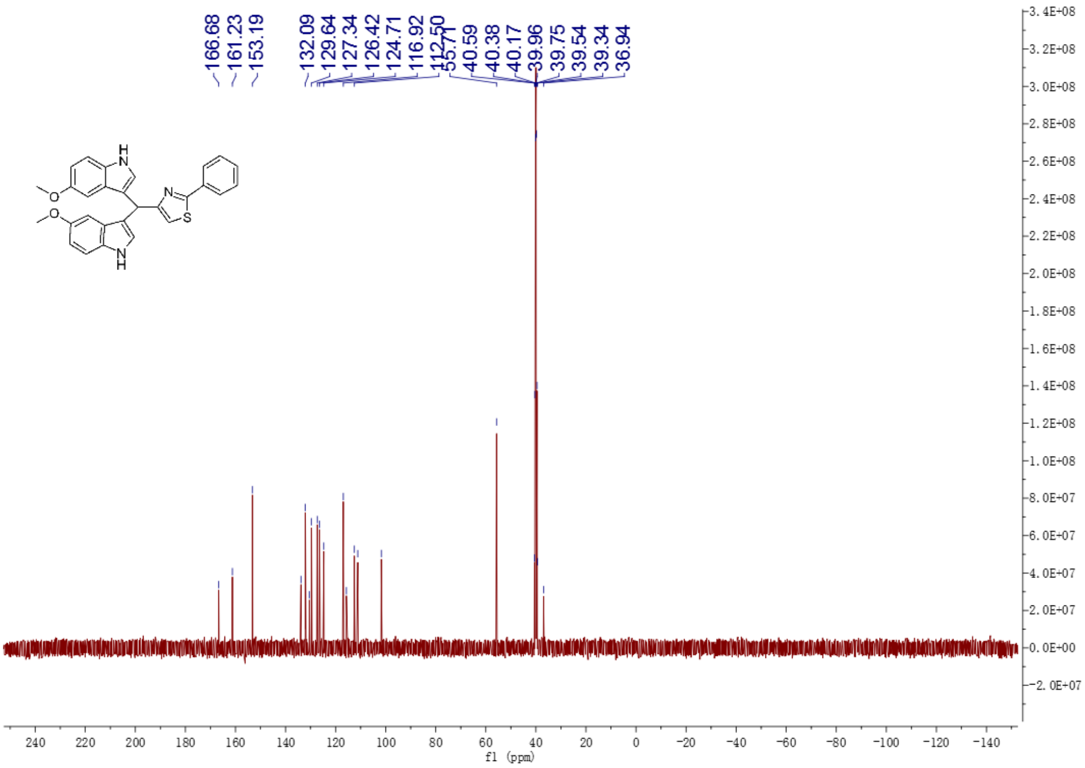

Figure S39. ^{1}H NMR spectrum (400 MHz, DMSO-d_6) of 13c

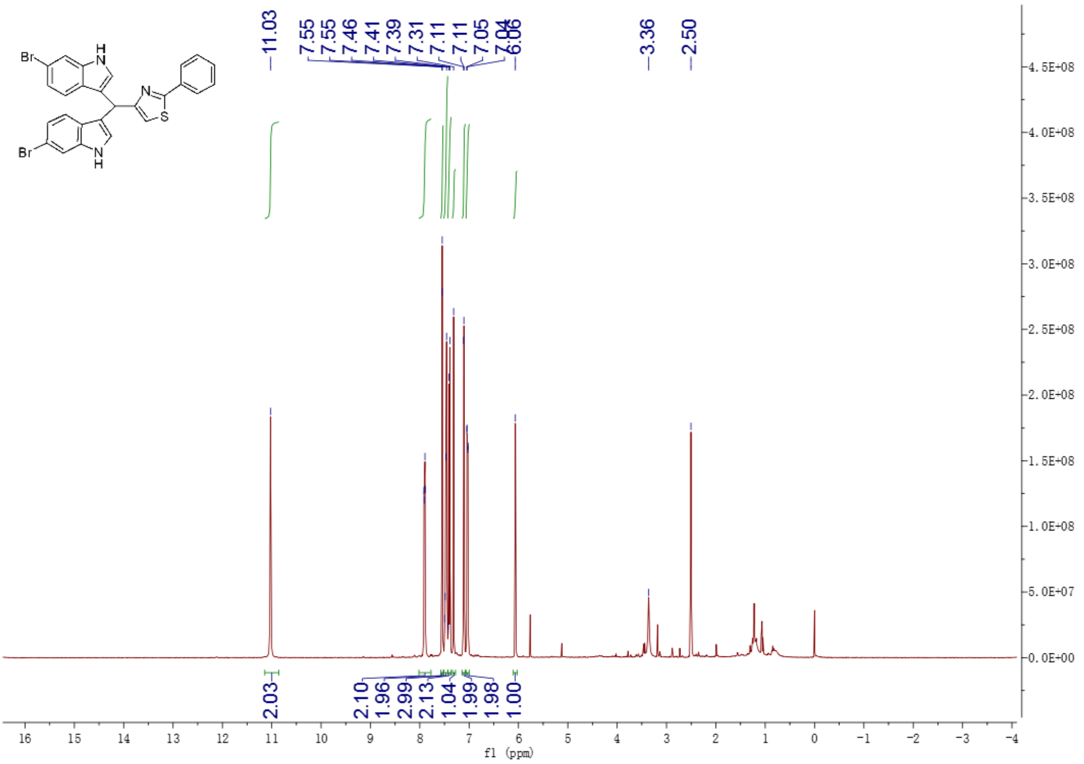

Figure S40. ^{13}C NMR spectrum (100 MHz, DMSO-d_6) of 13c

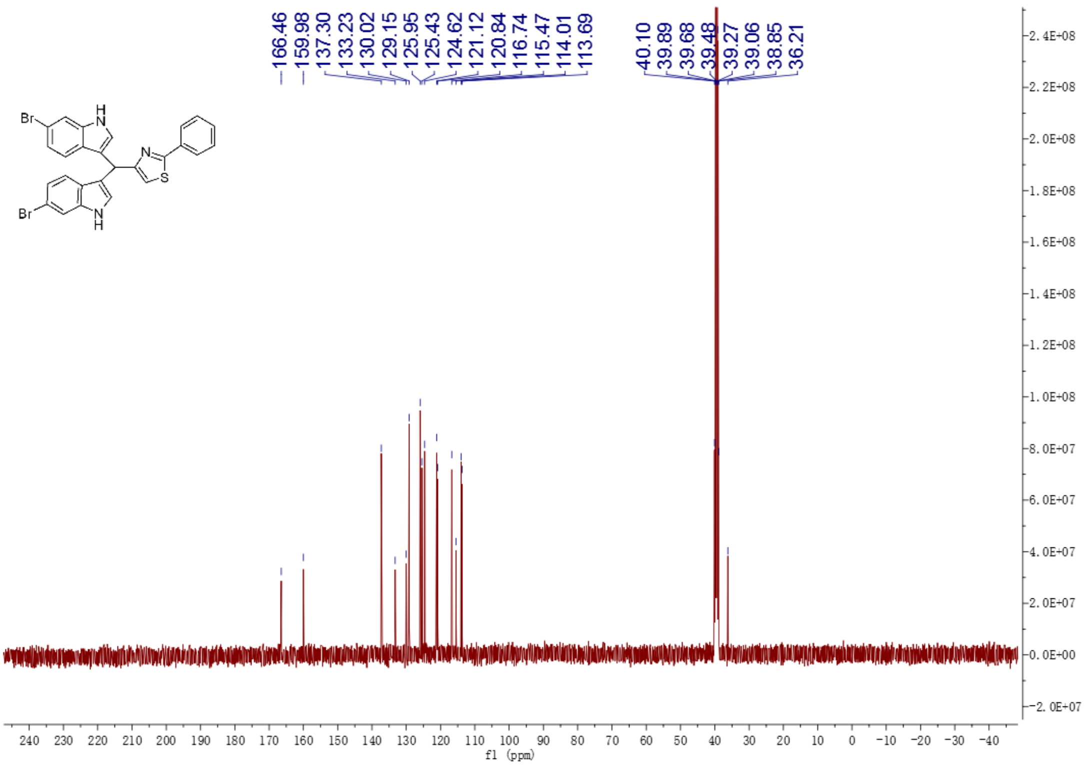

Figure S41. ^{1}H NMR spectrum (400 MHz, DMSO-d_6) of 13d

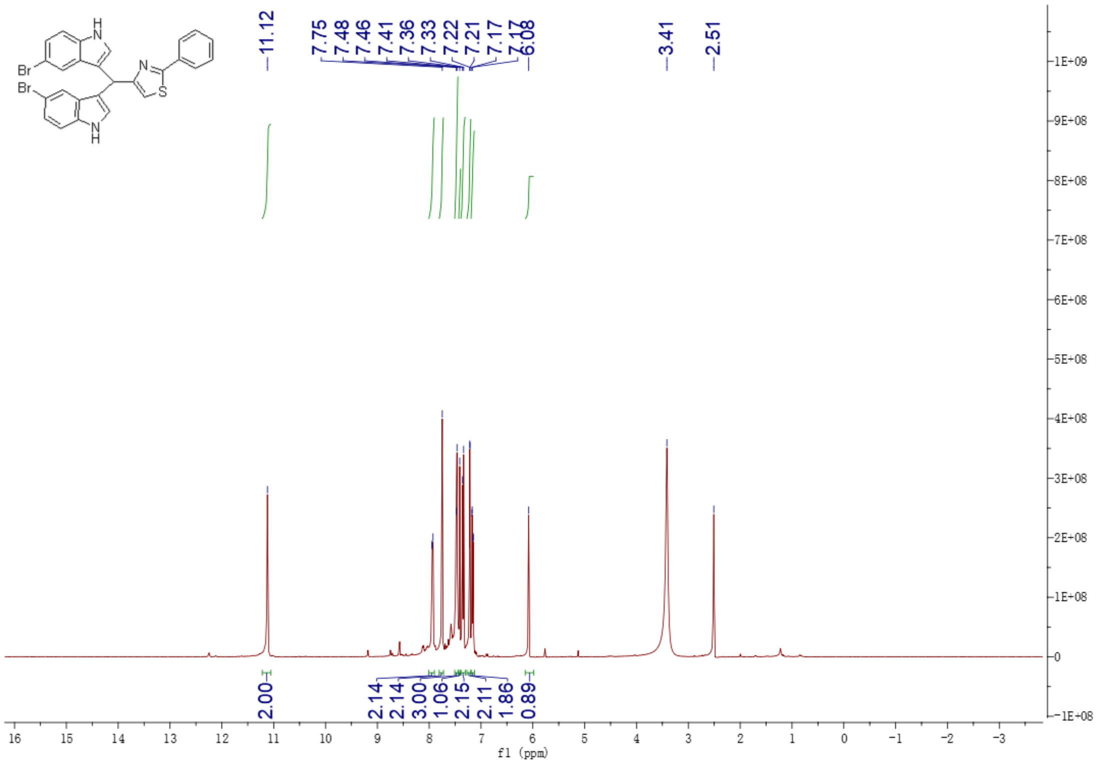

Figure S42. ^{13}C NMR spectrum (100 MHz, DMSO-d_6) of 13d

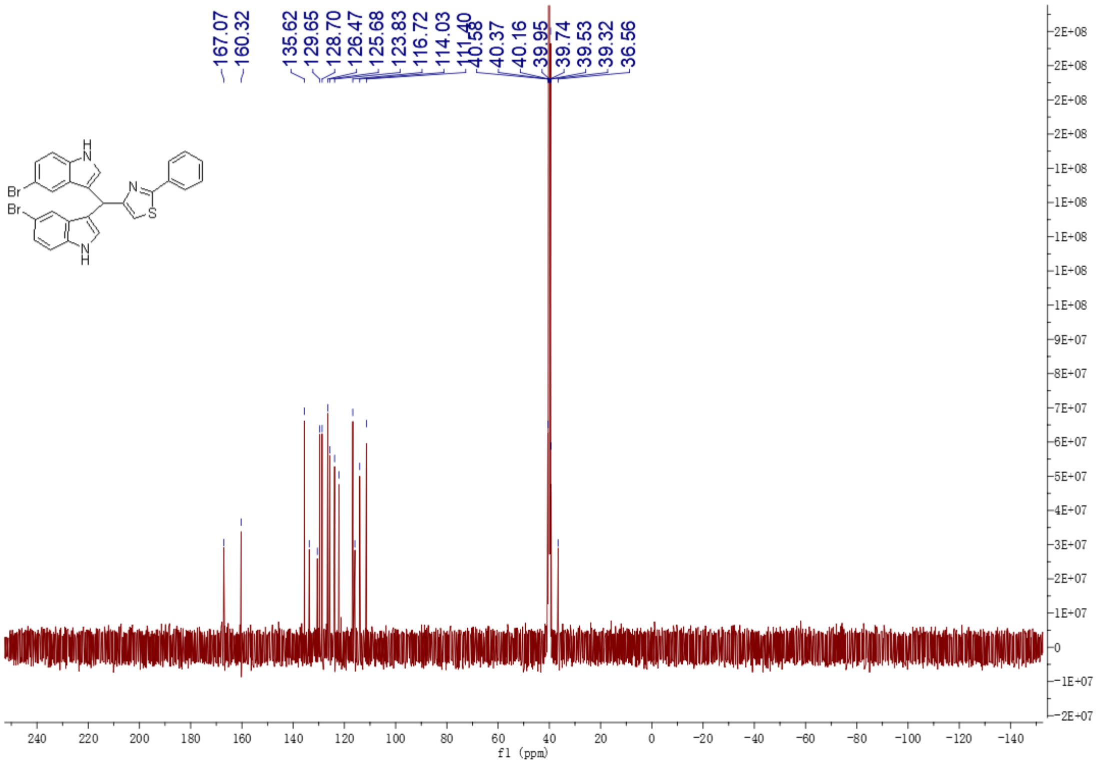

Figure S43. ^{1}H NMR spectrum (400 MHz, DMSO-d_6) of 14a

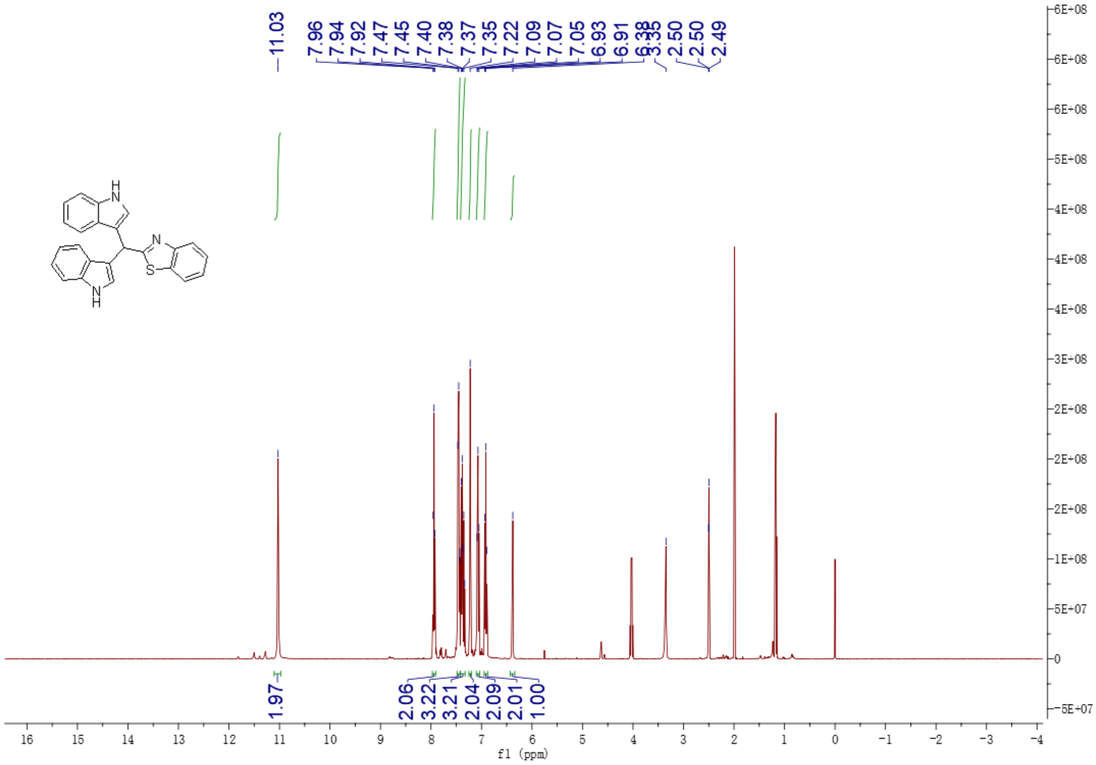

Figure S44. ^{13}C NMR spectrum (100 MHz, DMSO-d_6) of 14a

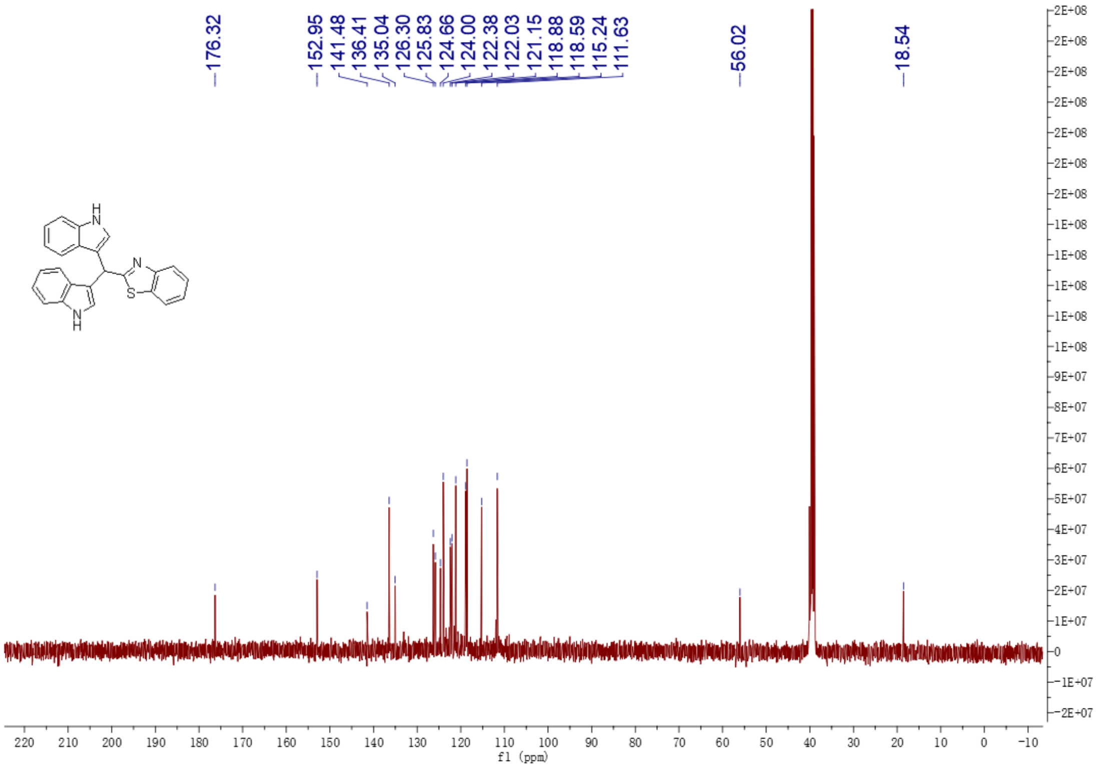

Figure S45. ^{1}H NMR spectrum (400 MHz, DMSO-d_6) of 14b

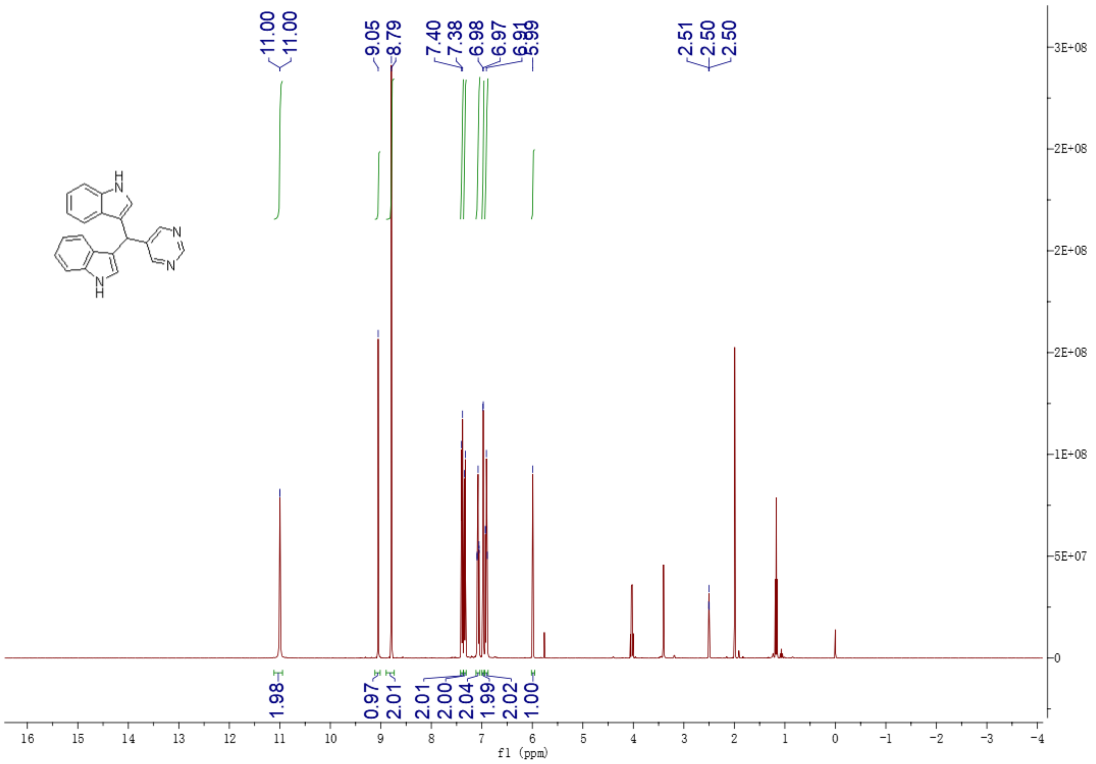

Figure S46. ^{13}C NMR spectrum (100 MHz, DMSO-d_6) of 14b

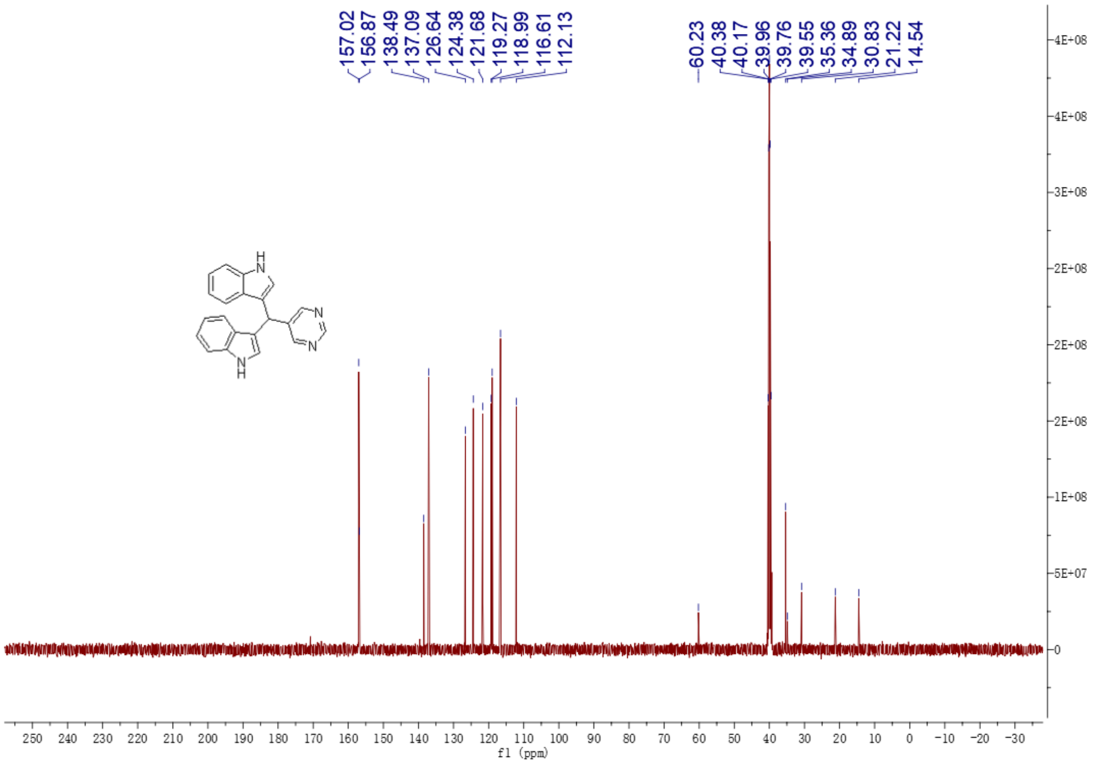

Figure S47. ^{1}H NMR spectrum (400 MHz, DMSO-d_6) of 14c

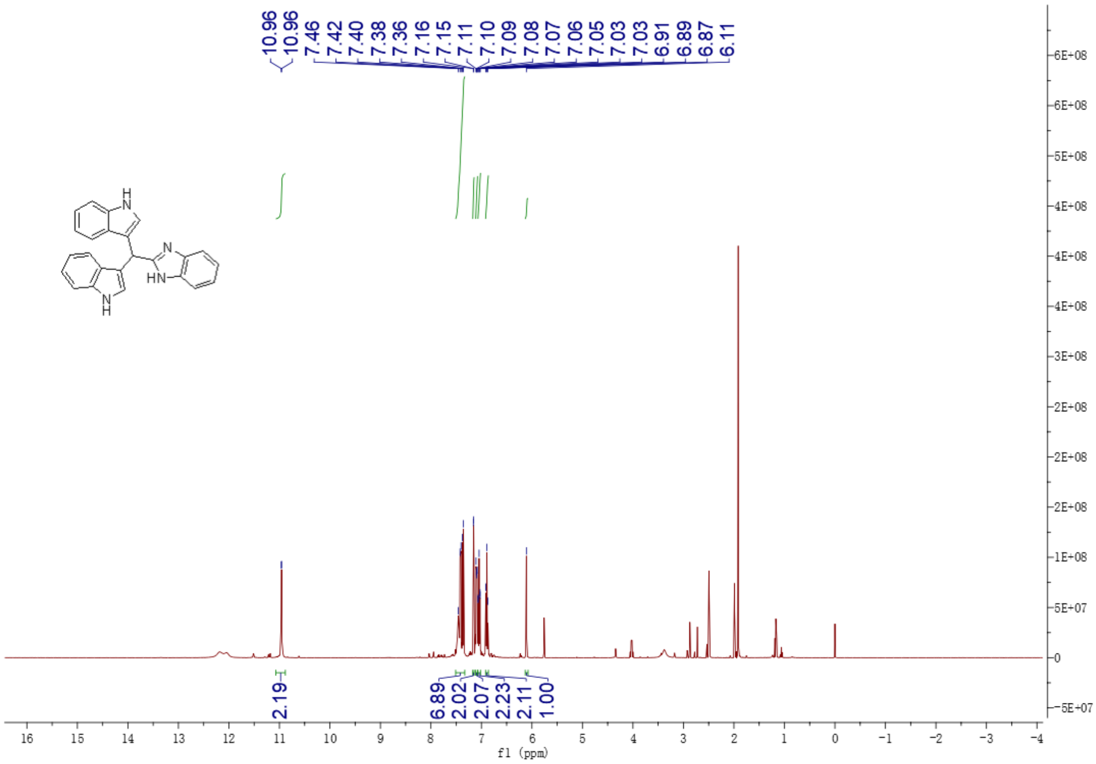

Figure S48. ^{13}C NMR spectrum (100 MHz, DMSO-d_6) of 14c

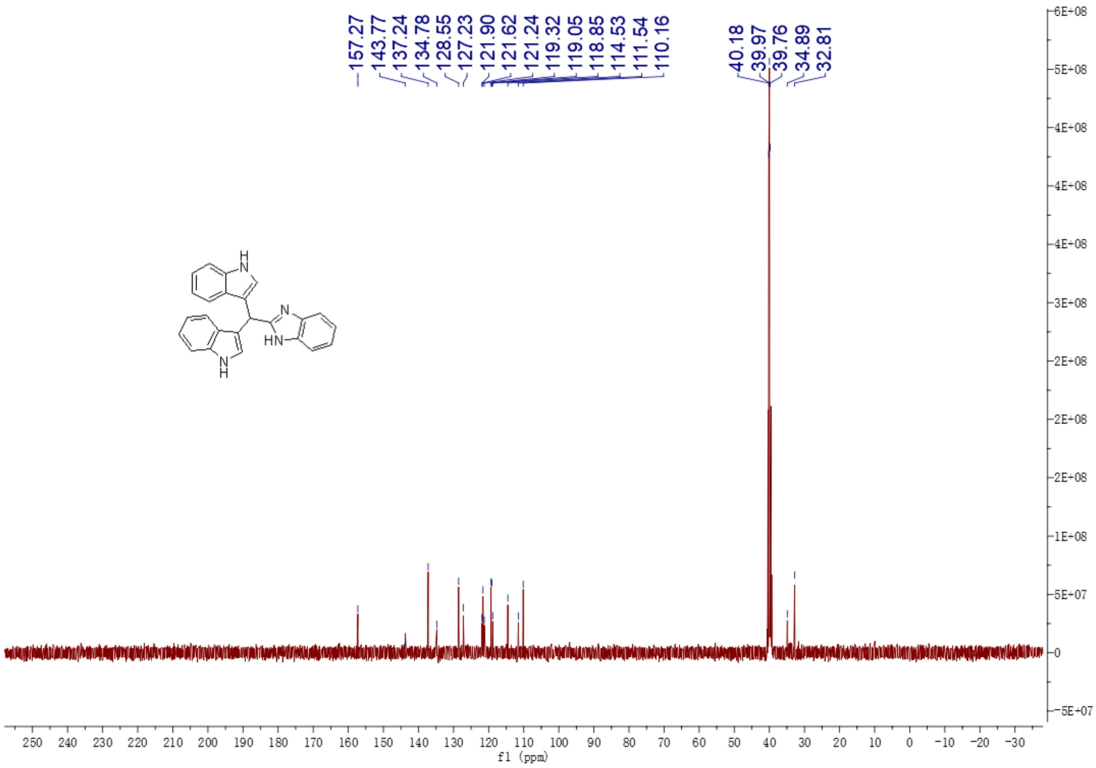

Figure S49. ^{1}H NMR spectrum (400 MHz, DMSO-d_6) of 14d

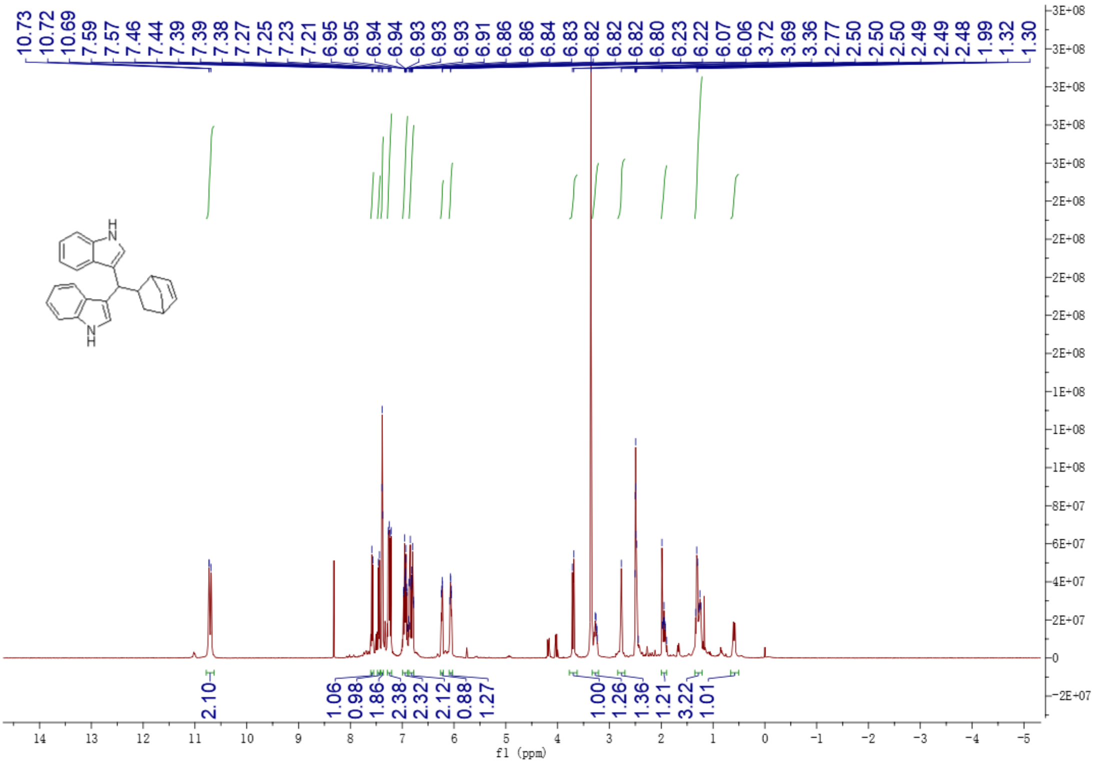

Figure S50. ^{13}C NMR spectrum (100 MHz, DMSO-d_6) of 14d

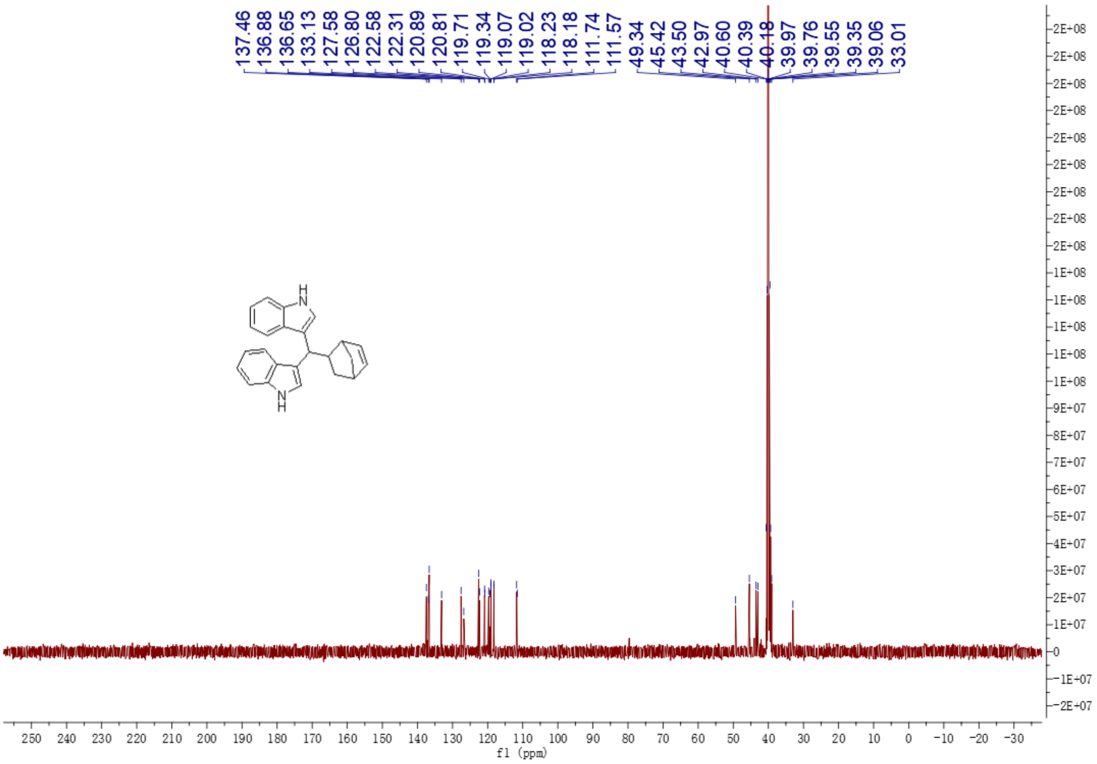

Figure S51. ^{1}H NMR spectrum (400 MHz, DMSO-d_6) of 14e

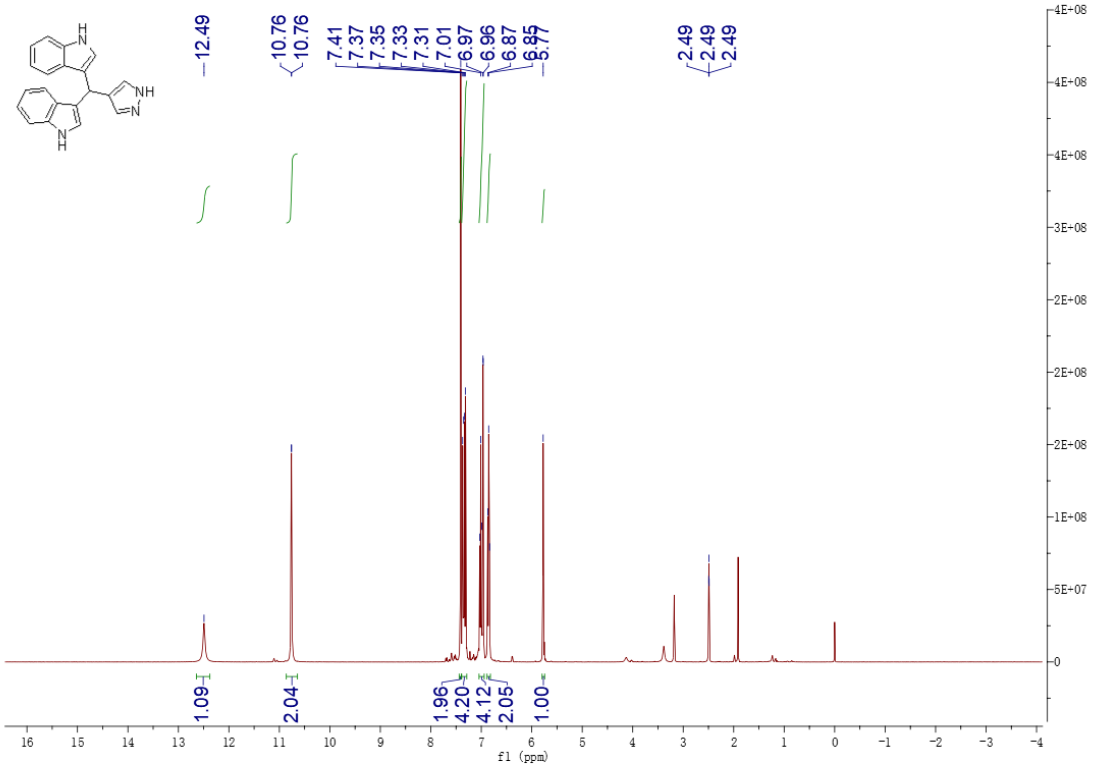

Figure S52. ^{13}C NMR spectrum (100 MHz, DMSO-d_6) of 14e

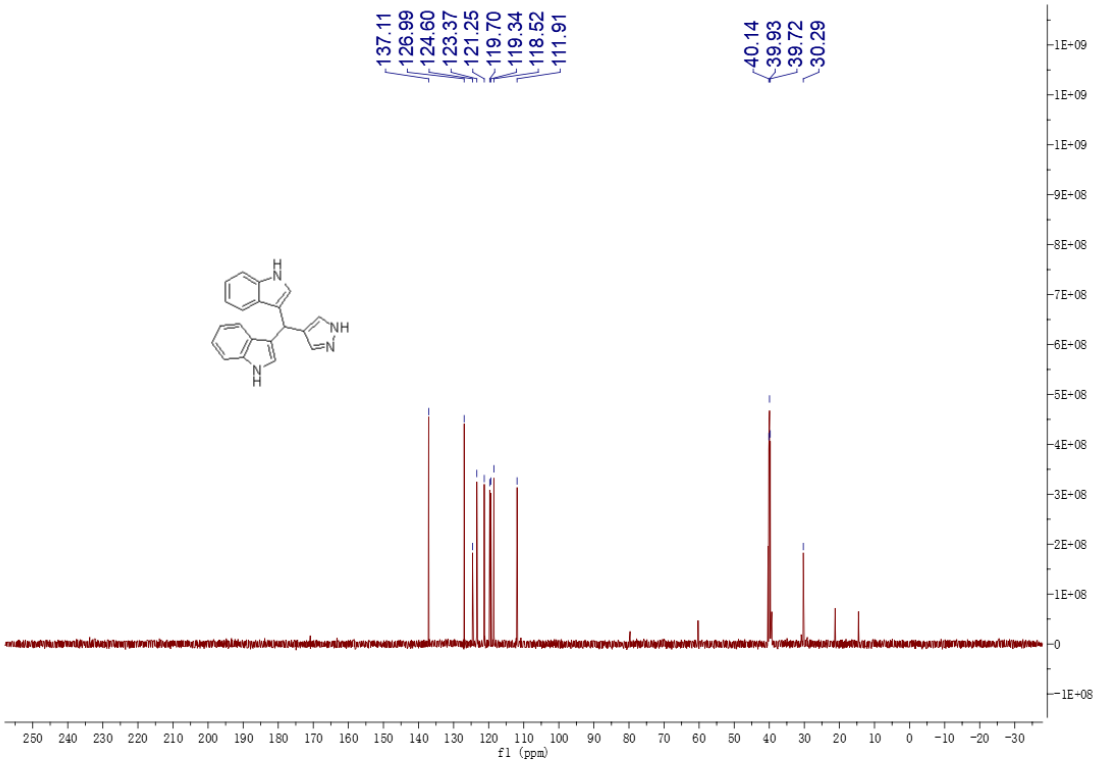

Figure S53. ^{1}**H NMR spectrum (400 MHz, DMSO-*d*$_6$) of 14f**

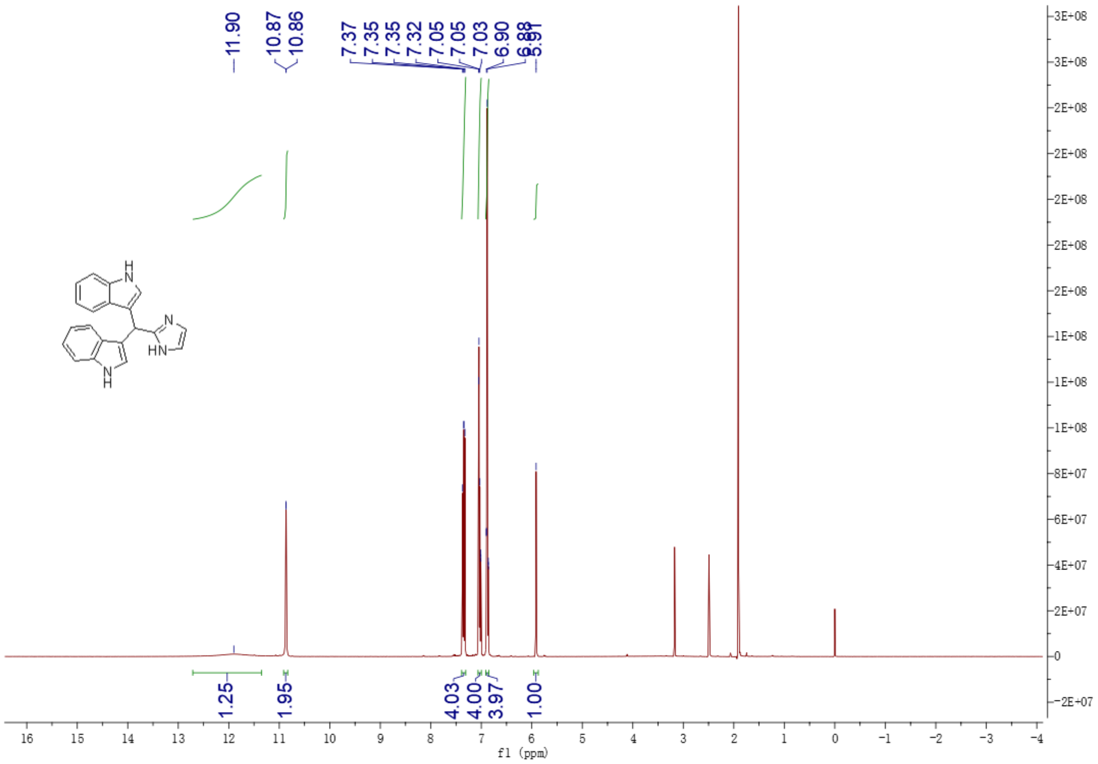

Figure S54. ^{13}**C NMR spectrum (100 MHz, DMSO-*d*$_6$) of 14f**

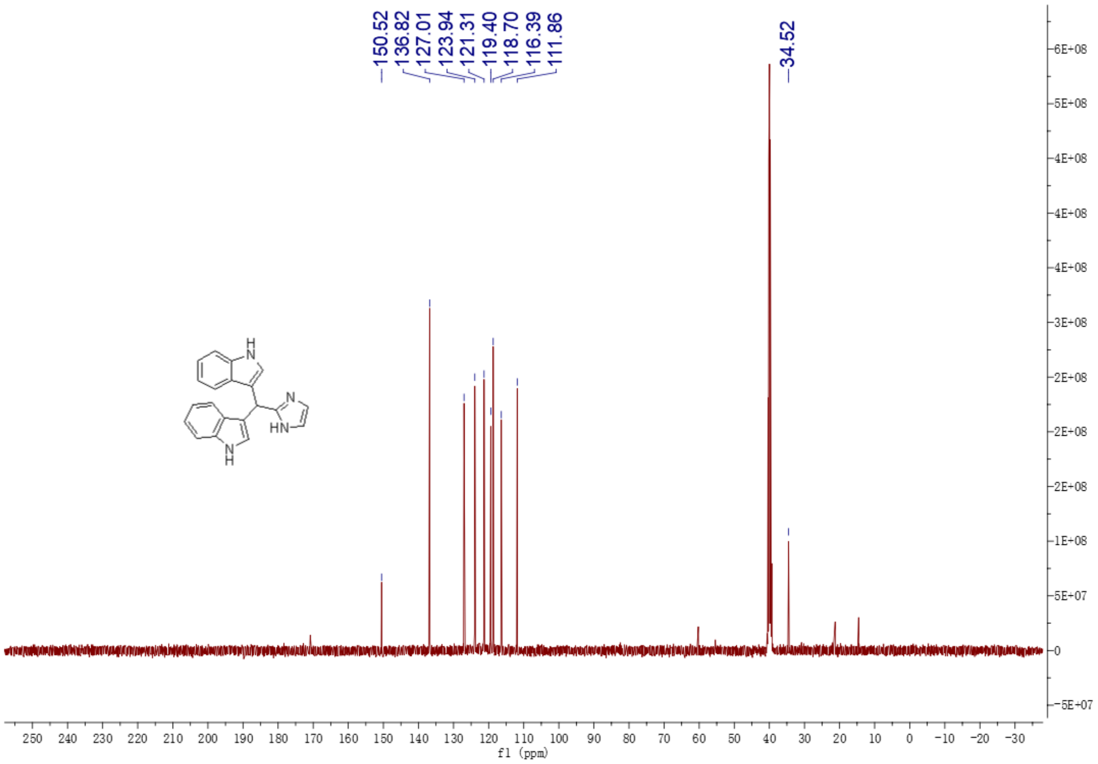

Figure S55. ^{1}**H NMR spectrum (400 MHz, DMSO-*d*$_6$) of 14g**

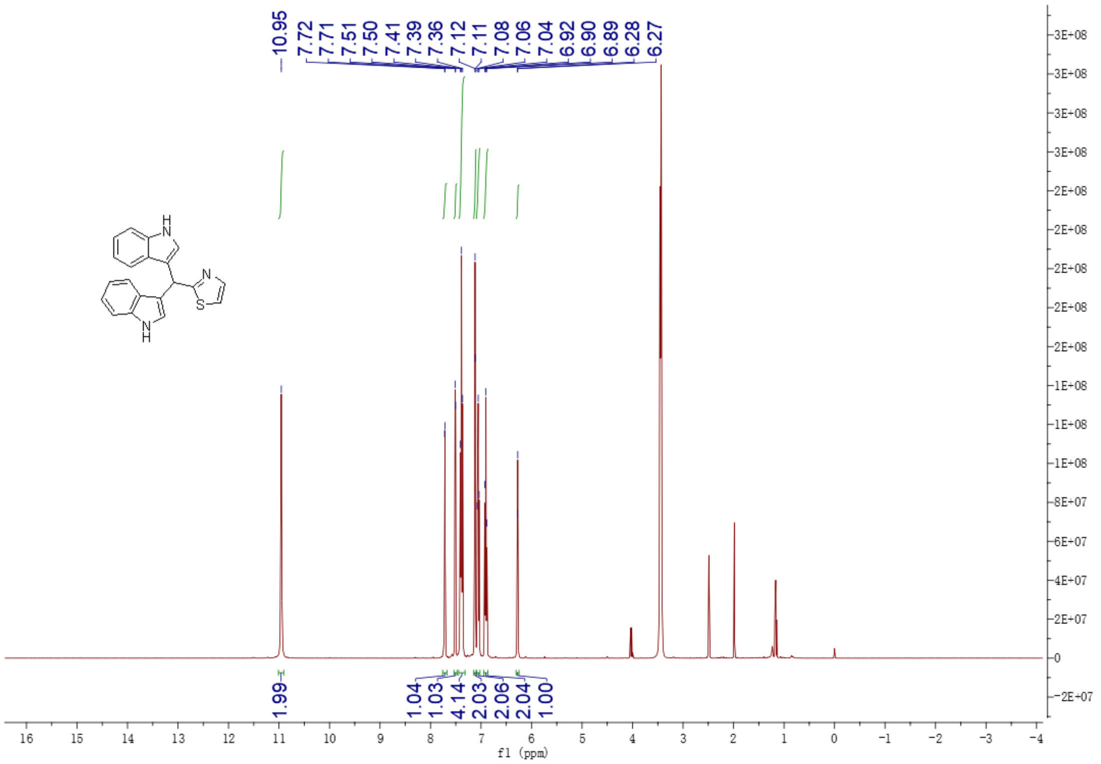

Figure S56. ^{13}**C NMR spectrum (100 MHz, DMSO-*d*$_6$) of 14g**

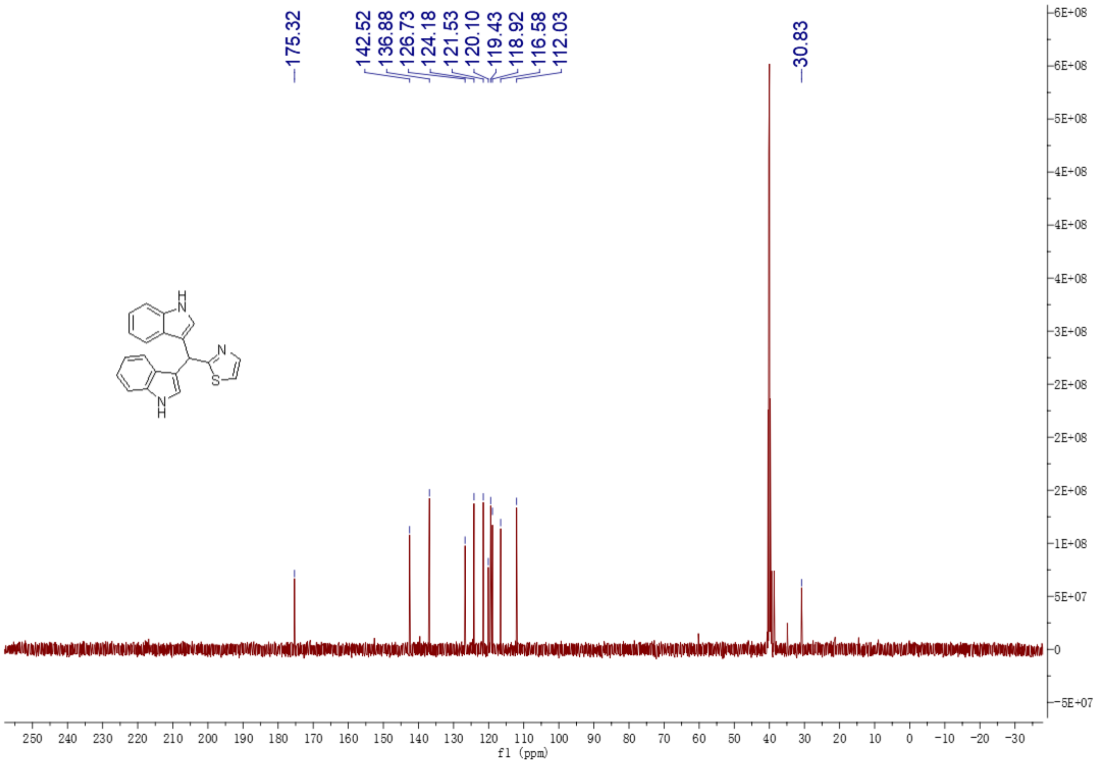

Figure S57. ^{1}H NMR spectrum (400 MHz, DMSO-d_6) of 15

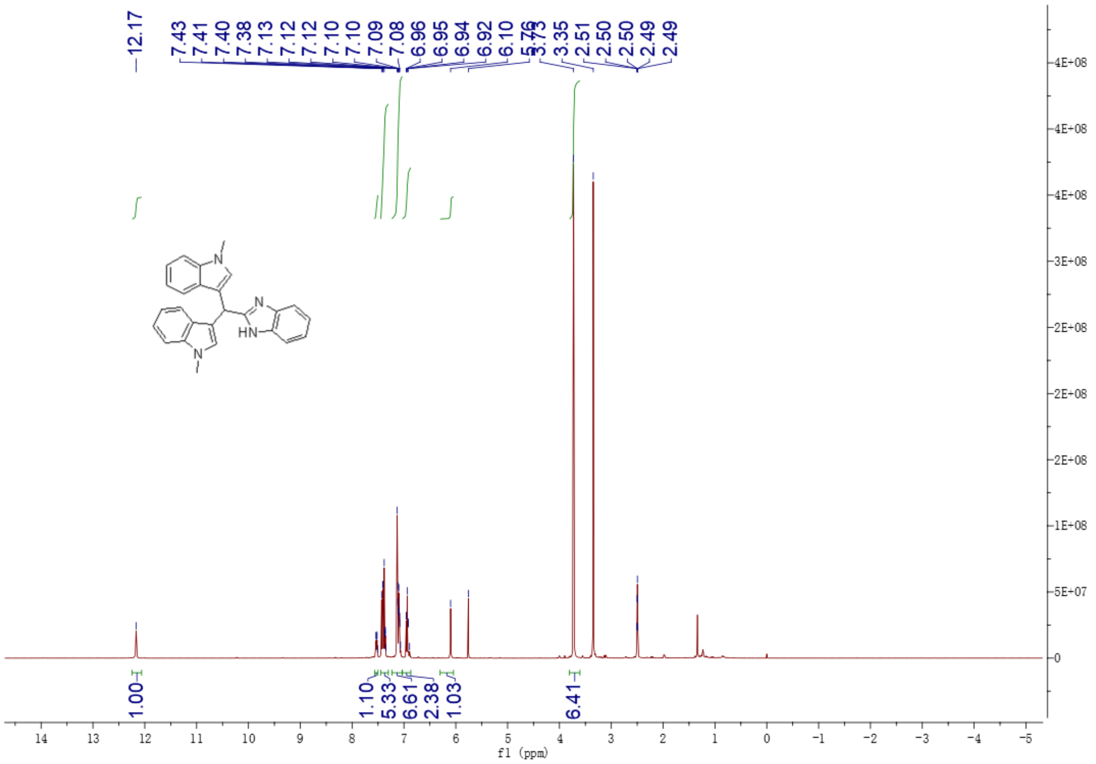

Figure S58. ^{13}C NMR spectrum (100 MHz, DMSO-d_6) of 15

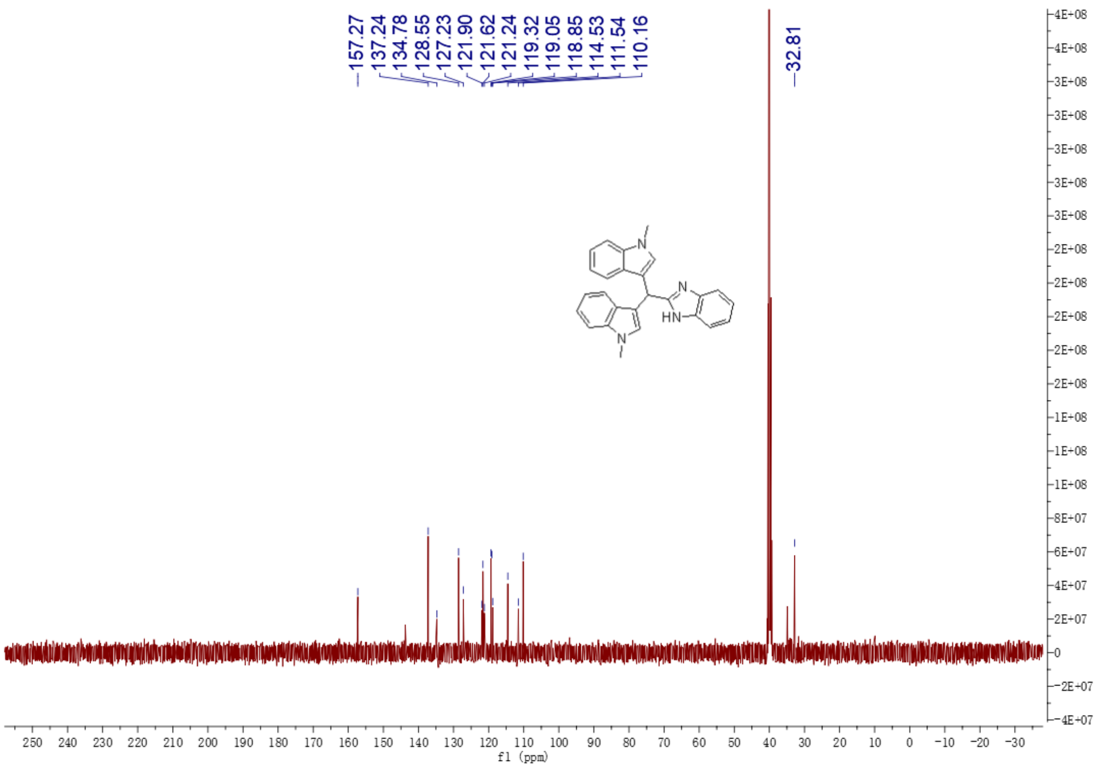

Figure S59. ^{1}H NMR spectrum (400 MHz, DMSO-d_6) of 16a

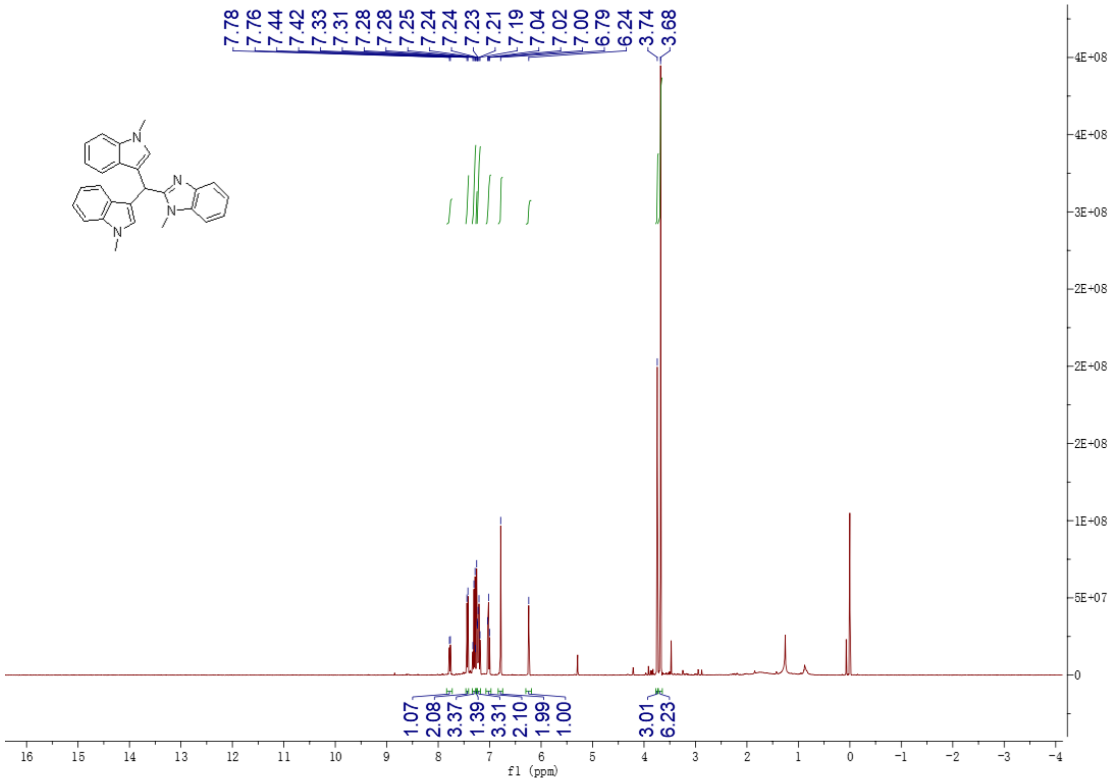

Figure S60. ^{13}C NMR spectrum (100 MHz, DMSO-d_6) of 16a

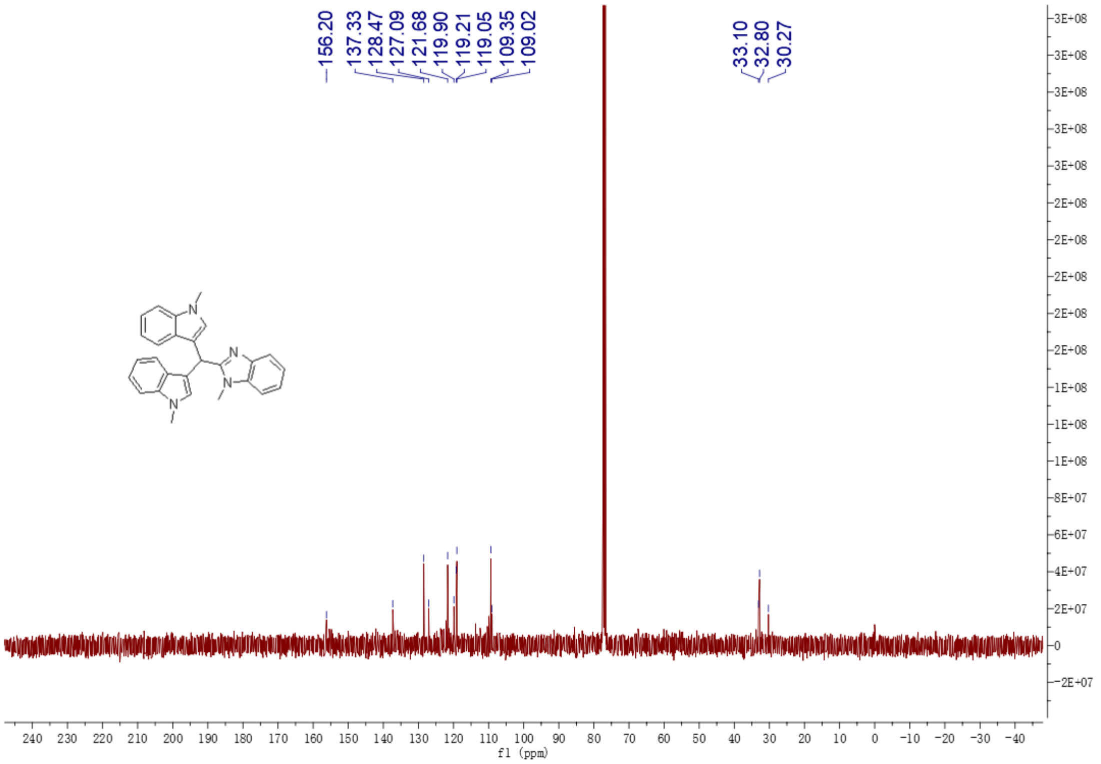

Figure S61. ^{1}H NMR spectrum (400 MHz, DMSO-d_6) of 16b

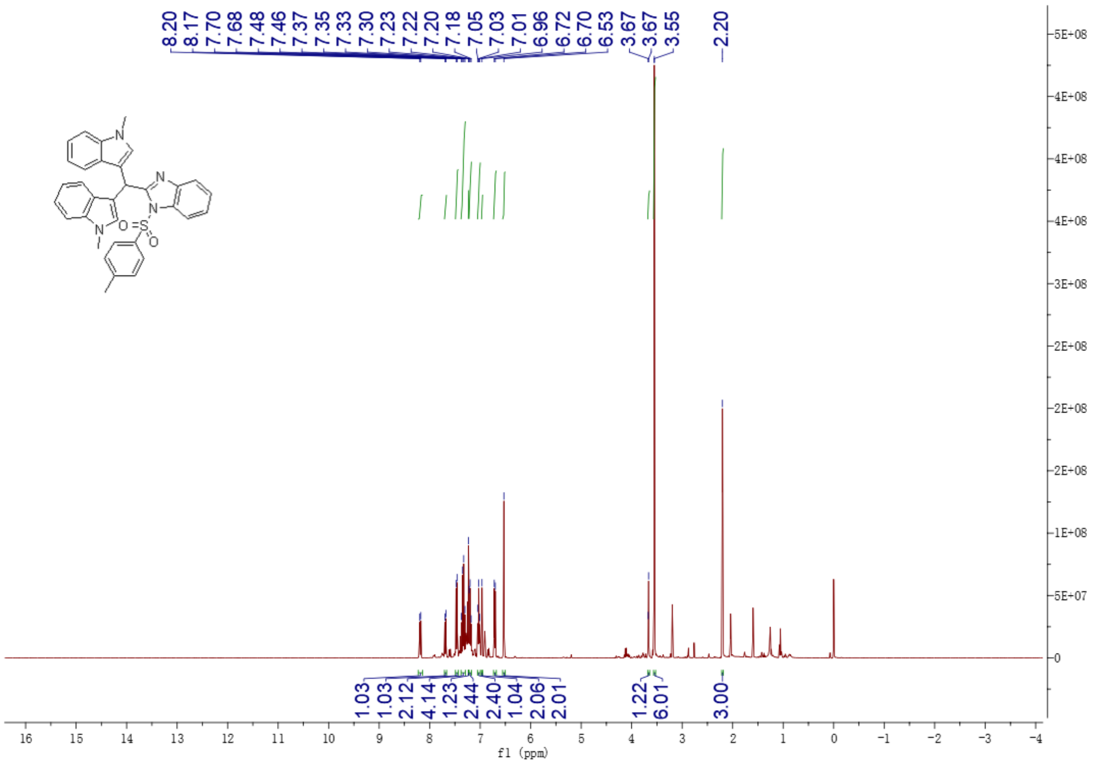

Figure S62. ^{13}C NMR spectrum (100 MHz, DMSO-d_6) of 16b

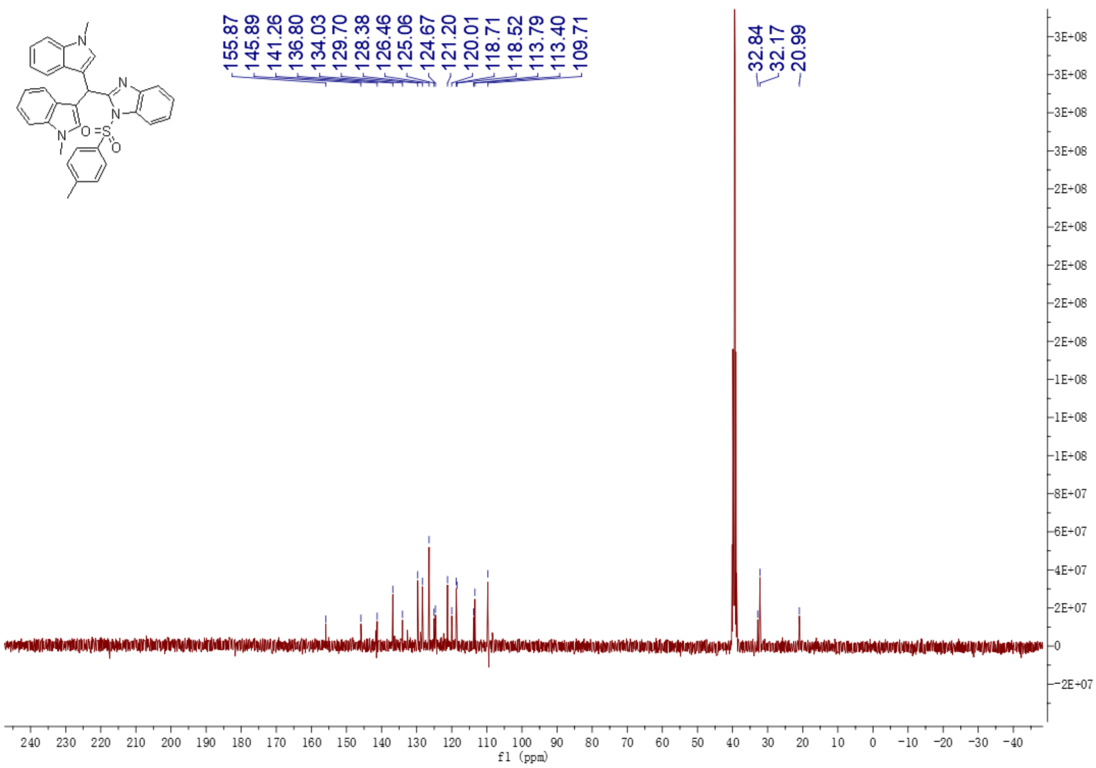

Figure S63. ¹H NMR spectrum (400 MHz, CDCl₃) of 16c

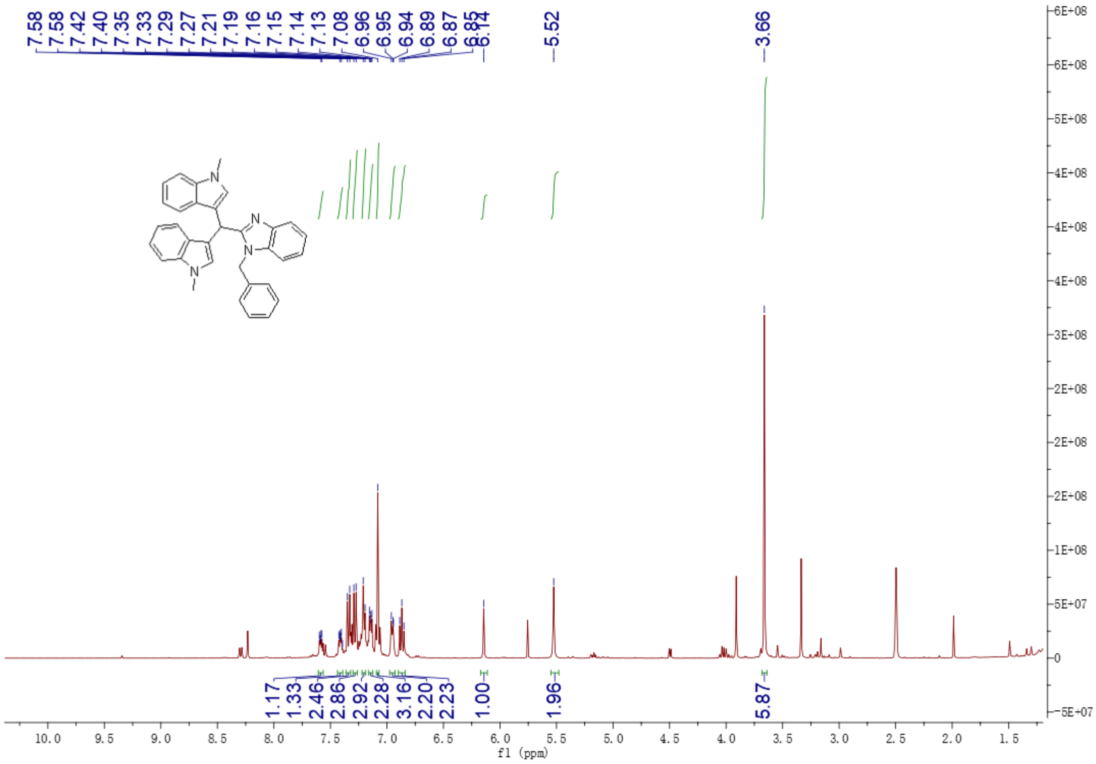

Figure S64. ¹³C NMR spectrum (100 MHz, CDCl₃) of 16c

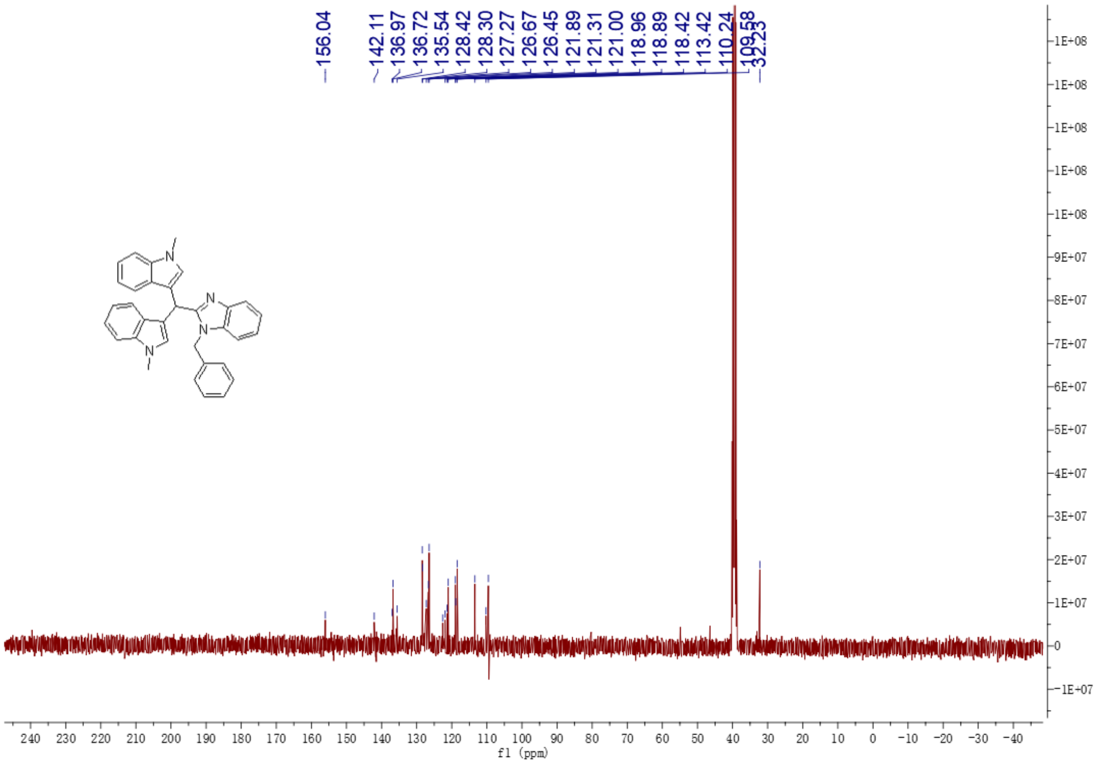

Supplement: Supplementary file 1 [file molecules-28-03032-s001.zip › molecules-2264099-supplementary.pdf]
